# Avian cecal microbiome response and resilience to Newcastle disease are dictated by breed background

**DOI:** 10.3389/fsysb.2025.1659648

**Published:** 2026-02-12

**Authors:** Aqsa Ameer, Farrukh Saleem, Ciara Keating, Farhan Afzal, Hamid Irshad, Khurshid Ahmed, Sadia Sattar, Umer Zeeshan Ijaz, Sundus Javed

**Affiliations:** 1 Department of Biosciences, COMSATS University Islamabad, Islamabad, Pakistan; 2 National Veterinary Laboratories, Ministry of National Food Security and Research, Islamabad, Pakistan; 3 Department of Engineering, Durham University, Durham, United Kingdom; 4 Livestock and Dairy Development Department Punjab Rawalpindi, Poultry Research Institute Rawalpindi, Rawalpindi, Pakistan; 5 Animal Sciences Institute, National Agricultural Research Center, Islamabad, Pakistan; 6 Water and Environment Research Group, University of Glasgow, Mazumdar-Shaw Advanced Research Centre, Glasgow, United Kingdom; 7 Department of Molecular and Clinical Cancer Medicine, University of Liverpool, Liverpool, United Kingdom; 8 Department of Microbiology, National University of Ireland, Galway, Ireland

**Keywords:** 16S rRNA, disease resistance, microbiota, Newcastle disease, poultry

## Abstract

A wide range of viral infections threaten the long-term sustainability of poultry production. Newcastle disease (ND), caused by Newcastle disease virus (NDV), is endemic in most Asian countries, including Pakistan, causing 50%–100% mortality in young and mature chickens. Some local chicken breeds show resistance to certain diseases and have greater survival probability. The chicken gut microbiome is linked to immune response against infections and to production performance parameters. The present study aims to comprehend disease resistance patterns in multiple chicken breeds with respect to gut microbial communities. Day-old Naked Neck, Black Australorp, Rhode Island Red, white layer, and broiler chicks were raised on an antibiotic-free diet in a semi-controlled setup. Vaccinated and non-vaccinated birds were challenged with NDV. Disease onset was delayed in breeds other than broilers, in which disease symptoms appeared at day 3 post-challenge with maximum severity and mortality. Other breeds, irrespective of vaccination, survived through the challenge period. Naked Neck showed the least variation in clinical features and growth parameters. A lower diversity in broiler groups with a significant decrease after NDV challenge was revealed by 16S rRNA amplicon sequencing of cecal DNA. Furthermore, broiler cecal core microbiome membership was found to be more variable than other breeds. Moreover, differentially abundant genera were observed across treatment groups and breeds with a similar effect on the predicted metabolic pathways, indicating varied energy metabolism responses. Shotgun metagenomics revealed a higher abundance of functional genes, including antimicrobial resistance (AMR) genes, stress genes, virulence genes, and amino acid degradation genes in the broiler NDV-infected group compared to the control group. The gut microbiota in chickens affects immunity to infections, health, and productivity. Compared to broilers, local chicken breeds, specifically Naked Neck, are found to have high immune competence in resisting ND while maintaining most performance metrics. Broilers show lower alpha diversity with an unstable core microbiome. Therefore, stable core microbiome maintenance may help the birds cope with the viral infection. The results support the farming of resistant chicken breeds over broilers to reduce production losses from NDV outbreaks.

## 1 Background

In many regions of the world, the poultry industry is expanding and becoming more industrialized. Increased urbanization, purchasing power, and population have all been major growth drivers (FAO, 2017). The poultry sector is one of the most important livestock subsectors in Pakistan, significantly contributing to its gross domestic product (GDP); it is growing 8%–10% annually, with over 8.5% share in exports in 2022–2023 (GOP, 2023; PRI, 2017). However, the industry has faced huge economic losses due to disease outbreaks (Deist et al., 2017). Newcastle disease virus (NDV) is among the five most prevalent viral infections in Pakistan (Umar et al., 2019). An increasing prevalence of Newcastle disease (ND) in Punjab Province, which is one of the highest production regions within the country, threatens the poultry sector (Munawar et al., 2013), with an estimated mortality of 50%–100% in chicken flocks (Copland and Alders, 2005). Depending on the viral strain, ND can exhibit a variety of clinical manifestations that impact host neurological, respiratory, and digestive systems (Brown and Bevins, 2017). The virus is highly contagious and spreads through direct contact with infected bird feces, secretions, and aerosols. It can survive for weeks within the environment, and transmission can also occur through contaminated machinery, feed, and clothing (Miller and Koch, 2013). Currently, there is no treatment for the disease, and the response of farmers is typically to cull infected birds, and potentially the entire flock, to control disease spread (Swayne et al., 2020). Prevention is also maintained through vaccinations against the virus. NDV vaccination, despite being commonly practiced, does not provide effective coverage due to genomic difference in the circulating strains and the possibility of vaccinated birds acting as viral reservoirs despite protective antibody titers (Rehmani et al., 2015). Moreover, commercialized poultry setups with inadequate biosecurity measures cannot prevent virulent NVD spread, despite flock vaccination (Moharam et al., 2019). Meanwhile, local chicken breeds have innate resistance to certain infections and greater survival probability due to diverse phenotypes/genotypes than commercial lines (Besbes, 2009; FAO, 2017; Sonaiya, 2008; Wong et al., 2017). Despite this, commercial lines are preferred due to enhanced performance parameters. It is unclear how Pakistani local breeds of chicken compare to broiler chickens in their response to NDV vaccination and infection.

We are becoming increasingly aware of the importance of the microbiome for host health. In chickens, a diverse and well-balanced gut microbiome has been linked to better growth, effective feed utilization, a robust immune system, disease resilience, and stress tolerance (Bajagai et al., 2024; Carrasco et al., 2019; Stanley et al., 2014; Yadav and Jha, 2019). In general, a myriad of resident bacteria in the intestines confers benefits to the intestinal physiology, including colonization resistance to pathogens (Berg, 1996; Deng and Wang, 2024; Hooper and Gordon, 2001; Khan et al., 2021). The chicken cecum is a functional part of its gastrointestinal tract and plays an important role in nutrition (Józefiak et al., 2004). The cecal microbiome has been shown to respond to disease infection by preventing colonization by pathogens; however, how it responds to NDV infection is not well studied.

This study aims to explore differences in chicken performance parameters in response to NDV infection in different chicken breeds to ascertain NDV resistant varieties. We assessed 125 birds from *Naked Neck*, *Black Australorp*, *Rhode Island Red*, *white layer*, and *broiler* breeds for disease morbidity, mortality, and performance parameters after NDV infection. The cecal microbiome was then acquired using amplicon sequencing of the 16S rRNA gene. Additionally, shotgun metagenomics analysis was carried out on broiler samples. We hypothesized that broilers would show reduced microbial diversity and that each breed would show a distinct microbial signature as well as a differential response to NDV infection. We further hypothesized that the cecal microbiome composition and function would be altered by NDV infection.

## 2 Methods

### 2.1 Experimental design

PREPARE guidelines (Smith et al., 2018) were followed for the study design and the optimization of the trial period and experimental procedures and to devise appropriate strategies for housing, waste disposal, and culling. ARRIVE guidelines (Percie du Sert et al., 2020) were followed for subsequent reporting. The protocol and procedures employed were reviewed and approved by the Ethics Review Board (ERB) at COMSATS University Islamabad (ERB No. CUI/Bio/ERB/4-21/17).

Details of the experimental trials, including preparation of the rearing site, the procurement of chicks, and the division of treatment groups, feed, and vaccinations are given in Ameer et al. (2024b). In brief, the study comprised five chicken breeds—three local breeds (Naked Neck, Black Australorp, Rhode Island Red) and two commercial breeds (white layer and broiler). For each breed, 25 mixed-gender day-old chicks (total n = 125) were reared for the experiment at the National Veterinary Laboratories (NVL), Islamabad, Pakistan. To minimize environmental confounding, birds from all breeds assigned to the same treatment group were housed in the same room under identical management conditions. Only the NDV-challenged groups were placed in separate biosafety rooms to prevent accidental transmission to control or vaccinated-only groups, as required by institutional biosafety guidelines. Birds were fed twice-daily on pre-estimated (in accordance with age) antibiotic-free feed. Birds were divided into four treatment groups (five birds per group): control, vaccinated, vaccinated challenged, and non-vaccinated challenged. Two chicks were sampled from each breed on day 1 to establish baseline cecal microbiome profiles. The remaining 20 birds were assigned to the four treatment groups (n = 5 per group). To buffer against potential morbidity or mortality during the trial, three extra birds were initially placed in the control group. Birds that remained healthy until the end of the experiment were retained, ensuring a final sample size of five birds per treatment group. Birds in the vaccination group were administered ND every month through commercially available live attenuated LaSota strain vaccine (VG/GA-AVINEW strain; Boehringer Ingelheim, Germany) according to the manufacturer’s instructions.

### 2.2 Virus propagation, Embryo infectious dose 50 (EID50) determination, and challenge dose calculation

For the challenge study, the details of NDV culture propagation, infectivity titer determination, and infection challenge are provided in Ameer et al. (2024a), Ameer et al. (2024b). In brief, NDV culture was prepared using viscerotropic-velogenic NDV (vvNDV) suspension by inoculating 8–9 day-old specific-pathogen-free (SPF) embryonated eggs [https://www.fao.org/4/ac802e/ac802e09.htm]. The success of the NDV culture was determined through the agglutination assay of red blood cells (RBCs) (Stear, 2005). The infectivity titer of the NDV suspension was determined through embryo infectious dose 50 (EID_50_) calculation (Reed and Muench, 1938). Birds were challenged orally at maturity with the viral suspension according to the calculated dose.

### 2.3 Morphological, growth, and hematological measurements

To evaluate the morphological and growth variations between different breeds and groups, measurements were taken at specific time points, starting from day 1. The weight, feed intake, height, shank length, beak length, beak width, and wingspan of the birds were measured using a weighing scale, plain scale, and digital Vernier caliper. The feed conversion ratio (feed intake to weight gain ratio) was measured in order to assess the flock’s feed efficiency. The effect of the vaccine and NDV challenge on the production performance of birds was documented to determine the breeds with high performance levels under stress. Before and after challenge, blood samples (two birds per group) were aseptically collected from the wing vein. An automated hematology analyzer was used for complete blood count (CBC) and differential cell count (DCC) following the manufacturer’s instructions. Elabscience®, USA ELISA kits were used to determine serum albumin, serum cholesterol, serum creatinine, serum globulin, serum glucose, uric acid, C-reactive protein (CRP), growth hormone, and insulin levels following the manufacturer’s instructions.

### 2.4 Hemagglutination inhibition (HI) assay

A HI test was performed to measure the presence and concentration of antibodies in vaccinated and NDV-challenged birds against the virus. The highest dilution of serum inhibiting the hemagglutination by consistent viral suspension is described as “hemagglutination inhibition” (HI) titer. NDV suspension was used as an antigen in the HI test. The presence and concentration of antibody is measured by its ability to inhibit the agglutination at various dilutions (Allan and Gough, 1974). The detailed protocol along with details of clinical symptoms of the disease and disease severity scoring are provided in the Supplementary Information.

### 2.5 DNA extraction, 16S rRNA gene sequencing, and shotgun metagenomics

The detailed methodology of DNA extraction, 16S rRNA gene amplicon sequencing (Ameer et al., 2024b), and shotgun sequencing has been provided by us previously (Ameer et al., 2024a). In brief, cecal samples were collected from each treatment group at day 1 (n = 2/breed) and maturity (n = 5/treatment group) and processed for DNA extraction using an Invitrogen PureLink™ Microbiome DNA Purification Kit following the manufacturer’s protocol (details in the Supplementary Information). The V4 region of the 16S rRNA gene using 515f and 806r primer sets was amplified using the pair-end method (Caporaso et al., 2011). DNA library preparation and quantification were validated using the Eco™ Real-Time PCR System. PCR was employed to selectively enrich DNA fragments containing adapters at both ends and to increase the overall yield of the library. Fragment size distribution was assessed using an Agilent 2100 bioanalyzer. Sequencing was carried out on an Illumina MiSeq platform using the v2 300-cycle reagent kit. Eight samples (four from broiler control; four from broiler non-vaccinated challenged group) were selected for shotgun metagenomic sequencing based on their exceptional behavior throughout the experimental period and sequenced through Illumina TruSeq.

### 2.6 Final set of parameters

For metadata, we used the following parameters with details provided in Supplementary Data Table S1: average weight, average feed intake, feed conversion ratio (FCR), hemagglutination inhibition (HI) titer, white blood cell (WBC) and red blood cell (RBC) counts, hemoglobin, platelets, hematocrit, mean corpuscular volume (MCV), mean platelet volume (MCH), mean corpuscular hemoglobin concentration (MCHC), mean platelet volume (MPV), platelet distribution width (PDW), heterophils, lymphocytes, monocytes, eosinophils, basophils, serum albumin, serum globulin, serum cholesterol, serum creatinine, serum glucose, uric acid, C-reactive protein, growth hormone, insulin, and height, shank length, beak length, beak width, and wingspan of the bird.

### 2.7 Bioinformatics

The details of bioinformatics pipelines used in the study to process 16S rRNA amplicon sequences and shotgun sequences are provided in Ameer et al. (2024a), Ameer et al. (2024b) and the Supplementary Material.

### 2.8 Statistics

Statistical analysis was carried out using R version 4.3.1 (2023-06–16).

#### 2.8.1 Assessing differences between treatment groups

For key metadata parameters that were continuous in nature, we autoscaled the data and used a nonparametric Kruskal–Wallis test to see if any of the parameters were statistically differential across different treatment groups. Afterward, we used a Random Forest classifier using R’s randomForest package (Liaw and Wiener, 2002) on the reduced set of differential features. This enabled us to use importance-sampling measures such as mean decrease in accuracy (MDA) and mean decrease in GINI (MDG) to rank the importance of each parameter in segregating different treatment groups. To visualize the rankings, bump charts were generated using “ggbump” (https://github.com/davidsjoberg/ggbump).

#### 2.8.2 Assessing treatment effects on recorded parameters

To estimate the treatment effect estimates (between Control and Challenged) for subgroups (vaccinated vs. others, broilers vs. others, Black Australorps vs. others, Rhode Island Reds vs. others, Naked Necks vs. others, and white layers vs. others) on the metadata parameters, R’s “subtee” package (Ballarini et al., 2021) was used.

#### 2.8.3 Microbial diversity

R’s vegan package (Dixon, 2003) was used for alpha and beta diversity analyses. For alpha diversity measures, after rarefying the abundance table to minimum a library-size count (9773 reads), we used (i) *Shannon entropy*, a commonly used index to measure balance within a community, and (ii) *Chao1 richness*, a nonparametric estimator of species richness which assumes that rare species carry information about the (unknown) number of unobserved species. We used R’s “aov()” function to calculate the pairwise analysis of variance (ANOVA) *p*-values, which were then annotated onto the alpha diversity figures. For beta diversity we used three different distance measures: (i) *Bray*–*Curtis distance* to visualize the compositional changes, (ii) *unweighted UniFrac distance* estimated using R’s phyloseq package (McMurdie and Holmes, 2013) to identify changes between samples in terms of phylogeny, and (iii) *hierarchical meta-storms* (HMS), which calculates the functional beta diversity distance in a hierarchical fashion propagating the KEGG orthologs (KOs) abundances upward to the pathways in a multi-level pathway hierarchy to give a weighted dissimilarity measure (Zhang et al., 2021). Principal coordinate analysis (PCoA) was used to visualize beta diversity dissimilarity. Additionally, the vegan package was used to perform PERMANOVA analyses to determine whether the microbial or functional community structures can be explained by different sources of variability. PERMANOVA was performed on a filtered set of parameters identified by the *redundancy analyses with forward selection* following recommendations from Vass et al. (2020) to retain only a partial subset of key parameters associated with the microbiome. In addition to the above-mentioned distance measures, we also employed weighted UniFrac distances in PERMANOVA by using the phyloseq package. For key parameters, we then fitted smooth surfaces of the covariates on an ordination plot (PCoA in this case) using penalized splines through the function “ordisurf()” in R’s VEGAN package. The method uses a generalized additive model by regressing the covariate as C ∼ S (Dim1, Dim2), where Dim1 and Dim2 are the ordination scores extracted from PCoA, and S() is a spline function. We only show those covariates where the model fits: p < 0.05.

#### 2.8.4 Core microbiome

To identify the core microbiome of each breed, we used the approach discussed in Shade and Stopnisek (2019). This first ranks OTUs using two metrics: site-specific occupancy (whether samples are grouped by different treatment groups: *control*, *vaccinated*, *vaccinated challenged*, and *non-vaccinated challenged*) and replicate consistency (whether the OTUs are consistent across replicates in each treatment group). After ranking the OTUs, the subset of core taxa was constructed incrementally by adding highly to minimally prevalent OTUs and then quantifying the contribution of the core subsets to beta diversity using the Bray–Curtis distance in the equation $C=1-\frac{BC_{core}}{BC_{all}}$ . The original authors specified a threshold at which the core subset construction stops—that is, where the addition of an OTU does not cause more than 2% increase in the explanatory value by Bray–Curtis distance. Independently, a neutral model (Burns et al., 2016) was fitted to the “S”-shaped abundance-occupancy distributions to inform those OTUs that are likely selected by the environment. These are obtained as those that fall outside the 95% confidence interval of the fitted model and are inferred to be deterministically assembled rather than neutrally selected. The taxonomy tree of the core microbiome across different breeds and treatment groups was drawn using R’s “metacoder” package (Foster et al., 2017).

#### 2.8.5 Differential taxa between treatments

To find genera and MetaCyc pathways that are significantly different between multiple conditions, we used the DESeqDataSetFromMatrix() function from the DESeq2 package (Love et al., 2014) with the adjusted *p*-value significance cutoff of 0.05 and log_2_ fold change cutoff of 2.

To find a minimal subset of genera that have changed with respect to continuous predictors considered in this study, we used CODA-LASSO regression (coda_glmnet() function), employing R’s coda4microbiome package (Calle et al., 2023). For the feature, we used the OTUs collated at genus level and the MetaCyc pathways in the CODA-LASSO model.

#### 2.8.6 Downstream analysis metagenomic assembled genomes

For the statistical analysis of data tables from the shotgun metagenomics dataset, R’s vegan and coda4microbiome packages were used to obtain diversity estimates and CODA-LASSO regressions, respectively. Additionally, to determine the relationship between individual microbes and infection, we used the generalized linear latent variable model (GLLVM) within R’s “gllvm” package (Niku et al., 2019). Approximation to the log-likelihood was done through variational approximation (VA), with final sets of parameters in the “glvmm()” function being family = “negative.binomial,” method = “VA,” and “control.start = list (n.init = 7, jitter.var = 0.1)” that resulted in convergence of the algorithm.

## 3 Results

### 3.1 ND susceptibility upon NDV challenge

Broiler chickens showed the quickest onset of ND, with disease symptoms appearing at day 3 post-challenge, in comparison to disease onset at day 7 post-challenge for all other breeds. ND symptoms included respiratory distress, watery diarrhea, anorexia, lethargy, and mucus discharge. Disease severity scores were assigned based on these estimates (Supplementary Table S1). Hemorrhagic cecal tonsils, the intestine, and proventriculus were also observed during post-mortem inspection. Overall, vaccinated birds showed mild symptoms such as low feed intake and depression compared to the non-vaccinated ones. Maximum disease severity was observed in the non-vaccinated broiler group, in which all birds expired except one (80% mortality). One non-vaccinated Naked Neck chicken also expired, but that mortality was linked more with agonistic interactions. In general, all other breeds, whether vaccinated or not, survived the study period.

### 3.2 Morphological, performance, and hematological parameters in response to NDV vaccination and challenge

**FIGURE 1:**
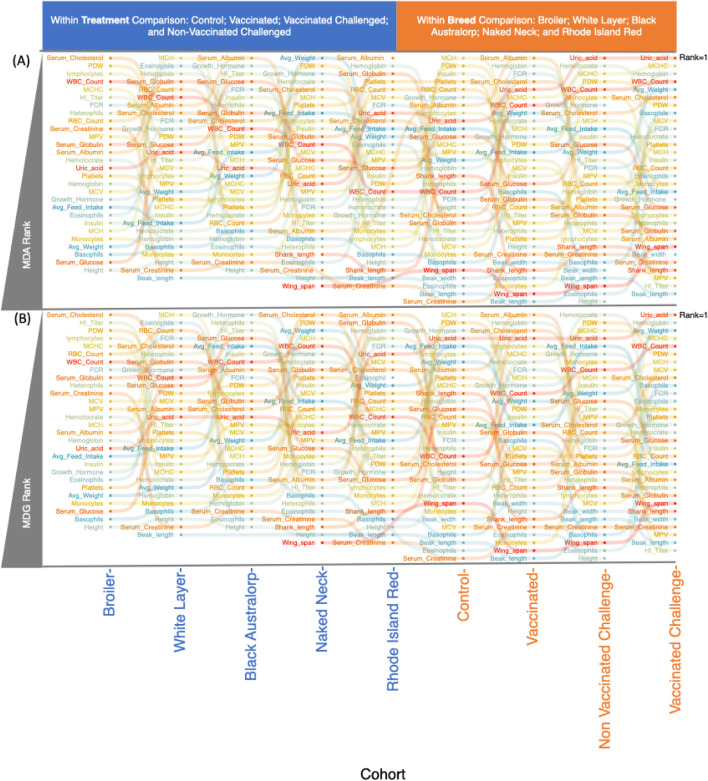
Bump plot to explore ranking of covariates returned from fitting Random Forest classifier (Supplementary Figures S1–S9) on the effects of treatment groups within a given breed (left side; blue axis text) and the effect of breeds within a given treatment group. For within-group comparison for cohorts given in the x-axis, the groups are listed on the top. The rank decrease from top to bottom, with higher rank suggesting parameters with more discriminating power. **(A)** Ranking returned based on the mean decrease in accuracy (MDA) importance measure; **(B)** ranking returned based on mean decrease in GINI (MDG). Rank increases downward for both measures, with higher ranks suggesting those parameters that cause larger class separations (whether breed or treatment).

We also observed changes in association with different treatment groups by applying the Kruskal–Wallis test followed by ranking through Random Forest classifier. As expected, these responses were varied for different breeds. Serum cholesterol, platelet distribution width (PDW), white blood cells (WBCs), lymphocytes, heterophils, and HI titer increased after NDV vaccination or challenge. Meanwhile, serum glucose, serum globulin, and growth hormone decreased in vaccinated groups, and platelets, hematocrit, average weight, and red blood cell (RBC) count decreased after challenge. Similarly, different breeds showed varied responses with respect to these parameters (Figure 1; Supplementary Figures S1–S9).

Subtee analysis calculated the challenge effect to find the subgroup (whether a member of broiler, Naked Neck, Black Australorp, Rhode Island Reds, white layer, or vaccination group) most drastically affected after challenging the birds with respect to the parameters of interest in terms of increase or decrease in that parameter. In terms of growth performance, the most affected group was broiler, which showed the greatest decline in average weight, feed intake, and growth hormone levels after viral challenge. In terms of morphological parameters such as height, shank length, beak length, beak width, and wingspan, the most affected group was Rhode Island Red. Considering the hematological parameters as a measure of resilience, Naked Neck turned out to be the least affected breed, despite high HI titer upon challenge. Other breeds including While Layer and Rhode Island Red showed substantial change in factors such as RBC count, monocytes, heterophils, and basophils after challenge (Supplementary Figure S10). Additionally, a LASSO regression model was applied to determine regulation of all the covariates as a result of NDV vaccination and infection (Supplementary Table S3).

### 3.3 Gut microbial and functional diversity in response to vaccination and challenge in different breeds

**FIGURE 2:**
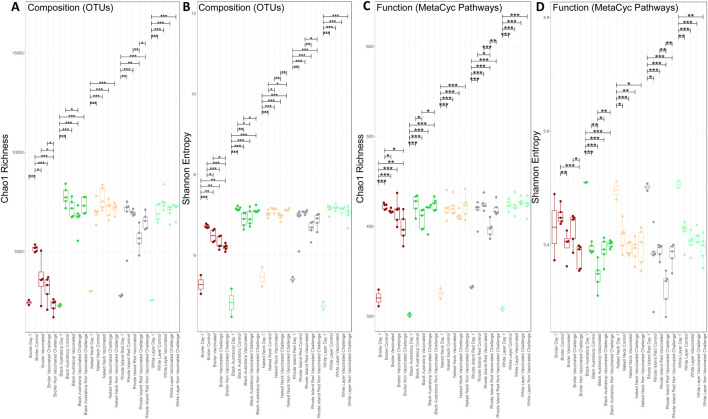
Alpha diversity comparison of bacterial OTUs and predicted MetaCyc pathways (PICRUSt2). **(A)** Chao1 richness of bacterial OTUs, **(B)** Shannon entropy of bacterial OTUs, **(C)** Chao1 richness of predicted MetaCyc pathways, and **(D)** Shannon entropy of predicted MetaCyc pathways. Lines connect samples according to ANOVA; solid lines indicate statistically significant differences with significance levels shown a *p < 0.05, **p < 0.01, and ***p < 0.001.

We found that day-old chicks have lower alpha diversity than mature chickens, suggesting an increase in species richness with age. We observed a marginal decrease in alpha diversity after vaccination compared to control; however, this result was not significant. A significant decrease in diversity was also observed in Black Australorp, Rhode Island Red, and Naked Neck after NDV challenge. Species richness was significantly lower in broilers than in all other breeds. The broiler control group had the highest alpha diversity values, with a significant decrease observed after NDV challenge. We also observed a marginal decrease in species richness after vaccination. Similarly, a marginal decrease in species richness was observed in the Black Australorp and Rhode Island Red groups after NDV challenge. Meanwhile, no significant change in species richness was observed in the case of Naked Neck and white layer after challenge (Figures 2A,B).

A decrease in predicted functional richness was observed after NDV challenge compared to the control groups in all breeds (Figure 2C). A significant decrease in Shannon entropy was observed in non-vaccinated challenged broilers compared to controls. For other breeds, a marginal decrease in Shannon entropy was observed in the vaccinated and challenged groups (Figure 2D).

**FIGURE 3:**
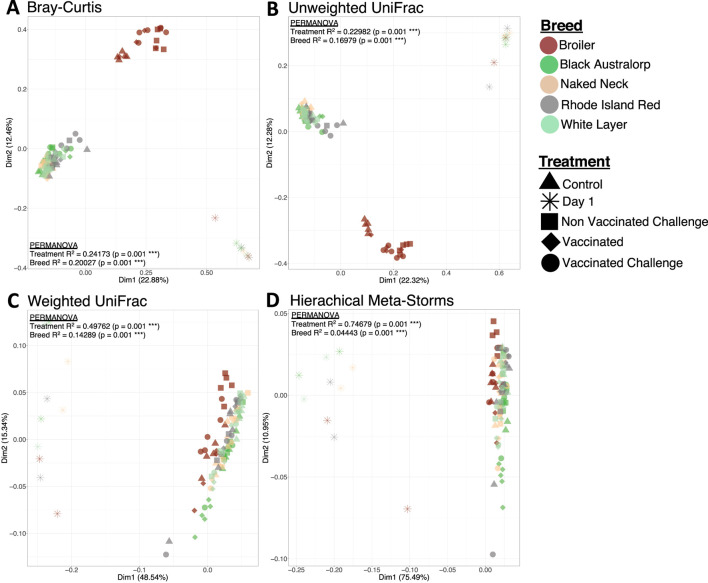
Beta diversity represented by principal coordinate analysis (PCoA) plots, with each axis showing the percentage variability explained by that axis. We used four different distance measures: **(A)** Bray–Curtis distance to show differences in composition, **(B)** unweighted UniFrac distance to show differences in phylogeny, **(C)** weighted UniFrac to show differences in both composition and phylogeny, and **(D)** hierarchical meta-storms to show differences in metabolic function. PERMANOVA statistics utilizing these distance measures are shown to suggest whether there are significant differences between treatment (*day 1*, *control*, *vaccinated*, *non-vaccinated challenged*, and *vaccinated challenged*) and breed (*broiler*, *Black Australorp*, *Naked Neck*, *Rhode Island Red*, and *white layer*), with the R^2^ value explaining percentage variability (scaled to 1).

Beta diversity indices indicated clear and significant shifts in the chicken cecal microbial structure with age, where day-old chicks clustered away from mature chicken samples in all the breeds. The broiler treatment groups formed a cluster distinct from all other breeds. In contrast, all other breeds irrespective of vaccination or challenge status clustered together (Figures 3A,B). Combining both abundance and phylogeny in weighted UniFrac (Figure 3C) led to all breeds overlapping, although day-old chicks still remained distinct from mature birds. Similar results (no distinguished clustering) were observed in the case of Hierarchical Meta-Storms, indicating functional redundancy in the chicken cecal microbiome irrespective of breed (Figure 3D).

We found that chicken breed was associated with the most variability in microbial composition (27%), weighted phylogeny (31%), and function (23%). Meanwhile, only 6% variability in microbial composition and 9% variability in function were attributable to NDV vaccination or infection. In addition, factors such as FCR, HI titer, growth hormone, WBC count, RBC count, and heterophils significantly contributed to the community indices, indicating microbiome-associated changes in host physiology and immune response (Supplementary Table S4). The results are further validated through a generalized additive model in the form of PCoA plots using penalized splines (Supplementary Figure S16).

### 3.4 Cecal core microbiome membership in different chicken breeds

**FIGURE 4:**
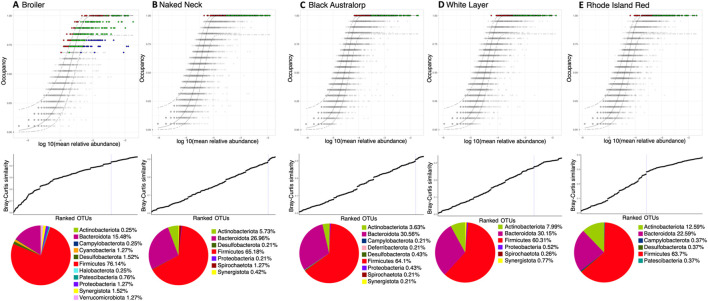
Core microbiome of all chicken breeds: **(A)** broiler, **(B)** Naked Neck, **(C)** Black Australorp, **(D)** white layer, and **(E)** Rhode Island Red. We have incorporated a treatment-specific occupancy model (multiple replicates for each treatment: *control*, *vaccinated*, *vaccinated challenged*, and *non-vaccinated challenged*, respectively). Once we obtained the OTU rankings depending on occupancy within these treatment groups as well as their replicate consistency, Bray–Curtis similarity is calculated for the whole dataset and then also for only the top-ranked taxa. The contribution of the top-ranked taxa is divided by the total Bray–Curtis similarity to calculate a percent contribution of the prospective core set to beta diversity. The next-ranked taxon is added consecutively to find the point in the ranking at which adding one more taxon offers diminishing returns on explanatory value for beta diversity; it is shown in the middle row. The dotted line represents the “last 2% decrease” criterion where OTUs are incorporated into the core subset until there is no more than a 2% decrease in beta diversity. Independently, a neutral model is fitted to the dataset for each breed. Combined with the core microbiome, the top row then represents the core OTUs that are neutral, falling within the 95% interval confidence intervals shown in green, while non-neutral core OTUs with observed frequency above the predicted frequency from the neutral model (selected by the host) are shown in red. Those core OTUs with observed frequency below the predicted frequency from the neutral model (selected by dispersal limitation) are shown in green. All other OTUs are grayed out. The bottom row shows a pie-chart of core OTUs per breed and their taxonomic identity (at phylum level). The abundance of core OTUs for each breed and different treatments are shown in Supplementary Figures S1–S5.

**FIGURE 5:**
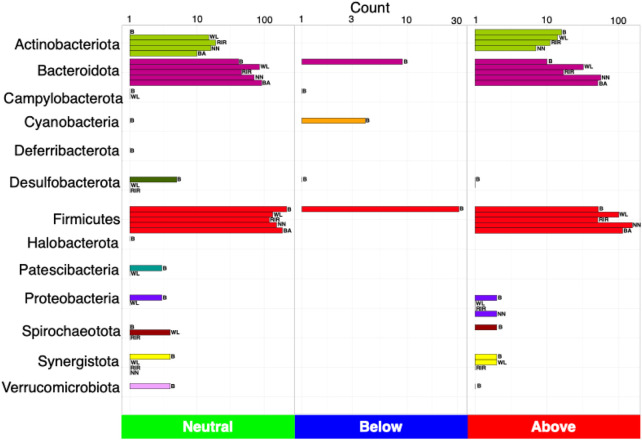
Count of the number of OTUs detected as neutral, below (selected by dispersal limitation), and above (selected by host), classified at phylum level. Legends are as follows: B, broiler; WL, white layer; RIR, Rhode Island Red; NN, Naked Neck; BA, Black Australorp.

We next used a dynamic core microbiome inference strategy coupled with neutral modeling to determine the core subset of OTUs and whether they are selected by the host (Figures 4A–E). All breeds other than broiler showed a stable core reaching an occupancy of 100% of the respective samples. A few core taxa were selected by the influence of a breed’s gut environment. In case of broiler, core membership was more variable, with a minimum occupancy threshold computed to be ∼69%. In particular, taxa that fall above the neutral model prediction are in a higher occupancy than expected by their mean abundance in the dataset, and taxa that fall below the prediction are in higher abundance than expected by their occupancy. This means that for the cecal microbial communities of local breeds and white layer, host genetics might have a role in taxa selection. Meanwhile in broiler, in addition to host (and/or the environment) effect, some core taxa were dispersal limited (Figure 5). Phylum Firmicutes dominated the core subset, with a maximum proportion in broiler (76.14%) and minimum in white layer (60.31%). The second major core phylum was Bacteroidota, with a maximum proportion in Black Australorp (30.56%) and minimum in broiler (15.48%). Phylum Actinobacteriota dominated in Rhode Island Red, with a minimum proportion of core subset in broiler. Core OTUs of Campylobacterota were present in broiler, Rhode Island Red, and Black Australorp but were absent from Naked Neck and white layer. Desulfobacterota core OTUs were found in all breeds except white layer. Proteobacteria core OTUs were found in all breeds except Rhode Island Red. Core OTUs belonging to Halobacterota and Verrucomicrobiota were only found in broiler. The taxonomic details of core OTUs recovered for all breeds (representing collated abundances of all groups merged) and treatment groups (specific abundances) are provided in Supplementary Figures S11–S15.

### 3.5 Influence of chicken breed on the cecal microbiome

DESeq2 was applied to find log2 fold differentially abundant genera and MetaCyc pathways between the groups. In general, more genera were differentially abundant across treatment groups (vaccinated and challenged groups compared to control) than breeds. *Corynebacterium*, *Dietzia*, *Brevibacterium*, *Yaniella*, *Aerococcus*, *Facklamia*, *Aliicoccus*, and *Staphylococcus* abundances were increased in control groups, suggesting their decreased abundance after NDV vaccination or infection. In addition, some genera such as *Paenochrobactrum*, *Pseudochrobactrum*, *Clostridium_sensu_stricto_1*, *Escherichia–Shigella*, *Akkermansia*, *Frisingicoccus*, and *Solobacterium* were abundant in vaccinated or infected groups. *Kocuria* abundance increased in the vaccinated groups. Moreover, some genera were differentially abundant in specific breeds, such as *Collinsella* and *Chlamydia* in Naked Neck, *Alloprevotella* in Black Australorp, and *Butyricicoccus*, *Roseburia*, and *Intestinimonas* in broiler groups. Similarly, *Oerskovia*, *Romboutsia*, *Ochrobactrum*, and *Microbacterium* in white layer and *Megamonas*, *Gulosibacter*, and *Lachnoclostridium* in Rhode Island Red groups were differentially abundant. Interestingly, *Campylobacter* (a common zoonotic pathogen) was found to be upregulated in broilers upon NDV infection (Supplementary Table S5).

A total of 85 MetaCyc pathways were found to be differentially abundant using a permutation combination of all pairwise groups. We observed breed and treatment specific effect on the predicted metabolic pathways, indicating varied energy metabolism responses in different groups. Interestingly, a majority of the differentially observed pathways were found in the broiler, white layer, and Rhode Island Red groups. These include CATECHOL-ORTHO-CLEAVAGE-PWY, LIPASYN-PWY, P101-PWY, PWY-5417, PWY-5431, and PWY-6992. A few pathways were found to be selectively enriched or decreased in Naked Neck and Black Australorp. Overall, the predicted functional profiles were significantly different between control, vaccinated, and challenged groups of each breed, with the majority of pathways upregulated in the control groups. These included pathways associated with carbon and energy utilization, fatty acid and lipid degradation, protein biosynthesis, aromatic compound degradation and amide/amidine/amine, and polyamine biosynthesis. Interestingly only select pathways involved in protein degradation/modification, creatinine degradation (PWY-4722), and protein N-glycosylation (PWY-7031) were upregulated in the non-vaccinated infected groups. Protein N-glycosylation was specifically present in *Campylobacter* and involved in modifying proteins with oligosaccharide structures in response to viral infections. In addition, the pseudaminate biosynthesis (PWY-6143) pathway, which is involved in pathogen colonization through flagella assembly modulation, and mannosylglycerate biosynthesis (PWY-5656) involved in stress adaptation by halophilic bacteria and L-arabinose degradation IV (PWY-7295) were upregulated only in the non-vaccinated infected broiler and Black Australorp groups. Meanwhile, the isopropanol biosynthesis (PWY-6876) pathway was only upregulated in the Rhode Island Red infected group. Demethylmenaquinol-6 biosynthesis II (PWY-7373) was upregulated in the Black Australorp non-vaccinated infected group, which is involved in vitamin K2 synthesis and is important for coagulation (Supplementary Table S6).

### 3.6 Impact of NDV infection on the structure and function of cecal microbial communities

**FIGURE 6:**
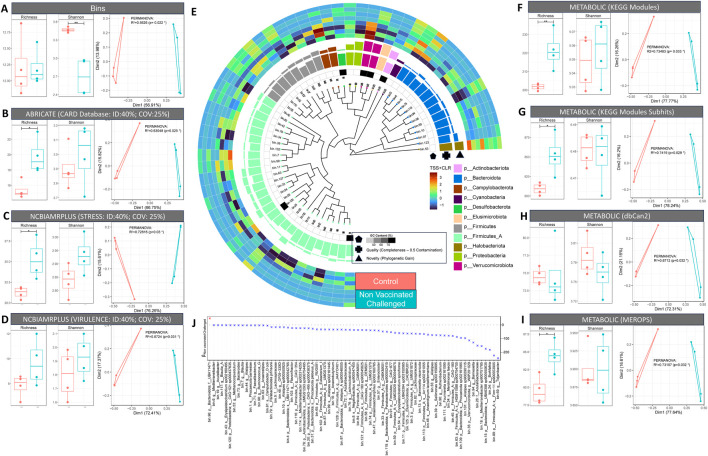
Shotgun metagenomics results. **(A–D, F–I).** Taxonomic and functional diversity (alpha and beta diversity) comparison of control and non-vaccinated challenged samples. For alpha diversity plots (richness and Shannon entropy), ANOVA significances are as follows: p < 0.001 (***), p < 0.01 (**), p < 0.05 (*). For beta diversity plots, principal coordinate analysis (PCoA) is used along with PERMANOVA showing percentage variability between the control and non-vaccinated challenged groups. **(E)** Phylogenetic tree of MAGs recovered via GToTree using 25 bacterial and archaeal specific single copy genes. The tree also features G-C content, quality index (genome completion: 5 × genome contamination), and novelty (represented by phylogenetic gain (PG) values calculated using the GTDB toolkit). **(J)** Generalized linear latent variable model (GLLVM) where the abundance of individual MAGs is regressed against the grouping variable (control; non-vaccinated challenged) with beta coefficients represented by either positive (red) or negative (blue) association in comparison to control (reference).

In agreement with the amplicon sequencing results, broiler control samples showed significantly higher alpha diversity but no marked difference in species richness between infected and control groups. Similarly, beta diversity was observed to be different with 56.26% variability (PERMANOVA using Bray–Curtis distance; p = 0.022) between the groups (control and challenged) (Figure 6A). We further explored the abundance of antimicrobial resistance (AMR), stress, and virulence genes as well as enzymes associated with major metabolic functions. The infected (challenge) group possessed a higher abundance of functional genes, including AMR, stress, and virulence, than the control group. The healthy ceca were enriched for genes involved in carbohydrate metabolism. In contrast, amino acid degradation genes were enriched in the infected group (Figures 6A–D,F–I). The majority of detected antimicrobial resistance genes were involved in transposon-mediated antibiotic inactivation. Furthermore, the disinterred virulence and stress genes were mainly involved in adherence to epithelial cells and AMR, respectively (Supplementary Figures S17–S26).

In general, we observed more genera to be differentially abundant across treatment groups (vaccinated and challenged groups compared to control) than across breeds. *Corynebacterium*, *Dietzia*, *Brevibacterium*, *Yaniella*, *Aerococcus*, *Facklamia*, *Aliicoccus*, and *Staphylococcus* showed decreased abundance after NDV vaccination or infection. On the other hand, *Paenochrobactrum*, *Pseudochrobactrum*, *Clostridium_sensu_stricto_1*, *Escherichia–Shigella*, *Akkermansia*, *Frisingicoccus*, and *Solobacterium* were more abundant in vaccinated or infected groups*. Kocuria* abundance was high in the vaccinated groups. Since broiler groups stood out in terms of disease severity, microbial diversity and differential analyses, we selected two broiler groups (control and non-vaccinated challenged) for shotgun metagenomics to assess responses to NDV infection. Firmicutes A and Firmicutes, as per GTDB-TK taxonomy, were the most abundant phyla present in the broiler ceca, with Clostridia and Bacilli as the major classes within this. Bacteroidota was the second most abundant phylum, with Bacteroidia as the major class within this. We also found some novel Firmicutes*,* Alphaproteobacteria, Gammaproteobacteria, and Actinobacteriota. Furthermore, the majority of the Firmicutes were anaerobic Clostridia such as *Anaerotignum lactatifermentans*, *Sellimonas sp002159995*, and *Fournierella sp002161595* present in the infected group. In contrast, the majority of the commensal Bacteroidota belonging to the class Bacteroidia, including *Parabacteroides sp900552415*, were enriched in healthy birds (Figure 6E). Just one MAG (genus *Tidjanibacter*) showed a positive association with NDV infection (Figure 6J).

## 4 Discussion

Newcastle disease (ND) poses a constant threat to the poultry industry (Ganar et al., 2014; Orsi et al., 2009; Yang et al., 2017). ND induces a high mortality which significantly reduces poultry production. In the developing world, poultry is an essential nutrition and income source, and thus losses from ND can be devastating (Charkhkar et al., 2024). Vaccines are widely used to protect chickens but have low efficacy due to inappropriate vaccine regimes, poor biosafety practices, and antigen divergence between strains (Elbestawy et al., 2023).

The gastrointestinal tract of chickens is colonized by an intricate network of microbial communities (Ley et al., 2008). Gut microbial communities play an important role in the production performance and overall health of the birds (Shang et al., 2018). It has been reported that the typical microbiome profile affects the development of viral illnesses (Mizutani et al., 2022) and that certain bacterial metabolites have a strong protective effect (Domínguez-Díaz et al., 2019; Feng et al., 2023). In order to comprehend the pathophysiology underlying viral illnesses, it is crucial to clarify the interactions between viral infections and microbiota. Symbiotic interaction between host and native microbiota is important in the development of viral infections. First, cell attachment, the initial stage of a viral infection, is physically impeded by commensal bacteria present in the mucosa (Bron et al., 2017). The antiviral action is facilitated by the continual activation of the host immune system by substances derived from certain gut bacteria (Robinson and Pfeiffer, 2014). Furthermore, it is thought that “cross talk,” which includes material or physical transfers between the host cell and the microbiota on the cell epithelium, plays a significant role in infection progression. This has an impact on intracellular processes as well such as in controlling the expression of viral genes in infected cells (Mizutani et al., 2022). Furthermore, gut microbiota may influence interferon responses, which in turn indirectly influences virological outcomes (Wirusanti et al., 2022). At the same time, viral exposure leads to interferon production modulating bacterial communities and function (Huang and Landay, 1969; Li, 2007).

It is anticipated that analyses of the gut microbiota and core microbial communities of various chicken breeds could provide unique insights for novel strategies of pathogen exclusion, thus enhancing feed efficiency and disease resistance through microbiome modulation (Paul et al., 2021). The present study investigated cecal microbial communities of different chicken breeds in response to NDV. While NDV challenge and its effect on broiler gut microbiota has been explored in newly hatched and 21-day-old specific-pathogen-free chickens (Cui et al., 2018; Tong et al., 2022), the response may be impacted by native gut microflora, including various facultative pathogens that are acquired under normal rearing conditions. In addition, we provide novel insights for cecal microbiome alterations in response to ND among additional breeds—some, like Naked Neck, indigenous to Pakistan.

In the poultry industry, feed efficiency is the most important attribute because feed accounts for 70% of total production cost (Zampiga et al., 2018). In the present study, maximum feed efficiency was observed for broiler (Nolte et al., 2020). We observed an FCR of <2 for broilers, supported by Li et al. (2020), where average broiler FCR was 1.89. However, in our study, feed efficiency may be over/underestimated as feed wastage could not be calculated. Birds were infected at second week post-vaccination as the maximum antibody titers were reported during this time (Wang et al., 2015). NDV infection negatively affected the feed efficiency of broilers, possibly due to the decreased activity of digestive enzymes, resulting in poor production performance (Chansiripornchai & Sasipreeyajan, 2006; Rehman et al., 2021; Wang et al., 2015). We observed that broilers were the most susceptible to ND, as evidenced by higher disease severity than Naked Neck, Black Australorp, Rhode Island Red, and white layer. This is consistent with lower ND incidence in layer farms compared to broilers (Abbas et al., 2015). The disease onset and severity also varied between broiler and other breeds, which depends on viral strain as well as host susceptibility (Deist et al., 2017; Swayne et al., 2020). Considering the hematological parameters as a measure of resilience to infections, Naked Neck was revealed as the most resistant breed. It has previously been observed that autosomal Naked Neck genes such as Na and F have positive effects on immunocompetence in chickens (Adomako et al., 2014; Deeb and Cahaner, 1999; Fathi et al., 2014; Fernandes et al., 2023; Yakubu et al., 2008; Yalçin et al., 1997).

Like previous studies, we observed an increase in diversity as chicks age (Ameer et al., 2023; Crhanova et al., 2011; Danzeisen et al., 2011; Ijaz et al., 2018; Mohd Shaufi et al., 2015; Oakley et al., 2014a; Zou et al., 2022). In addition, high dissimilarity was observed between broiler cecal microbiomes and indigenous breeds, as observed in Chen et al. (2023) and Paul et al. (2021). Furthermore, we observed decreased diversity after NDV challenge, indicating that viral infection impacted the gut bacterial communities. NDV infection associated dysbiosis in chicken gut microflora was reported in Cui et al. (2018) and Li (2007), which led to inflammation and immune modulation (Chang and Lin, 2016). In contrast, Tong et al. (2022) reported no changes in microbiome diversity with NDV infection. However, the study assessed the birds after 21 days, which may be too short a time to assess shifts in the microbiome (Tong et al., 2022).

We observed the broiler core microbiome to be relatively unstable and influenced by the environment compared to other breeds, which have a relatively stable core. Firmicutes and Bacteroidota were the most abundant phyla in all breeds, with the highest prevalence in broiler. Both phyla are involved in the feed conversion efficiency of chickens through the digestion of non-starch polysaccharides and nitrogen cycling (Clavijo and Flórez, 2018; Oakley et al., 2014b; Roth et al., 2022; Sergeant et al., 2014). Firmicutes abundance is low in laying hens (Khan and Chousalkar, 2021; Roth et al., 2022), and similar trends were observed in white layers in this trial. Moreover, an increased abundance of Bacteroidetes in all breeds except broiler was observed, which has been associated with egg laying stages, where Bacteroidetes overtakes Firmicutes (Joat et al., 2021). Animals fed on high-fat and -fiber diets also show higher Firmicutes and Bacteroidetes abundance, respectively (De Wit et al., 2012; Hildebrandt et al., 2009). However, as all breeds were reared on a consistent diet, these changes in Firmicutes and Bacteroidetes abundance might be reflective of host-associated microbial selection and dispersal limitation. Interestingly, Campylobacterota—a major food safety concern (Pang et al., 2023) —was not detected in Naked Neck and white layer within the core microbiome. Many studies report a strong association of host genetics and environmental factors on the diversity, structure, composition, and stability of gut microbial communities (Benson et al., 2010; Khachatryan et al., 2008; Rawls et al., 2006; Vijay-Kumar et al., 2010; Wei et al., 2013; Wu et al., 2011). Two different chicken breeds raised under same conditions and fed the same diet have different gut microbiota compositions (Zhao et al., 2013). Furthermore, the moderate heritability of some bacterial families have been reported as an evidence of association between the host genome and gut microbiota, which could be due to the gut secretions and surface modification of epithelial cells (Meng et al., 2014).

We found differentially abundant genera upon NDV exposure, in response to either vaccination or challenge, compared to control within each breed. The majority of commensal bacteria showed decreased abundance after vaccination or challenge, indicating a dysbiotic state upon viral exposure. NDV infection has previously been reported as altering the chicken gut micro-ecosystem (Cui et al., 2018; Tong et al., 2022). Opportunistic pathogens such as *Campylobacter*, *Clostridium_sensu_stricto_1*, and *Escherichia–Shigella* were found to be upregulated in NDV infected groups. Similar findings were published by (Tong et al., 2022), in which a higher relative abundance of *Escherichia–Shigella* in NDV-challenged White Leghorn chickens was reported. The upregulation of opportunistic pathogens in response to ND is supported by evidence of CMP-pseudaminate biosynthesis upregulation, a pathway involved in promoting the motility, invasion, and immune evasion of certain pathogenic bacteria, including *Helicobacter* and *Campylobacter* (Chou et al., 2005; Josenhans et al., 2002; Schirm et al., 2003). In this study, these were among the genera which were significantly upregulated in response to NDV in broiler and Black Australorp. Moreover, bacterial pathogenicity may be enhanced by host interferon production against NDV, as it is known to enhance bacterial endotoxin lethality (Huang and Landay, 1969). Furthermore, breed-wise variation in relative abundance of different genera may suggest host selection pressure and associated immune responses (Paul et al., 2021).

In shotgun metagenomics data, *Tidjanibacter* was observed in infected broilers, which is associated with inflammatory conditions in humans (Parker et al., 2020). A higher abundance of anaerobic Clostridia were also observed, which is consistently reported in response to NDV infection. In fact, it has been reported that inflammation within the host may be beneficial to *Clostridioides difficile* as it exploits the degradation of collagen and other components of the extracellular matrix during inflammatory responses and uses them as source of protein. Moreover, *Clostridium* produces toxins that excludes *Bacteroides*, which competes for similar nutrients (Fletcher et al., 2021).

The present study revealed that microbial diversity, core microbiome membership, and differentially abundant bacterial genera harbored in the chicken gut of different breeds, which could reinforce disease prevention strategies, promote poultry gut health, and improve production performance. The data highlight the importance of rearing desi/local breeds due to their intrinsic disease resistance and stable core microbiome. Based on the study outcomes, a few recommendations could be communicated to poultry farmers and policymakers, including the implementation of strict biosecurity measures such as increased cleaning frequency and low stocking density, which could help reduce disease risks. Authorities should invest in future research with regular and extensive farm surveys. The study further illustrates the importance of metagenomic analysis along with amplicon data for an accurate representation of novel microbial communities. Through the application of metagenomics and metabolomics, the actual metabolic pathways in disease resistant breeds could be elaborated. This would offer a detailed understanding of how the microbiota contributes to immune competence against viral infections and could lead to important interventions to curtail disease spread and boost production performance.

## 5 Conclusion

This study identified breed-associated differences in cecal microbiome composition and NDV response, with local breeds showing indications of greater resilience to NDV than commercial broilers. These observations included higher survival, delayed onset of clinical signs, and more stable microbiome profiles following NDV challenge. While these trends suggest that certain local breeds may better withstand NDV infection, the implications for broader poultry sustainability should be interpreted cautiously, as multiple other diseases and production factors also influence overall system performance. Further research is needed to clarify the microbiome-linked mechanisms that underlie breed-specific resilience and to evaluate whether targeted microbiome modulation could improve disease tolerance in commercial breeds. Local breeds may hold potential value for NDV-endemic regions; however, these findings should be interpreted as exploratory rather than definitive, due to this study’s limited sample size and inherent variability in microbiome data. More comprehensive studies are required before drawing wider conclusions about their role in long-term poultry sustainability.

## Data Availability

The datasets presented in this study can be found in online repositories. The names of the repository/repositories and accession number(s) can be found at: http://dx.doi.org/10.1016/j.dib.2024.110957, data in brief https://www.ncbi.nlm.nih.gov/bioproject/?term&equals; PRJEB65106, PRJEB65106 https://doi.org/10.1016/j.dib.2024.110487, data in brief.
