# Supplementary material for: Avian cecal microbiome response and resilience to Newcastle disease are dictated by breed background
Source: Front Syst Biol. 2026 Feb 12;5:1659648. doi: 10.3389/fsysb.2025.1659648 (PMC12935917; doi:10.3389/fsysb.2025.1659648)
Supplement: Supplementary file 1 [file DataSheet1.pdf]

Supplementary Information

Avian caecal microbiome response and resilience to Newcastle disease is dictated by breed background

Aqsa Ameer[1], Farrukh Saleem[1,2], Ciara Keating[3], Farhan Afzal[4], Hamid Irshad[5], Khurshid Ahmed[2], Sadia Sattar[1], Umer Zeeshan Ijaz[6,7,8*], Sundus Javed[1*]

[1]Department of Biosciences, COMSATS University Islamabad, 44000, Pakistan

[2]National Veterinary Laboratories, Ministry of National Food Security and Research, Islamabad, 44000, Pakistan

[3]Department of Engineering, Durham University, Durham, DH1 3LE, UK

[4]Poultry Research Institute Rawalpindi, Livestock and Dairy Development Department Punjab Rawalpindi, 48061, Pakistan

[5]Animal Sciences Institute, National Agricultural Research Center, Islamabad, 44000, Pakistan

[6]Water & Environment Research Group, University of Glasgow, Mazumdar-Shaw Advanced Research Centre, Glasgow G11 6EW, UK

[7]Department of Molecular and Clinical Cancer Medicine, University of Liverpool, Liverpool L69 7BE, UK

[8]National University of Ireland, Galway, University Road, Galway, H91 TK33, Ireland

*** Joint corresponding authors** (both authors jointly directed this work)
umer.ijaz@glasgow.ac.uk and sundus.javed@comsats.edu.pk

Detailed Methods

Haemagglutination inhibition (HAI) assay

Briefly, 50µl of PBS was dispensed in each well of the micro titration plate. 50µl of serum sample was dispensed in the first well of the plate. Two-fold serial dilution of 50µl was made up to 10th well, leaving 11th and 12th well as positive and negative control. 50µl of the 4HA units (HAU) antigen was added up to 11th well, followed by 30 minutes incubation at room temperature. Volume of 50µl 1% of freshly prepared chicken RBCs were dispensed in all wells and left for 45 minutes at room temperature and observed the pattern.

Clinical Symptoms and disease severity scoring in challenged groups

Challenged birds were observed daily for mortality and morbidity up to 4 weeks post-challenge in case of layer breeds. Challenged broiler birds were observed for two weeks. Normal/healthy and sick/diseased birds were observed for clinical signs and symptoms such as body temperature, feeding/social behaviour, egg laying frequency and general physiology. Recovery time of the survivors were recorded. Birds were examined during slaughtering and performed post-mortem inspection covering thymic lobules, liver, ova/testicles, heart, kidney, lungs, spleen, intestine, gizzard, proventriculus and cecal tonsils. Birds were scored 0, 1, 2 and 3 for no clinical signs, mild clinical signs, severe clinical signs and death, respectively. Scoring time point was 7th day post challenge for indigenous breeds and 4th day post-challenge for broiler.

DNA Extraction and Quantification

Briefly, 0.1g of cecal sample and 700µL of lysis buffer (S1) was added in bead tube, and vortexed in order to disperse the sample thoroughly. Then to enhance the lysis, 100µL of lysis

enhancer (S2) was added followed by vortexing. Sample tubes were then homogenized in beat beater (Fastprep, MP Biomedical) for 2min in 2 steps by selecting the program of mouse tissue homogenization with 10min rest in between the cycles followed by centrifugation at 14000rcf for 5min. 400µL of the supernatant was transferred to a clean micro centrifuge tube and 250uL of clean-up buffer (S3) was added with immediate vortexing to ensure thorough S3 dispersion and precipitation of inhibitors, followed by centrifugation at 14000rcf for 2min. 500µL of supernatant was transferred into a clean centrifuge tube. To bind the DNA to the column, 900µL of binding buffer (S4) was added and vortexed. 700µL of this mixture was loaded onto the spin column (column tubes) and centrifuged for 1min at 14000rcf. Flow through was discarded and process repeated for remaining sample mixture. To ensure that the entire sample has passed into collection tube, centrifugation process was repeated at 14000rcf for 30sec to 1min. Spin column was then placed in a collection tube and 500µL of wash buffer (S5) was added to tube-column assembly and centrifuged at 14000rcf for 1min. Flow through was discarded and centrifugation repeated for 30sec at the same revolution, to ensure complete removal of S5. Finally, 100 µL of elution buffer (S6) was added and incubated at room temperature followed by centrifugation at 14000rcf for 1min. The purified DNA was collected in tube and an aliquot used for quality check and stored at 4°C. The remaining DNA was stored at -20°C until shipment for sequencing. A simple extraction tube was processed in parallel to serve as a negative control for sequencing to assess any kit associated contamination during processing.

Quantification of double stranded DNA (dsDNA) was performed on a Qubit Fluorometer using the high sensitivity dsDNA assay kit (Fisher Catalog #Q32856). First, a mastermix of Qubit™ working solution was prepared according to the number of samples and standards (2 standards i.e. S1 and S2 for calibration). Tube lids were labeled and aliquot Qubit™ working solution and sample was added to each tube to make up the final volume to 200µL. Tubes were incubated at room temperature for 2 minutes after vertexing. The sample Qubit™ 3 Fluorometer was used to quantify the fluorescence intensity of fluorescent dye binding to double-stranded DNA (dsDNA).

Bioinformatics

<u>16S rRNA</u>

Abundance tables were obtained by constructing OTUs table, a proxy for species level assignment using a modified workflow where the software choices for pre-processing MiSeq reads result in reducing the substitution error rates significantly (Schirmer et al. 2015). A total of 11,544,255 paired-end reads from n=111 samples (109 true samples and 2 negative controls) were trimmed and filtered using Sickle (Joshi 2011) by applying a sliding window approach and trimming regions where the average base quality drops below 20, and retaining resultants paired-end reads with at least 50bp length. These reduced the total number of reads to 11,400,805. BayesHammer (Nikolenko et al. 2013) was then used to error correct the paired-end reads. The paired-end reads were overlapped using PandaSeq (Masella et al. 2012) using a simple Bayesian approach and with a minimum overlap of 10bp and that resulted in a total of 10,749,141 reads. After obtaining the consensus sequences from each sample, VSEARCH pipeline (Rognes et al. 2016) was used (all these steps are documented in https://github.com/torognes/vsearch/wiki/VSEARCH-pipeline) for OTU construction. The approach is as follows: reads from different samples were pooled together and barcodes added to keep an account of the samples these reads originate from. Reads were then dereplicated and sorted by decreasing abundance and singletons discarded. In the next step, the reads are clustered based on 99% similarity, followed by removing clusters that have chimeric models built from more abundant reads (–uchime_denovo option in vsearch). A few chimeras may be missed, especially if they have parents that are absent from the reads or are present with very low abundance. Therefore, in the next step, a reference-based chimera filtering step (–uchime_ref option in vsearch) was applied using a gold database (https://www.mothur.org/w/images/f/f1/Silva.gold.bacteria.zip). The original barcoded reads were matched against clean OTUs with 99% similarity (a proxy for species level separation) to generate OTU table (a total of 74,049 unique sequences) for 111 samples.

SILVA SSU Ref NR database release v.138 (Quast et al. 2013) was then used to assign taxonomy, and generated the rooted phylogenetic tree (using qiime phylogeny align-to-tree-mafft-fasttree) within the QIIME2 framework (Bolyen et al. 2019). Furthermore, PICRUSt2 (Douglas et al. 2020) within the QIIME environment was used to recover predicted KEGG enzymes (10,543 enzymes for 111 samples) and MetaCyc pathway (487 enzymes for 111 samples) for all the samples. For this purpose, parameters --p-hsp-method pic --p-max-nsti 2 in qiime picrust2 full-pipeline [https://github.com/gavinmdouglas/q2-picrust2] were used. Of 74,049 unique sequences, 73,453 were searched in PICRUSt2, with only 127 sequences, failing the NSTI criteria of 2 which is a quantitative criteria to match sequences in the reference database, thus increasing the reliability and confidence in the metabolic predictions. QIIME2 was also used to generate a final BIOM file that combined abundance information with the new taxonomy (summary statistics of OTUs/sample as follows: [Min: 8,423; 1st Quartile: 58,892; Median: 70,719; Mean: 69,671; 3rd Quartile: 87,850; Max: 121,554]), and which along with the recent phylogenetic assignment, and the meta-data was used for the downstream statistical analysis.

Shotgun metagenomics

Shotgun sequencing was performed on Illumina TruSeq ensuring ~20M reads per samples for 8 broiler samples (healthy control n=4 and NDV infected n=4) using 2x100bp reads at Glasgow Polyomics sequencing facility. Adapter trimmed reads were provided by the sequencing facility. These reads were then further trimmed using Sickle v1.200 (Joshi 2011) where the average Phred quality dropped below 20 and retained paired-end reads of length ≥50bp. This has given a total of 163,357,856 reads from all samples. Then all the forward and reverse reads were collated together, and co-assembly was done using megahit with the parameters --k-list 27,47,67,87 --kmin-1pass -m 0.95 --min-contig-len 1000 (Li et al. 2015). This resulted in a total of 202,665 contigs, 1,396,298,223 base pairs (bp), maximum of 344,753 bp, average length of 6,890 bp and an N50 score of 16,582 bp. MetaWRAP pipeline (Uritskiy et al. 2018) was then used and to bin the contigs using three different binning algorithms: metabat2 (198 bins) (Kang et al. 2019), maxbin2 (167 bins) (Wu et al. 2016), and CONCOCT (253 bins) (Alneberg et al. 2014). On these bins, CheckM (Parks et al. 2015) was applied to assess their completion as well as contamination. Within MetaWRAP framework, the bins from the three binners were consolidated together only retaining bins with ≥50% completion, and ≤10% contamination to give a final set of 130 bins or metagenomic assembled genomes (MAGs). For the bins, a mean genome completion of 82.90% and a mean contamination of 1.79% was obtained.

To obtain metabolic functions, nutrient cycling diagrams (Carbon, Sulfur, including taxonomy using GTDB-TK (Chaumeil et al. 2020), METABOLIC pipeline (Zhou et al. 2022) was used. METABOLIC allowed for the recovery of annotated proteins using KEGG (Kanehisa and Goto 2000), TIGRfam (Selengut et al. 2007), Pfam (Finn et al. 2014), custom hidden Markov model (HMM) databases (Anantharaman et al. 2016), dbCAN2 (Zhang et al. 2018), and MEROPS (Rawlings et al. 2016).

To infer MAGs phylogeny, GToTree (Lee 2019) was used. The software provides several Single Copy Genes (SCGs) sets depending on the resolution of the domains and taxonomic rank of interest. Two SCG sets were used, a 25-gene Bacteria and Archaea SCG set (recovered phylogeny for 81 MAGs) and a 16 genes SCG set (recovered phylogeny for 70 MAGs) by (Hug et al. 2016) that covers all major domains of life. To see which MAGs are novel, Genome Tree Toolkit (https://github.com/donovan-h-parks/GenomeTreeTk) was used by checking the phylogenetic gain for each MAG against the rest of the tree, with higher values potentially identifying novel species. These were calculated for each MAG in the trees recovered for both the 25 genes Bacteria and Archaea SCGs, and the 16 genes SCGs from (Hug et al. 2016), respectively.

Supplementary Table 1: Disease severity scores post NDV challenge

Chicken number	Naked Neck		Black Australorp		Rhode Island Red		White Layer		Broiler	
	Vaccinated	Non-vaccinated	Vaccinated	Non-vaccinated	Vaccinated	Non-vaccinated	Vaccinated	Non-vaccinated	Vaccinated	Non-vaccinated
1	1	3	1	2	2	1	1	1	1	3
2	1	1	1	2	1	1	1	1	0	3
3	1	1	1	1	1	0	1	1	0	3
4	0	1	0	1	0	0	0	1	0	3
5	0	0	0	0	0	0	0	0	0	2
Average Score	0.6	1.2	0.6	1.2	0.8	0.4	0.6	0.8	0.2	2.8

**No clinical sign= 0, mild clinical signs=1, severe clinical signs=2, death=3

**Time point of scoring for Layer breeds was 7th day post-challenge; for Broiler 4th day post-challenge

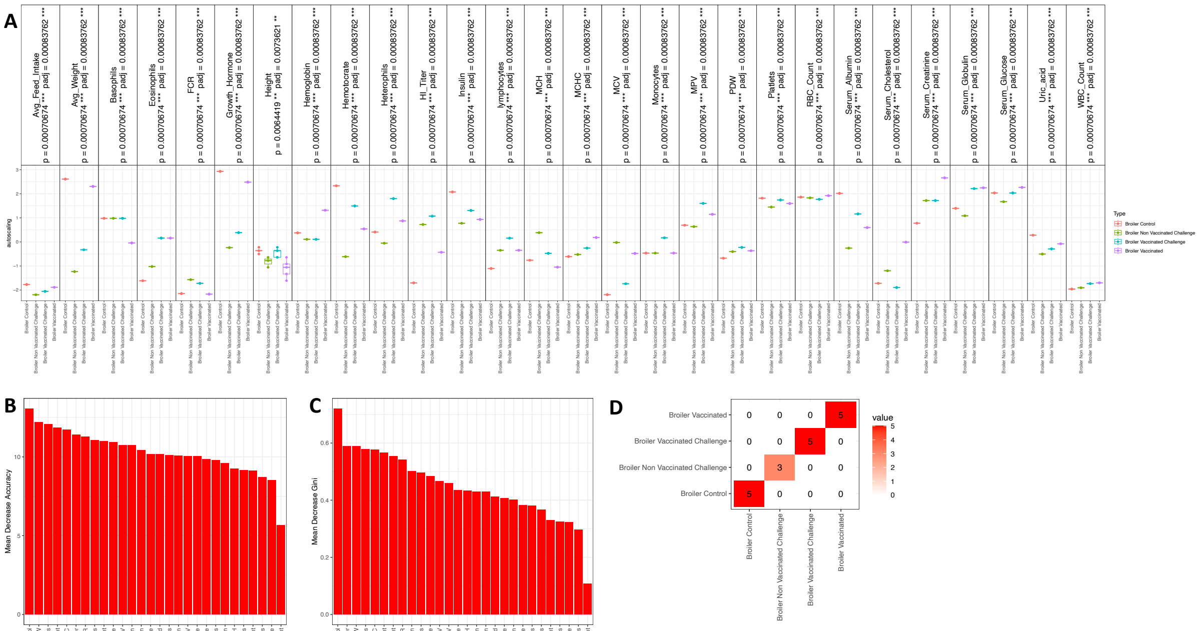

Supplementary Figure S1. Comparison of key parameters for different treatment groups for Broiler. After autoscaling the data, the non-parametric Kruskal Wallis test was performed with p-values adjusted for multiple comparisons using Benjamini & Hochberg (1995). The final list of parameters with adjusted p-values < 0.05 are shown in (A). Afterwards, a Random Forest Classifier is fitted on these parameters. Two importance measures, (B) Mean Decrease in Accuracy, and (C) Mean Decrease Gini ranks the features in terms of decreasing importance, i.e., the most important features that segregate between different conditions are shown on the left. To see the performance of the classifier the confusion matrix is shown in (D) where the rows represent the original labels and the columns represent the predicted labels by the classifier.

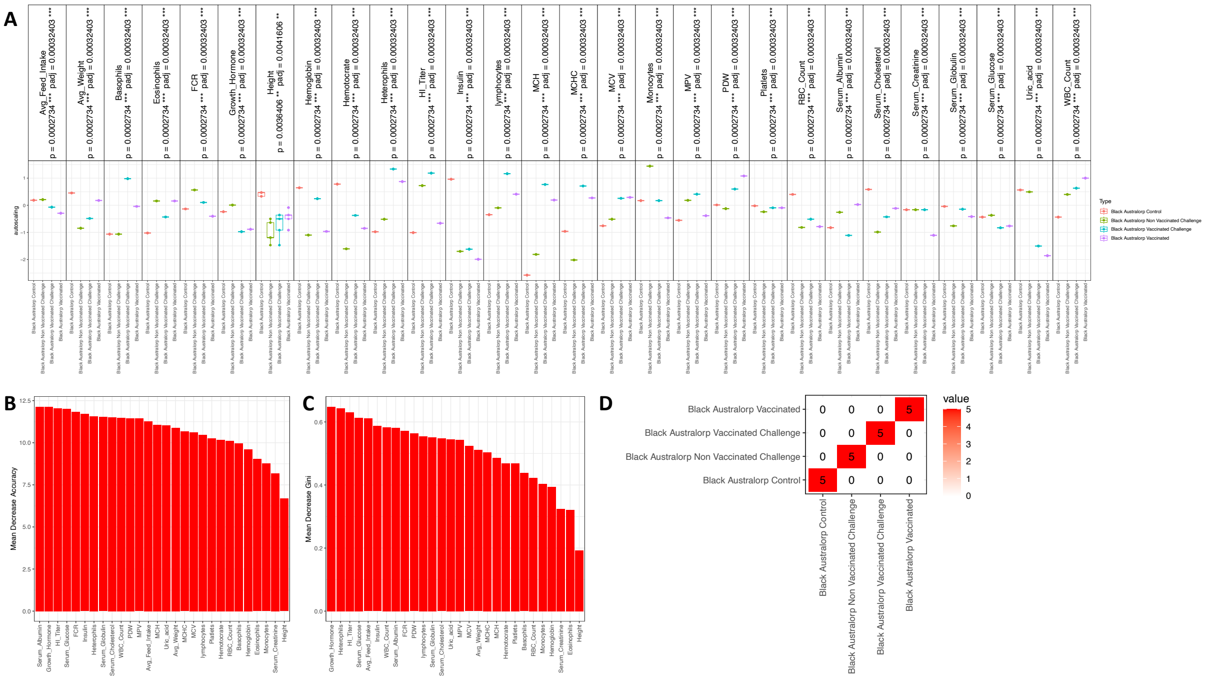

Supplementary Figure S2. Comparison of key parameters for different treatment groups for Black Australorp. The description is similar to the one provided in Supplementary Figure S1.

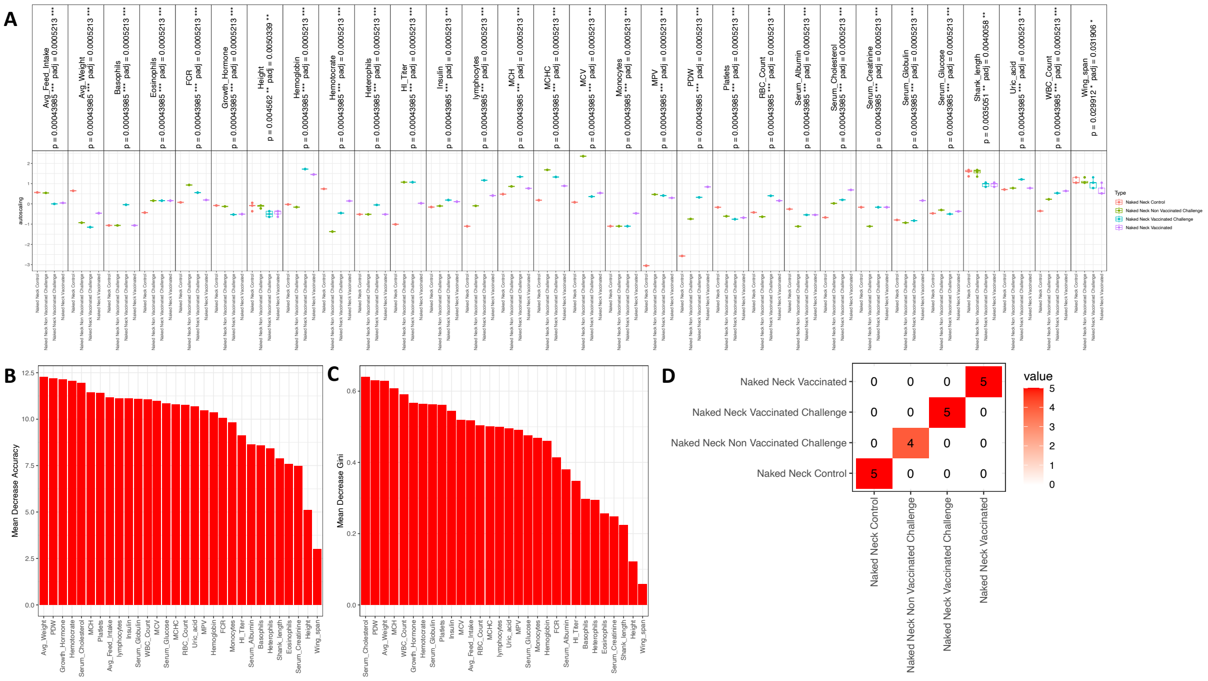

Supplementary Figure S3. Comparison of key parameters for different treatment groups for Naked Neck. The description is similar to the one provided in Supplementary Figure S1.

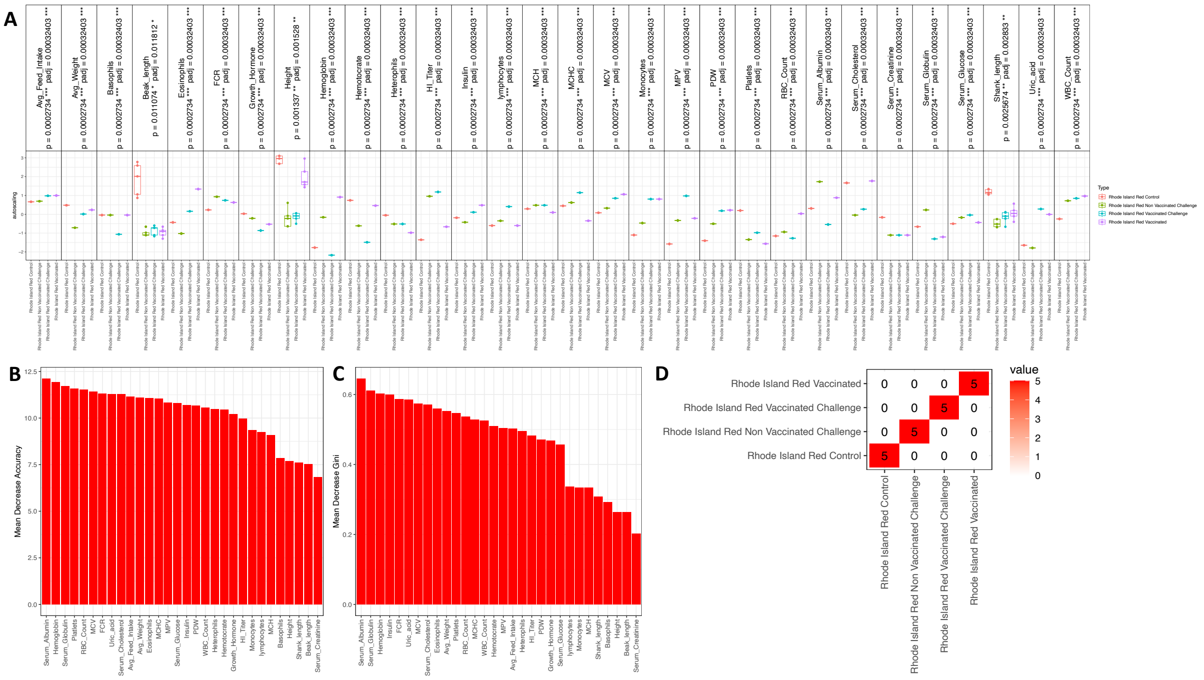

Supplementary Figure S4. Comparison of key parameters for different treatment groups for Rhode Island Red. The description is similar to the one provided in Supplementary Figure S1.

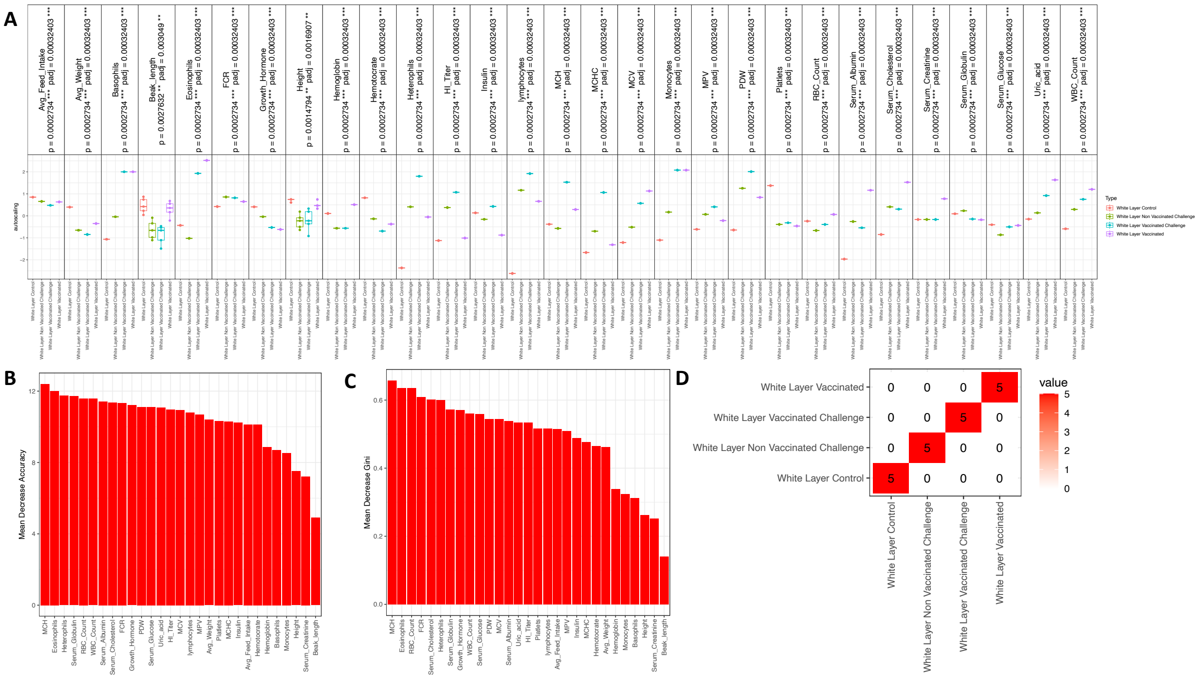

Supplementary Figure S5. Comparison of key parameters for different treatment groups for White Layer. The description is similar to the one provided in Supplementary Figure S1.

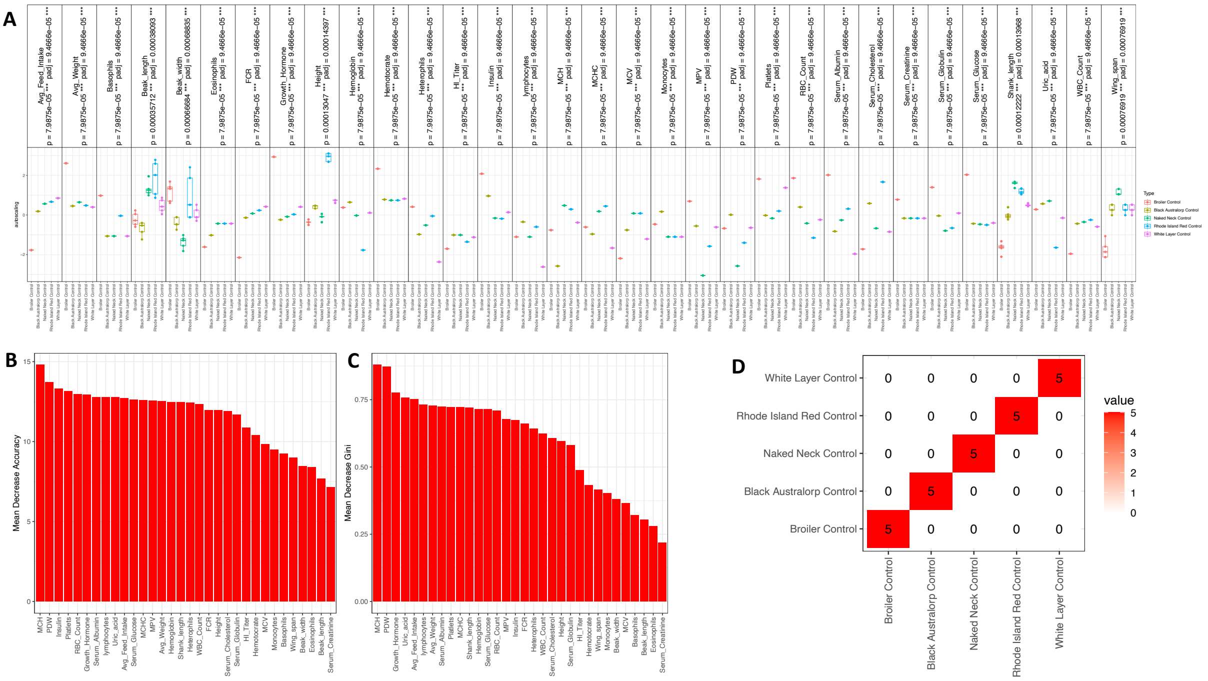

Supplementary Figure S6. Comparison of key parameters for all Control groups. The description is similar to the one provided in Supplementary Figure S1.

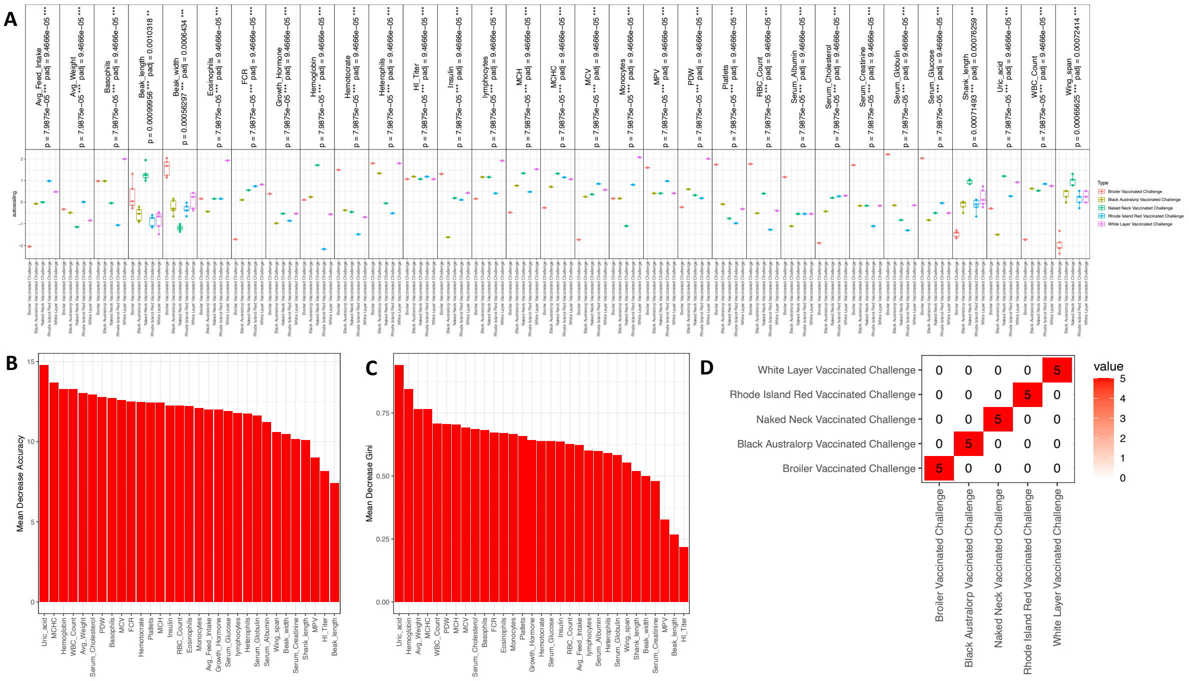

Supplementary Figure S7. Comparison of key parameters for all Vaccinated Challenge groups. The description is similar to the one provided in Supplementary Figure S1.

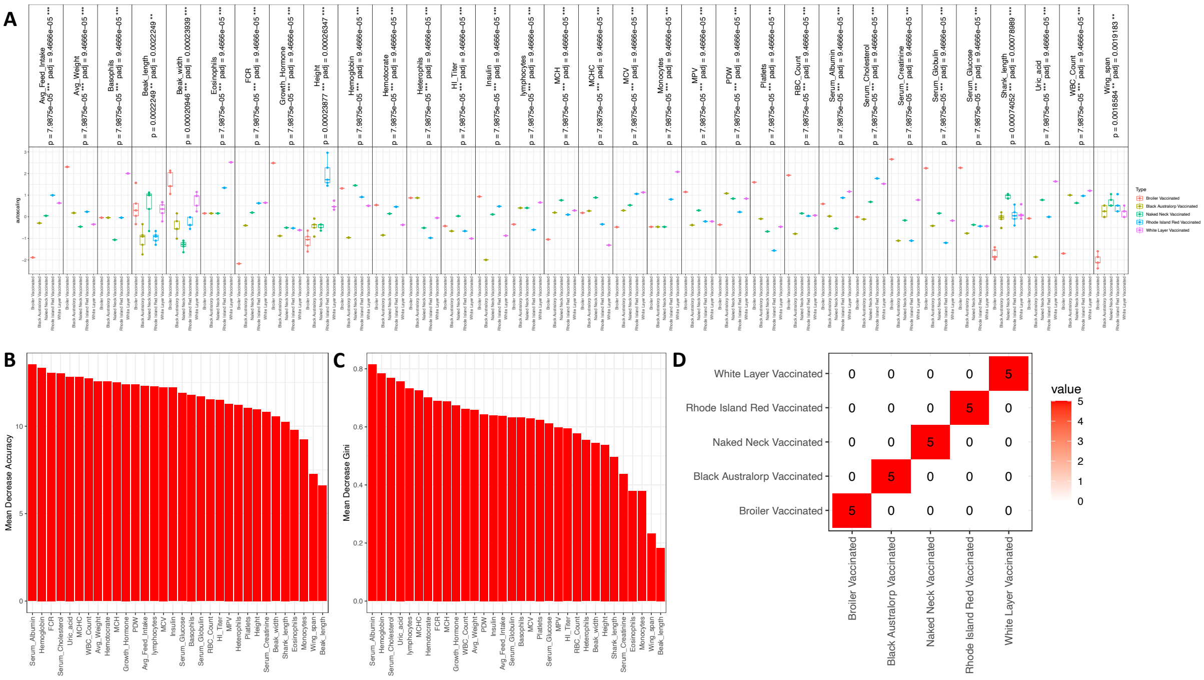

Supplementary Figure S8. Comparison of key parameters for all Vaccinated groups. The description is similar to the one provided in Supplementary Figure S1.

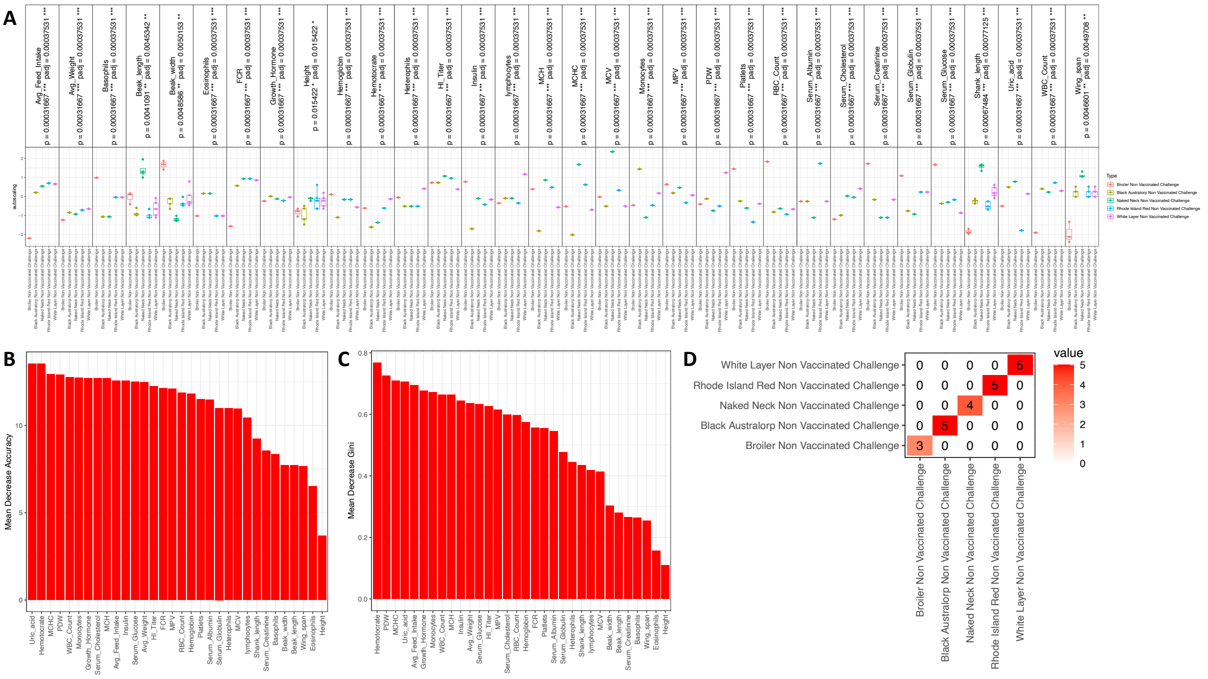

Supplementary Figure S9. Comparison of key parameters for all Non Vaccinated groups. The description is similar to the one provided in Supplementary Figure S1.

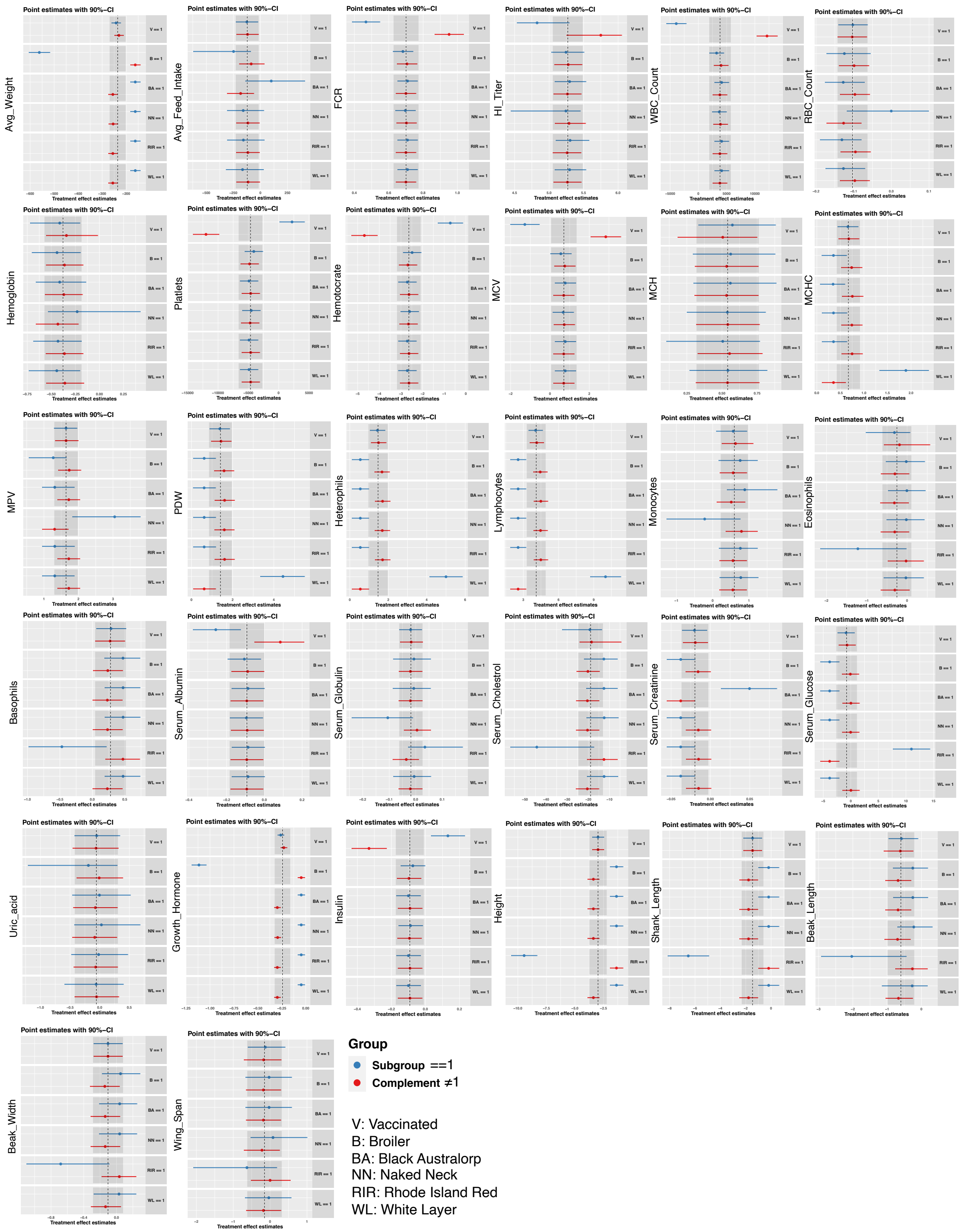

Supplementary Figure 10: Subgroup treatment effect estimation of parameters. For each parameter, the forest plot shows the difference of that particular parameter between the challenged and non-challenged birds as a point estimate with 90% confidence interval. This is shown for all possible subgroups (represented by blue line) and their complements (represented by red line). The vertical dashed line as well as its confidence intervals in the grey shaded area represent the region in which there is no effect of treatment (i.e., no difference between the challenged and non-challenged) and subgroup (i.e., no difference between the subgroup and its compliment).

Supplementary Table S3: Fitting LASSO regression to covariates. Covariates where β coefficients were zeroes across all models or not used in any of the models (NA: since the value didn't change) are not shown. These are β_{PDW}, $\beta_{\text{Heterophils}}$, $\beta_{\text{Monocytes}}$, $\beta_{\text{Eosinophils}}$, $\beta_{\text{Basophils}}$, $\beta_{\text{Serum\_Globulin}}$, $\beta_{\text{Serum\_Creatinine}}$, $\beta_{\text{Serum\_Glucose}}$, $\beta_{\text{C\_Reactive\_Protein\_pa}}$, β_{Insulin}, β_{Height}, $\beta_{\text{Shank\_length}}$, $\beta_{\text{Beak\_length}}$, $\beta_{\text{Beak\_width}}$, and $\beta_{\text{Wing\_span}}$. The colours represent which category the covariates were upregulated in whether Control (Yellow) or treatment (Green).

		Effect of NDV Vaccination					Effect of NDV Infection					Effect of NDV Vaccination + Infection				
Output Y	$P(Y = 0\| x)$	Broiler Control	White Layer Control	Naked Neck Control	Rhode Island Red Control	Black Australorp Control	Broiler Control	White Layer Control	Naked Neck Control	Rhode Island Red Control	Black Australorp Control	Broiler Control	White Layer Control	Naked Neck Control	Rhode Island Red Control	Black Australorp Control
	$P(Y = 1\| x)$	Broiler Vaccinated	White Layer Vaccinated	Naked Neck Vaccinated	Rhode Island Red Vaccinated	Black Australorp Vaccinated	Broiler Non Vaccinated Challenge	White Layer Non Vaccinated Challenge	Naked Neck Non Vaccinated Challenge	Rhode Island Red Non Vaccinated Challenge	Black Australorp Non Vaccinated Challenge	Broiler Vaccinated Challenge	White Layer Vaccinated Challenge	Naked Neck Vaccinated Challenge	Rhode Island Red Vaccinated Challenge	Black Australorp Vaccinated Challenge
β_0(Intercept)		369.0877091	115.8645612	78.87024188	361.709163	323.9502552	24.2255554	80.79254732	53.71976048	71.16554264	64.75851647	33.48438965	67.40141476	46.49964699	189.0021193	91.54303637

β_{WBC_Count}	β_{HI_Titer}	β_{FCR}	$\beta_{Avg\_Feed\_Intake}$	β_{Avg_Weight}
0	0	-7.04E-14	-6.64E-18	-0.192483812
0	0	1.05E-15	-2.67E-18	-0.078419331
0	0	6.15E-15	0	-0.052933048
0	1.74E-16	6.03E-16	1.16E-18	-0.235257992
0	0	-2.67E-15	0	-0.211732193
0	0	2.41E-15	-5.73E-18	-0.015325336
0	4.83E-16	0	0	-0.055718998
0	5.78E-17	0	-1.14E-15	-0.037189562
1.99E-20	0	0	0	-0.049012082
0	6.97E-17	3.41E-16	0	-0.045049403
0	4.36E-17	5.62E-16	-7.40E-18	-0.019937118
1.43E-20	0	2.41E-15	-3.63E-18	-0.047051598
0	5.81E-17	0	0	-0.032574184
5.28E-20	0	0	0	-0.124548349
3.61E-20	1.10E-16	9.98E-16	-3.77E-18	-0.062274174

β_{MCH}	β_{MCV}	$\beta_{Hemotocrate}$	$\beta_{Platlets}$	$\beta_{Hemoglobin}$	β_{RBC_Count}
-4.65E-15	0	0	0	0	0
0	0	0	0	0	0
0	0	0	0	0	1.84E-15
0	0	0	0	0	0
0	0	0	0	0	0
0	0	0	-6.78E-20	0	-9.73E-12
-3.49E-15	0	0	0	0	0
0	0	0	0	0	0
0	0	0	0	0	2.49E-14
0	4.98E-16	-5.81E-17	0	0	-8.72E-16
0	5.36E-16	0	-3.49E-19	-1.74E-15	0
3.49E-16	2.05E-16	0	0	0	0
7.75E-16	0	-2.32E-16	0	0	0
0	0	0	0	0	-1.74E-14
0	0	0	0	-4.65E-15	0

$\beta_{Serum\_Cholesterol}$	$\beta_{Serum\_Albumin}$	$\beta_{lymphocytes}$	β_{MPV}	β_{MCHC}
0	0	0	0	0
0	0	0	0	0
0	0	0	0	0
0	1.74E-15	NA	0	-1.16E-15
0	0	0	0	0
0	0	0	0	0
0	0	0	0	0
0	0	0	0	0
-7.12E-18	0	0	0	0
0	0	0	0	-5.81E-16
0	0	0	2.18E-16	0
0	0	0	1.94E-16	0
0	0	0	0	0
0	0	0	0	0
0	-3.49E-15	5.81E-17	0	0

$\beta_{Growth\_Hormone}$	β_{Uric_acid}
0	0
0	0
0	0
0	0
0	-1.03E-16
0	0
0	0
0	0
0	0
3.17E-15	0
0	0
0	0
0	0
0	0
0	0

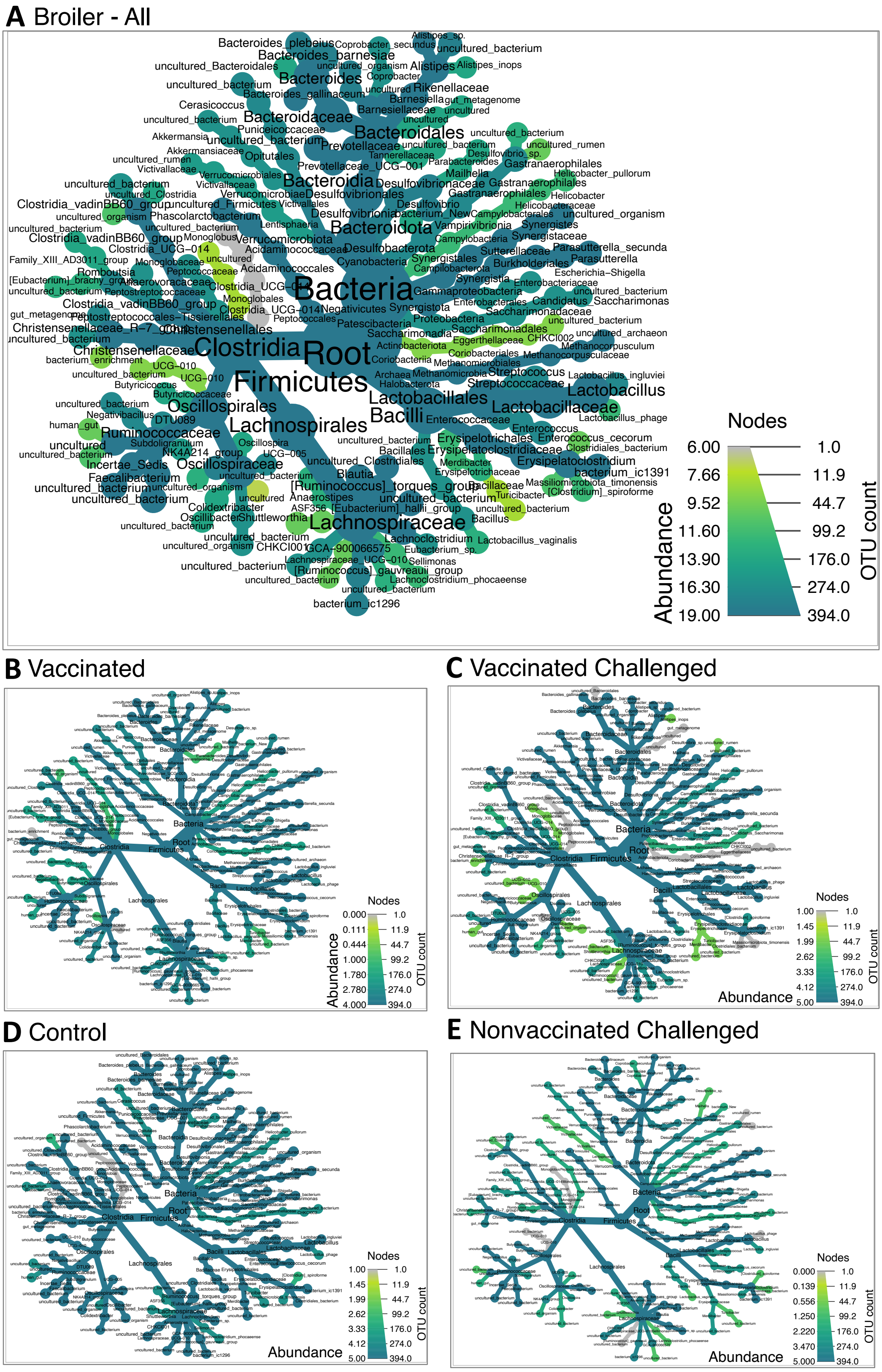

Supplementary Figure S11. Taxonomic tree of core OTUs recovered for Broiler with (A) representing collated abundances (all groups merged together) whilst (B), (C), (D), and (E) are treatment group specific abundances.

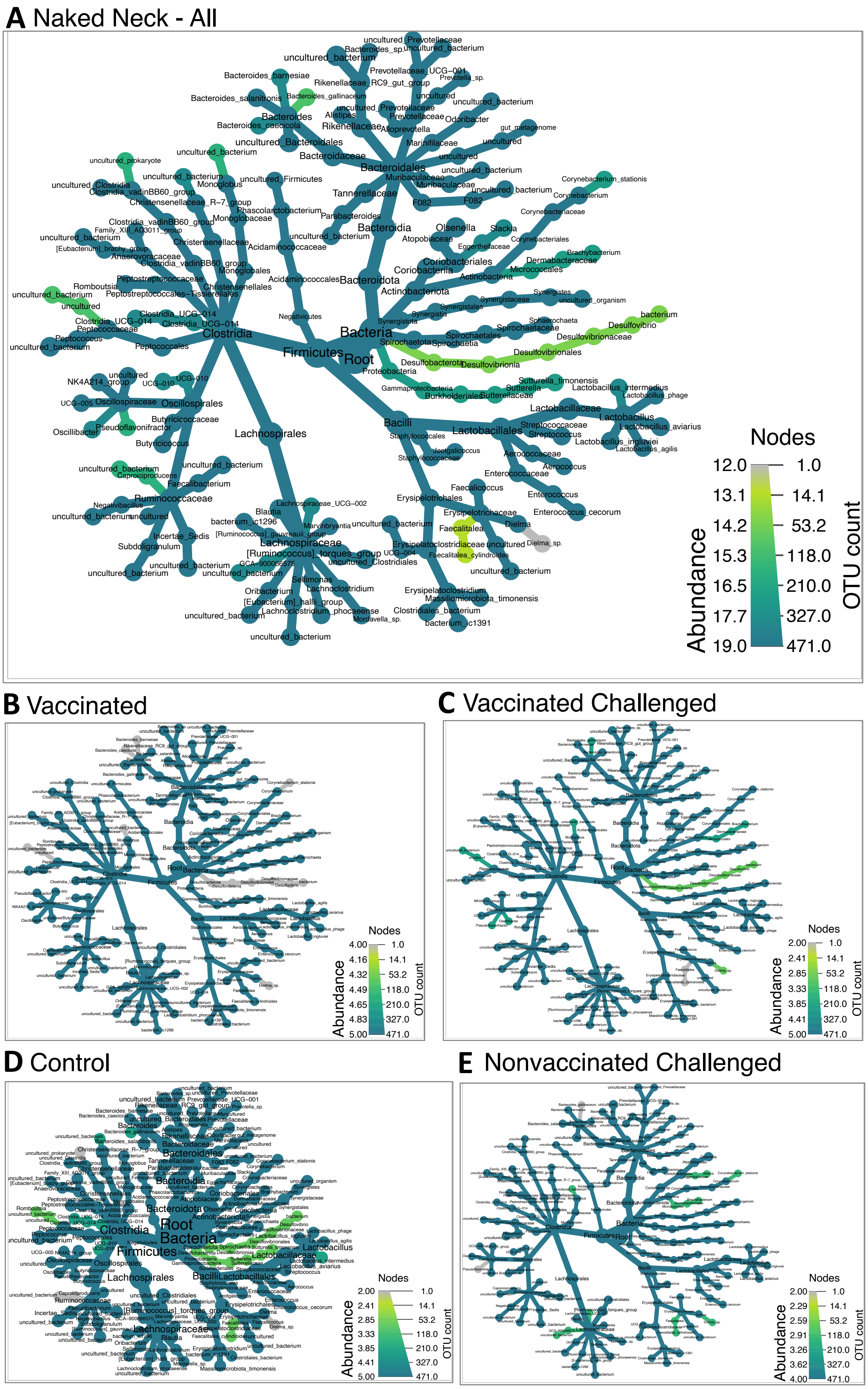

Supplementary Figure S12. Taxonomic tree of core OTUs recovered for Naked Neck with (A) representing collated abundances (all groups merged together) whilst (B), (C), (D), and (E) are treatment group specific abundances.

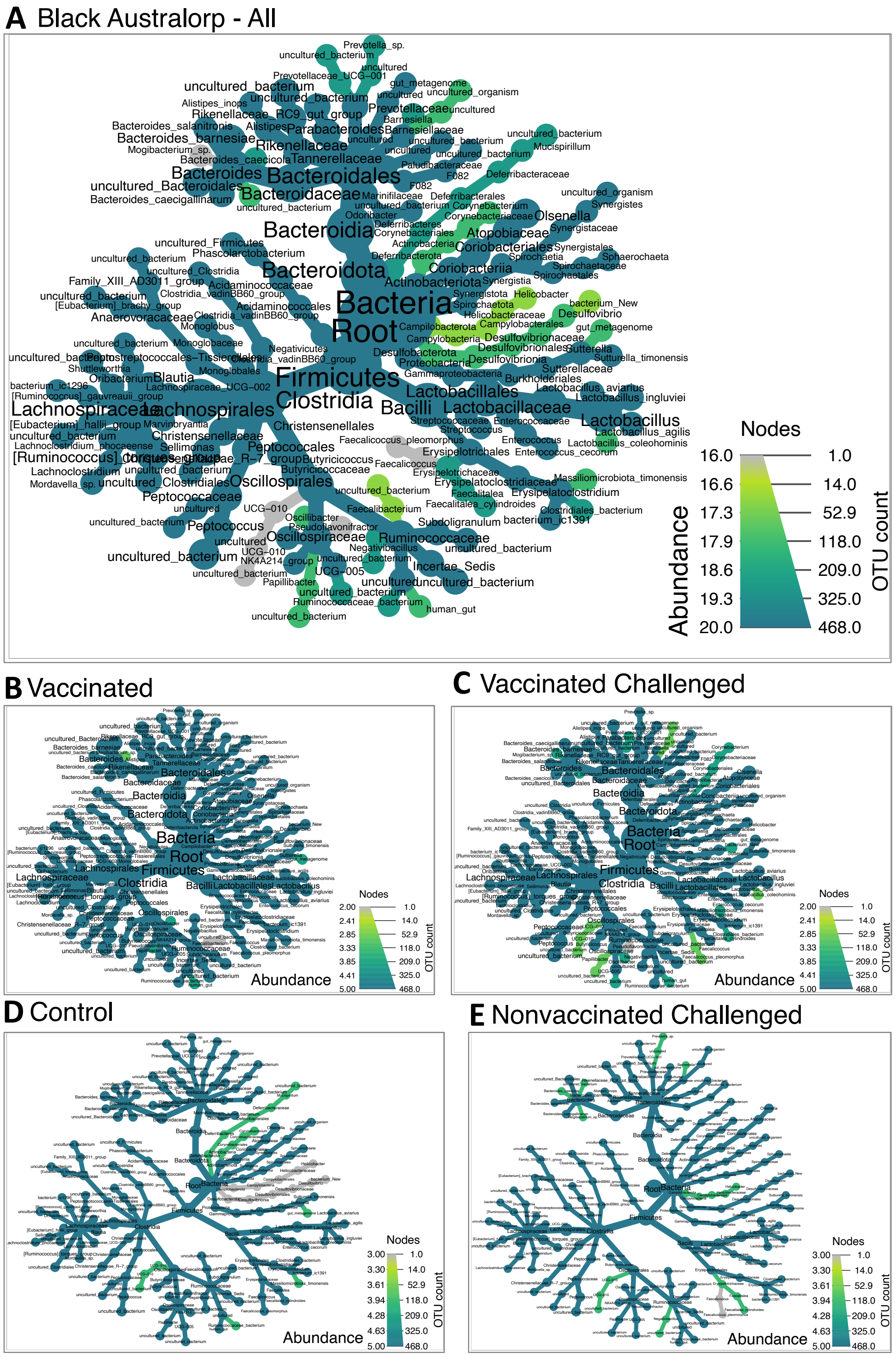

Supplementary Figure S13. Taxonomic tree of core OTUs recovered for Black Australorp with (A) representing collated abundances (all groups merged together) whilst (B), (C), (D), and (E) are treatment group specific abundances.

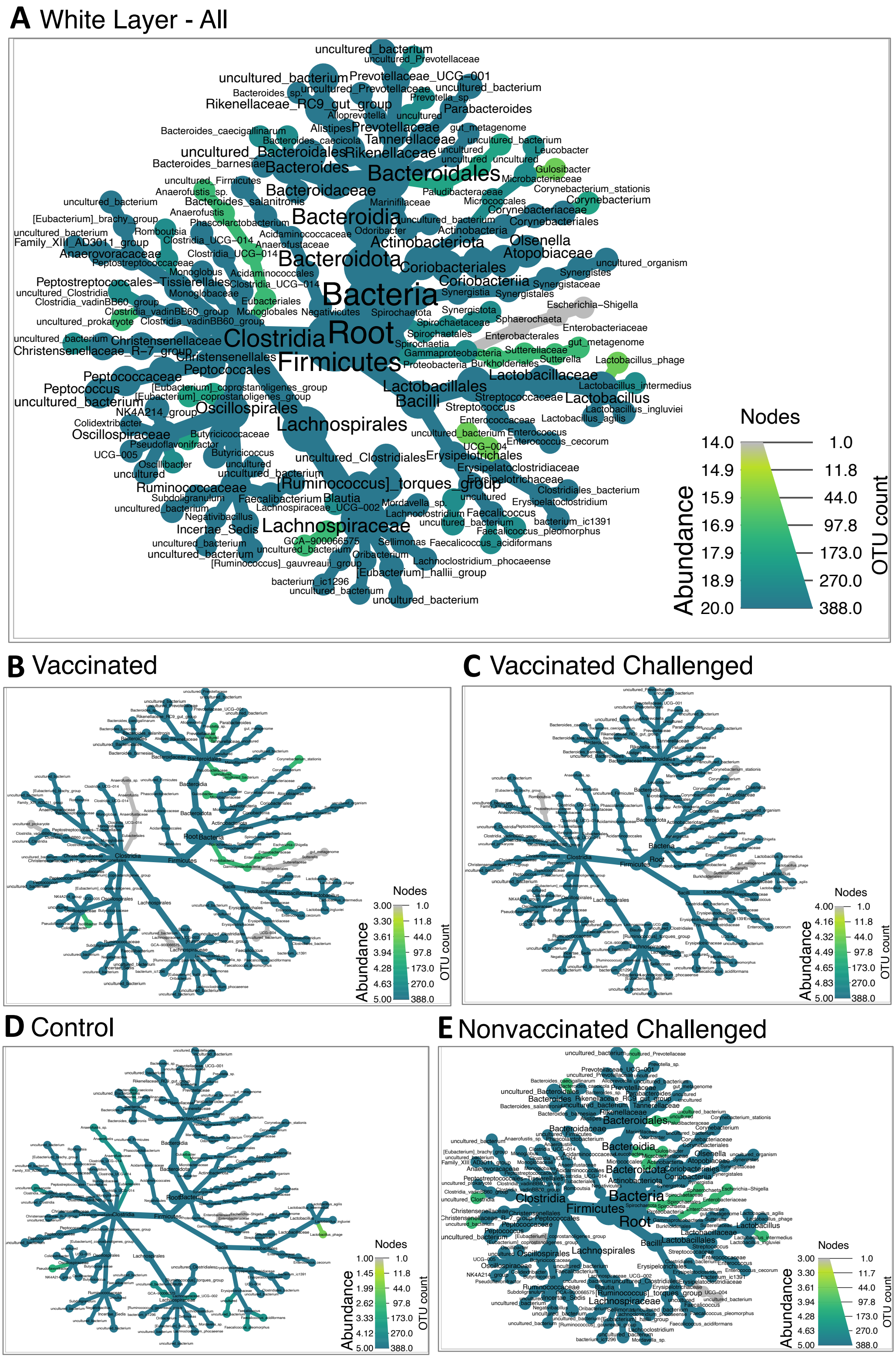

Supplementary Figure S14. Taxonomic tree of core OTUs recovered for White Layer with (A) representing collated abundances (all groups merged together) whilst (B), (C), (D), and (E) are treatment group specific abundances.

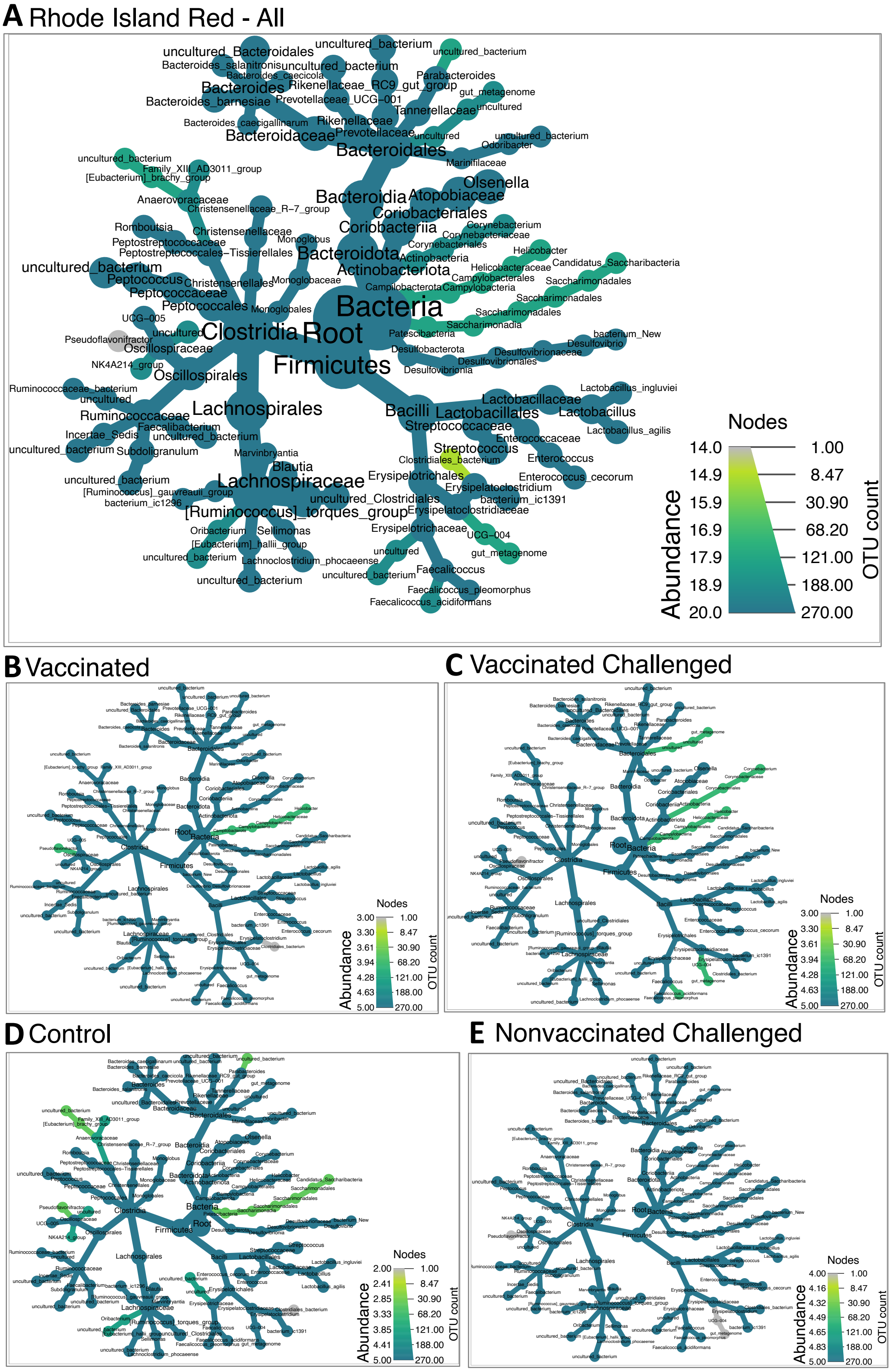

Supplementary Figure S15. Taxonomic tree of core OTUs recovered for Rhode Island Red with (A) representing collated abundances (all groups merged together) whilst (B), (C), (D), and (E) are treatment group specific abundances.

Supplementary Table S4: Redundancy analysis with forward selection was performed to select the most important environmental variables that explain variation in the community matrices. The initial set of variables considered are as follows (with those selected in the final PERMANOVA models in bold case): **Average Weight**, **Treatment** (*Control*, *Non Vaccinated Challenge*, *Vaccinated Challenge*, *Vaccinated*), **Breed** (*Broiler, Black Australorp, Naked Neck, Rhode Island Red, White Layer*), **Average Feed Intake, Feed Conversion Ratio (FCR)**, **Hemagglutination Inhibition (HI) Titer**, **White Blood Cells (WBC) Count**, **Red Blood Cells (RBC) Count**, **Hemoglobin**, **Platlets**, **Hemotocrate**, **Mean Corpuscular Volume (MCV)**, **Mean Platelet Volume (MCH)**, Mean Corpuscular Hemoglobin Concentration (MCHC), **Mean Platelet Volume (MPV)**, **Platelet Distribution Width (PDW)**, **Heterophils**, **Lymphocytes**, **Monocytes**, **Eosinophils**, **Basophils**, **Serum Albumin**, Serum Globulin, Serum Cholesterol, **Serum Creatinine**, **Serum Glucose**, **Uric Acid**, C Reactive Protein, **Growth Hormone**, **Insulin**, Height of bird, **Shank Length of Bird**, **Beak Length of Bird**, **Beak Width of bird**, Wing Span of Bird. Here df, SS, and F are Degree of Freedom, Sum of Squared Errors, and F Statistic, respectively.

	Covariates	Df	SS	R^2	F	P
Bray-Curtis Distance						
	**Breed**	**4**	**6.2004**	**0.27992**	**11.1715**	**0.001 *****
	**Growth Hormone**	**1**	**0.8725**	**0.03939**	**6.2879**	**0.001 *****
	**Treatment**	**3**	**1.3725**	**0.06196**	**3.2971**	**0.001 *****
	**WBC Count**	**1**	**0.4231**	**0.0191**	**3.0493**	**0.001 *****
	**FCR**	**1**	**0.3926**	**0.01773**	**2.8296**	**0.001 *****
	**Heterophils**	**1**	**0.3499**	**0.01579**	**2.5214**	**0.005 ****
	**PDW**	**1**	**0.341**	**0.0154**	**2.4579**	**0.003 ****
	**MCH**	**1**	**0.2698**	**0.01218**	**1.9448**	**0.023 ***
	**MCV**	**1**	**0.2522**	**0.01139**	**1.8179**	**0.027 ***
	**Platlets**	**1**	**0.2335**	**0.01054**	**1.6828**	**0.038 ***
	**Insulin**	**1**	**0.2344**	**0.01058**	**1.6891**	**0.037 ***
	**HI Titer**	**1**	**0.2316**	**0.01045**	**1.669**	**0.056 .**
	**Serum Glucose**	**1**	**0.2017**	**0.00911**	**1.4536**	**0.095 .**
	**Eosinophils**	**1**	**0.1908**	**0.00861**	**1.375**	**0.1 .**
	Beak Length of Bird	1	0.1832	0.00827	1.3205	0.13

	Beak Width of Bird	1	0.1336	0.00603	0.9632	0.44
	Residual	74	10.2678	0.46354		
	Total	95	22.1507	1		
Unweighted Uni-Frac						
	**Breed**	**4**	**4.3483**	**0.23834**	**7.9555**	**0.001 *** **
	**Growth Hormone**	**1**	**0.4789**	**0.02625**	**3.5049**	**0.001 *** **
	**WBC Count**	**1**	**0.329**	**0.01803**	**2.4079**	**0.005 ** **
	**HI Titer**	**1**	**0.296**	**0.01623**	**2.1664**	**0.005 ** **
	**RBC Count**	**1**	**0.2279**	**0.01249**	**1.6676**	**0.035 * **
	**Serum Creatinine**	**1**	**0.2173**	**0.01191**	**1.5902**	**0.037 * **
	**Average Weight**	**1**	**0.2156**	**0.01182**	**1.5777**	**0.044 * **
	**Eosinophils**	**1**	**0.1985**	**0.01088**	**1.4526**	**0.062 .**
	**PDW**	**1**	**0.2003**	**0.01098**	**1.4658**	**0.076 .**
	**Hemotocrate**	**1**	**0.1923**	**0.01054**	**1.4075**	**0.081 .**
	**Average Feed Intake**	**1**	**0.1839**	**0.01008**	**1.3461**	**0.089 .**
	MCV	1	0.1792	0.00982	1.3115	0.101
	**MCH**	**1**	**0.2032**	**0.01114**	**1.4871**	**0.063 .**
	Basophils	1	0.17	0.00932	1.2443	0.142
	**Serum Albumin**	**1**	**0.206**	**0.01129**	**1.5073**	**0.06 .**
	MPV	1	0.171	0.00937	1.2512	0.12
	Beak Width of Bird	1	0.1605	0.0088	1.1748	0.152
	Shank Length of Bird	1	0.1543	0.00846	1.1292	0.236
	Residual	74	10.1117	0.55425		
	Total	95	18.244	1		

Weighted UniFrac						
	**Breed**	**4**	**0.19498**	**0.31001**	**13.8443**	**0.001 *** **
	**Treatment**	**3**	**0.0507**	**0.08061**	**4.7996**	**0.001 *** **
	**Growth Hormone**	**1**	**0.02668**	**0.04242**	**7.5779**	**0.001 *** **
	**FCR**	**1**	**0.02513**	**0.03996**	**7.1388**	**0.001 *** **
	**Eosinophils**	**1**	**0.01086**	**0.01726**	**3.0833**	**0.007 ** **
	**WBC Count**	**1**	**0.01136**	**0.01806**	**3.2264**	**0.007 ** **
	**MCH**	**1**	**0.00909**	**0.01445**	**2.5807**	**0.014 * **
	**Monocytes**	**1**	**0.00813**	**0.01293**	**2.31**	**0.031 * **
	**Uric Acid**	**1**	**0.00682**	**0.01085**	**1.9373**	**0.068 .**
	Residual	81	0.28519	0.45345		
	Total	95	0.62893	1		
Hierarchical Meta-Storms						
	**Breed**	**4**	**0.028633**	**0.23821**	**9.4264**	**0.001 *** **
	**Treatment**	**3**	**0.011887**	**0.09889**	**5.2179**	**0.001 *** **
	**RBC Count**	**1**	**0.005881**	**0.04893**	**7.7447**	**0.001 *** **
	**Lymphocytes**	**1**	**0.003064**	**0.02549**	**4.0347**	**0.01 ** **
	**WBC Count**	**1**	**0.001877**	**0.01561**	**2.4713**	**0.044 * **
	Serum Albumin	1	0.00087	0.00724	1.1453	0.303
	Heterophils	1	0.00148	0.01231	1.9489	0.109
	**Average Weight**	**1**	**0.00149**	**0.0124**	**1.9625**	**0.077 .**
	Growth Hormone	1	0.000672	0.00559	0.8849	0.415
	**Monocytes**	**1**	**0.002143**	**0.01783**	**2.8226**	**0.028 * **
	**Serum Glucose**	**1**	**0.001629**	**0.01355**	**2.1452**	**0.055 .**
	**HI Titer**	**1**	**0.001613**	**0.01342**	**2.1239**	**0.068 .**
	Hemoglobin	1	0.000489	0.00407	0.6446	0.639
	Residual	77	0.058473	0.48646		

Total	95	0.120203	1

Significance codes: 0 ‘***’ 0.001 ‘**’ 0.01 ‘*’ 0.05 ‘.’ 0.1 ‘ ’ 1

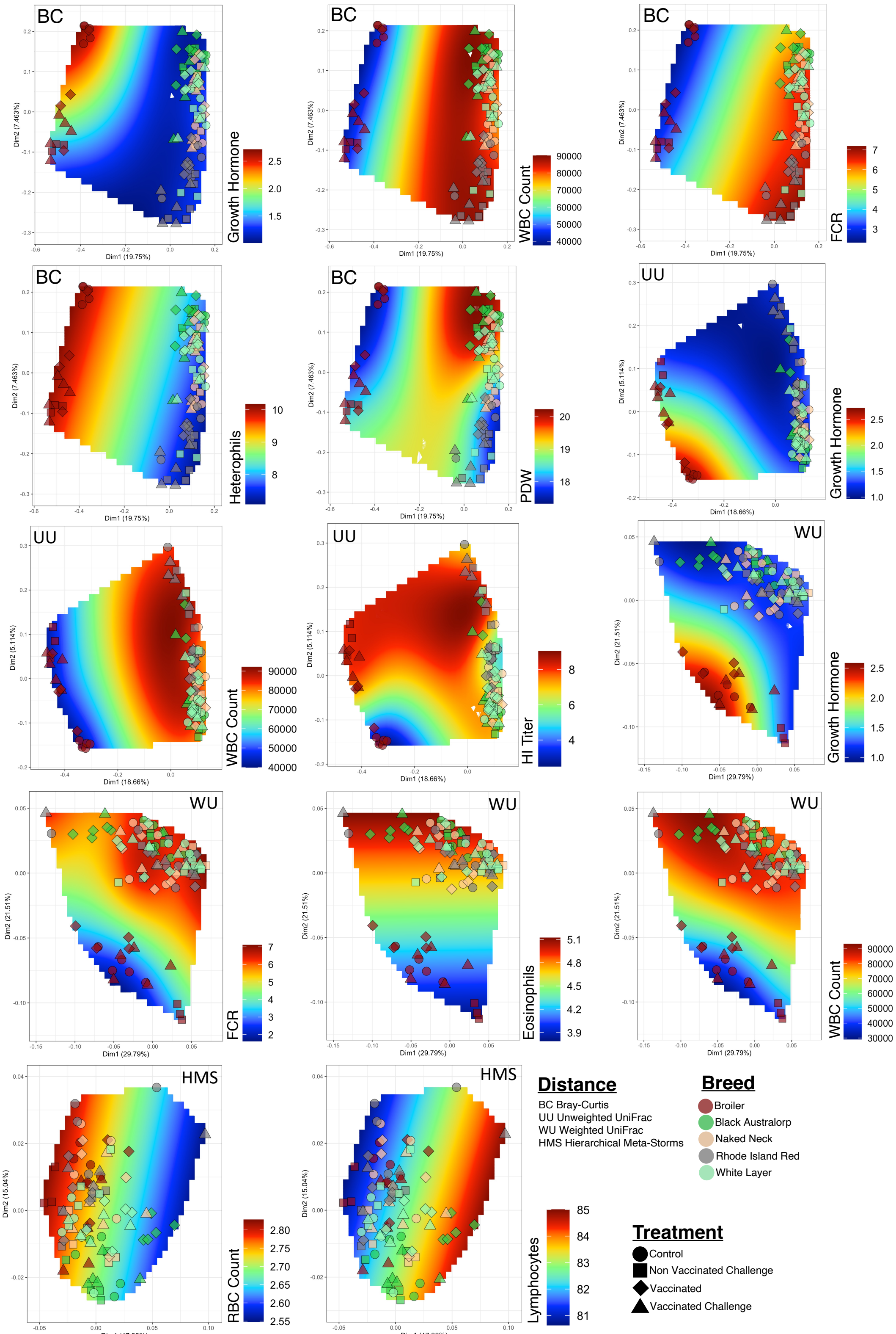

Supplementary Figure S16: Fitting of key parameters selected from PERMANOVA analysis in Supplementary Table S4. We fitted smooth surfaces of the covariates on ordination plot (PCoA in this case) using penalised splines. The method uses generalised additive model by regressing the covariate as C ~ S(Dim1,Dim2), where Dim1 and Dim2 are the ordination scores extracted from PCoA and S() is a spline function. We have only shown those covariates where the model fits i.e., $p < 0.0$

Supplementary Table S5: Genera found to be statistically significant (adjusted *p*-value significance cut-off of 0.05 and log2 fold change cut-off of 2) between multiple comparisons (using DESeq2) where the group they are upregulated in is in boldface. We have used the following naming conventions: **B.C** (Broiler Control); **B.V** (Broiler Vaccinated); **B.VCh** (Broiler Vaccinated Challenged); **B.NVCh** (Broiler Non-vaccinated Challenged); **NN.C** (Naked Neck Control); **NN.V** (Naked Neck Vaccinated); **NN.VCh** (Naked Neck Vaccinated Challenged); **NN.NVCh** (Naked Neck Non-vaccinated Challenged); **BA.C** (Black Australorp Control); **BA.V** (Black Australorp Vaccinated); **BA.VCh** (Black Australorp Vaccinated Challenged); **BA.NVCh** (Black Australorp Non-vaccinated Challenged); **RIR.C** (Rhode Island Red Control); **RIR.V** (Rhode Island Red Vaccinated); **RIR.VCh** (Rhode Island Red Vaccinated Challenged); **RIR.NVCh** (Rhode Island Red Non-vaccinated Challenged); **WL.C** (White Layer Control); **WL.V** (White Layer Vaccinated); **WL.VCh** (White Layer Vaccinated Challenged); and **WL.NVCh** (White Layer Non-vaccinated Challenged).

**Bacterial/Archaeal Genera**	**DESEQ Results** (upregulated in bold group)	**Implication in Literature**	**Reference**
Methanobrevibacter	**NN.V*** – NN.VCh; **RIR.C*** – RIR.V; **RIR.C*** – RIR.NVCh	Predominant methanogenic archaeal genus in the chicken ceca	(Yeoman et al. 2012; Luo et al. 2013; Luo et al. 2017)
Actinomyces	**NN.C* –** NN.NVCh; RIR.V **– RIR.VCh*; WL.C* –** WL.V; WL.V **– WL.VCh***	Gram-positive, anaerobic-to-microaerophilic bacteria, cause granulomas in humans/animals	(Hill et al. 1992)
Flaviflexus	**B.C* –** B.VCh**; RIR.C*-** RIR.NVCh; **RIR.C* –** RIR.VCh**; RIR.C* –** RIR.V	Detected and linked to multiple ARGs in layer farm	(Wang and Chai 2022)
Aeriscardovia	**BA.C* –** BA.NVCh; **BA.C* -** BA.VCh**; BA.C* -** BA.V**; BA.V*** - BA.VCh; **RIR.C* –** RIR.V; RIR.V **-** RIR.VCh; WL.C **– WL. NVCh*;** WL.C **- WL.VCh*;** W.V **– WL.VCh***	Increased the average daily gain and involved in immune modulation and gut health	(Farooq et al. 2022)
Bifidobacterium	BA.C **– BA.V*; BA.V* –** BA.VCh**;** NN.C **– NN.V*; NN.V* -** NN.VCh**; RIR.C* -** RIR.NVCh**; RIR.C* -** RIR.VCh**;** WL.C **- WL. NVCh*;** WL.C **- WL. VCh*;** WL.C **- WL. V***	Probiotic studied as alternative therapy for *Salmonella typhimurium* infection in broilers	(El-Sharkawy et al. 2020)
*Corynebacterium**	**BA.C* –** BA.NVCh; **B.C*** – B.NVCh; **B.C*** – B.VCh; **NN.C*** - NN.V; **RIR.C*** - RIR.NVCh; **RIR.C*** - RIR.VCh; **RIR.C*** - RIR.V; **WL.C*** - WL.NVCh; **WL.C*** - WL.VCh	Diphtheria outbreak reported in 15 weeks old white leghorn growers	(Enurah 2016)
*Dietzia**	**BA.C*-** BA.NVCh; **BA.C*-** BA.V**; B.C* –** B.NVCh; **B.C* -** B.NVCh; **NN.C* -**	Found abundant in outdoor layers and in 4	(Schreuder et al. 2020; Kubasova et al. 2022)

	NN.V; **RIR.C* -** RIR.NVCh; **RIR.C* -** RIR.VCh; RIR.V; **WL.C* -** WL.NVCh; **WL.C* -** WL.VCh; **WL.C* -** WL.V	month old broiler farm litter	
Brevibacterium	**B.C* –** B.NVCh; **B.C* –** B.VCh; **B.C* –** B.V; **NN.C* -** NN.V; **RIR.C* –** RIR.NVCh; **RIR.C*-** RIR.VCh; **RIR.C* -** RIR.V; **WL.C* -** WL.NVCh; **WL.C* -** WL.VCh; **WL.C* -** WL.V	Found abundant in 2 month old broiler farm litter	(Kubasova et al. 2022)
Oerskovia	WL.C **- WL.VCh***; WL.V **- WL.VCh***	Mesophile that was isolated from chicken faeces.	(Oerskovia merdavium Sa2CUA9 | DSM 112358 | BacDiveID:169777)
Brachybacterium	**B.C***- B.NVCh; **B.C*** – B.VCh; **B.C*** – B.V; **NN.C*** - NN.NVCh; **NN.C*** - NN.V; NN.V - **NN.VCh***; **RIR.C*** - RIR.NVCh; **RIR.C*** - RIR.VCh; **RIR.C*** – RIR.V; **RIR.V*** - RIR.VCh; **WL.C*** - WL.NVCh; **WL.C*** - WL.VCh; **WL.C*** - WL.V	Found abundant in 2 month old broiler farm litter	(Kubasova et al. 2022)
Gulosibacter	**RIR.C* -** RIR.NVCh; **RIR.C* -** RIR.VCh	Gram-positive, aerobic, non-spore-forming and non-motile bacteria, involved in breakdown of moilnate	https://en.wikipedia.org/wiki/Gulosibacter Duarte et al., 2011
Leucobacter	**B.C* -** BVCh; **RIR.C* -** RIR.VCh; RIR.VCh **- RIR.NVCh***; **RIR.V* -** RIR.VCh	Found abundant in braised chicken	(Liu et al. 2019)
Microbacterium	WL.C **- WL.NVCh***; WL.C **- WL.VCh***	Detected in high body weight chicken, positively correlated to fat metabolism	(Zhang et al. 2022)
Enteractinococcus	**RIR.C*** - RIR.NVCh; **WL.C*** - WL.NVCh; **WL.C*** - WL.VCh	Initially described as a benign constituent of the gut microbiota of adult poultry, now pathogenic strains have emerged as an important cause of skeletal disease in broilers	(Jung et al. 2018)
Glutamicibacter	**RIR.C* -** RIR.VCh	Gram positive, endospore negative, rod shaped, yellow pigmented, circular colony forming bacteria	(Santos et al. 2020)

Kocuria	BA.C **- BA.V***; **BA.V* -** BA.VCh; NN.C **- NN.V***; WL.C **- WL.V**; **WL.V -** WL.VCh	Human pathogens, predominantly isolated from chicken meat treated with oxalic acid	(Anang et al. 2006; Becker et al. 2008; Purty et al. 2013)
Yaniella	**NN.C*** - NN.NVCh; **NN.C*** - NN.V; NN.V - **NN.VCh***; **RIR.C*** - RIR.NVCh; **RIR.C*** - RIR.VCh; **RIR.C*** - RIR.V; **WL.C*** - WL.NVCh; **WL.C*** - WL.VCh; **WL.C***- WL.V	Found abundant in 4 month old broiler farm litter	(Kubasova et al. 2022)
Collinsella	**NN.C* -** NN.NVCh; **NN.C* -** NN.VCh; **NN.C* -** NN.V	An obligate anaerobe, non-motile, gram-positive rod bacterium, isolated from cecum of feral chickens	(Wongkuna et al. 2021)
Barnesiella	B.C **– B.NVCh***; B.V **– B.VCh*; NN.VCh* -** NN.NVCh; NN.V **- NN.VCh***; **WL.C* -** WL.VCh; **WL.V* -** WL.VCh	Gram-negative, obligate anaerobe, non-spore-forming, non-motile bacterium, isolated from chicken cecum	(Sakamoto et al. 2007)
Coprobacter	**BC*** – B.NVCh	Presented a decreased abundance during broiler's growth phase	(Clavijo et al. 2022)
Bacteroidales;F082;F082	**BA.C* -** BA.NVCh	Propionate producers, colonize layer chickens as probiotics	(Rios-Covian et al. 2017; Poudel et al. 2022)
Butyricimonas	**BA.C* -** BA.V	Efficiently transferred from hen to offspring, involved in butyrate production via lysine fermentation	(Medvecky et al. 2018)
Alloprevotella	**BA.C* -** BA.NVCh; **BA.VCh*** - BA.NVCh	Dominant fiber degrading genera in adult chicken gut	(Sun et al. 2021)
Prevotellaceae_Ga6A1_group	BA.C **- BA.VCh***; **BA.C* -** BA.V; BA.V **- BA.VCh***; **NN.C* -** NN.V; N.NV **- NN.VCh***; RIR.C **- RIR.NVCh***; RIR.C **- RIR.VCh***; RIR.V **- RIR.VCh***; **WL.C* -** WL.VCh; **WL.C* -** WL.V; WL.VCh **- WL.NVCh***	Not well-characterized, involved in the digestion of complex polysaccharides	(Crhanova et al. 2019)
Rikenellaceae	B.C – **B.VCh***	Found dominant in 50 day old chicken	(Sun et al. 2021)
Parabacteroides	B.C – **B.NVCh***; B.C – **B.VCh***; B.V – **B.VCh***	Foundational genera, transmitted from hen to chicks	(Kollarcikova et al. 2019)
Campylobacter	B.C – **B.NVCh***	Microaerobic thermophile, found abundant in indoor broiler chickens	(Bolton et al. 1997; Hazeleger et al. 1998; Varriale et al. 2022)

Helicobacter	BA.V - **BA.VCh***; **B.C* –** B.NVCh; **NN.VCh* -** NN.NVCh; NN.V - **NN.VCh***	Main colonizers of small intestine in chicken; associated with compromised performance	(Kollarcikova et al. 2019; Kempf et al. 2020)
Thermomicrobiales;JG30-KF-CM45;JG30-KF-CM45	**B.C*** - B.NVCh; **B.C*** - B.VCh	Non-filamentous nitrite oxidizing, thermophile	(Scheutz and Strockbine 2005; Sorokin et al. 2012; Sorokin et al. 2014)
Gastranaerophilales	**B.C*** - B.NVCh; **B.VCh* –** B.NVCh; **RIR.V* –** RIR.VCh	Positively correlates with the average daily gain and some immune responses in birds	(Li et al. 2020)
Mucispirillum	**WL.VCh* -** WL.NVCh	Stimulate mucus layer formation in intestinal tract, so enhances the gut health, found abundant in outdoor broiler chickens	(Varriale et al. 2022)
Bilophila	NN.C **- NN.V***	Sulfate reducing anaerobe, consistent member of the colonic flora of some animals	(McOrist et al. 2003; Ye et al. 2018)
Mailhella	NN.C **- NN.V***	Sulfate-reducers, abundant in laying hens	(Ricke et al. 2022)
Elusimicrobium	**BA.C*** - BA.V; NN.C - **NN.VCh***	Poorly represented microbiota, may appear in microbiota of adult hens	(Kempf et al. 2020)
Bacillus	**B.C* –** B.NVCh; **B.C* –** B.VCh; **B.V* –** B.VCh	Probiotic, helps in restoring many gut microbial genera	(Khan and Chousalkar 2020)
Planococcus	**NN.C* -** NN.V; **WL.C* -** WL.NVCh; **WL.C* –** WL.VCh	Multidrug resistant, gram positive, protease producer, found in chicken manure	(Dong et al. 2014; Ali et al. 2023)
[Clostridium]_innocuum_group	BA.C **- BA.V***; **BA.V* -** BA.VCh; NN.C **- NN.V***; **NN.V* -** NN.VCh; WL.C **- WL.V***; **WL.V* -** WL.VCh	Cellulolytic Firmicute, enhancing host health	(Leth et al. 2018)
Dielma	BA.C - **BA.NVCh***; WL.C - **WL.VCh***	Significantly correlated with short chain fatty acids production	(Qiu et al. 2022)
Faecalitalea	**BA.C*** - BA.NVCh; **BA.VCh*** - BA.NVCh; **RIR.C*** - RIR.V	Novel anaeobic rods, isolated from chicken cecum	(De Maesschalck et al. 2014)
Holdemania	BA.C **- BA.V***	Maintain gut health through lowering luminal pH and digestion of complex polysaccharides	(Khan and Chousalkar 2020)

Merdibacter	B.VCh – **B.NVCh***	Novel genus, detected in chicken gut metagenomes	(Gilroy et al. 2021)
Solobacterium	**BA.C*** - BA.NVCh; **BA.V* -** BA.VCh; NN.C - **NN.NVCh***; NN.C - **NN.VCh***; NN.C - **NN.V***; RIR.C - **RIR.VCh***; **RIR.VCh*** - RIR.NVCh; RIR.V - **RIR.VCh***	Gram positive, non-sporulated, anaerobic human pathogenic	(Kageyama and Benno 2000; Alauzet et al. 2021)
Turicibacter	BA.C **– BA.NVCh*;** BA.C **- BA.VCh*;** BA.C **- BA.V*;** RIR.C **- RIR.NVCh*;** RIR.C **- RIR.VCh*;** RIR.V **- RIR.VCh*;** WL.C **- WL.NVCh*;** WL.C **- WL.V*;** WL.VCh **- WL.NVCh*; WL.V* -** WL.VCh	Main colonizers of small intestine in chicken	(Kempf et al. 2020)
Aerococcus	**NN.C*** - NN.NVCh; **NN.C*** - NN.VCh; **RIR.C*** - RIR.NVCh; **RIR.C*** - RIR.VCh; **WL.C*** - WL.NVCh; **NN.C*** - NN.NVCh; **NN.C*** - NN.VCh; **NN.V*** - NN.VCh; **RIR.C*** - RIR.NVCh; **RIR.C*** - RIR.VCh; **RIR.C*** - RIR.V	Main Operational Bacterial Species detected in spoiled braised chicken	(Liu et al. 2019)
Facklamia	**NN.C*** - NN.NVCh; **NN.C*** - NN.VCh; **NN.C*** - NN.V; **RIR.C*** - RIR.NVCh; **RIR.C*** - RIR.VCh; **RIR.C*** - RIR.V; **WL.C*** - WL.NVCh; **WL.C*** - WL.VCh	Gram positive cocci, found as ileum microbiota of litter floor broilers	(LaClaire and Facklam 2000; Qiu et al. 2022)
Jeotgalibaca	**B.C* -** B.NVCh; **RIR.C* -** RIR.VCh	Detected in chicken gut metagenomes	(Gilroy et al. 2021)
Enterococcus	BA.C - **BA.V***; **BA.V*** - BA.VCh; **RIR.C*** - RIR.V; WL.C - **WL.NVCh***; WL.C - **WL.VCh***	Found in chicken small intestine	(Kempf et al. 2020)
Lactococcus	**B.C*** - B.NVCh	Studied as probiotic, on the selected microbial populations and intestinal morphology of 35-d-old broilers	(Lena et al. 2022)
Streptococcus	BA.C **- BA.V***; BA.V **- BA.VCh***; **B.C* -** B.NVCh; **B.VCh* -** B.NVCh; WL.C **- WL.NVCh***; WL.C **- WL.V***	Common inhabitants of the intestinal tract of animals and humans with potential for opportunistic infections	(Crispo et al. 2018)

Paenibacillus	NN.C - **NN.V***; **NN.V*** - NN.VCh	Detected in chicken gut metagenomes, may act as probiotic in broilers	(Gong et al. 2021; Gilroy et al. 2021)
Aliicoccus	**NN.C*** - NN.NVCh; **NN.C*** - NN.VCh; **NN.C*** - NN.V; **RIR.C*** - RIR.NVCh; **RIR.C*** - RIR.V; **WL.C*** - WL.NVCh; **WL.C*** - WL.VCh; **WL.C*** - WL.V	Gram positive aerobe, moderately halophilic, coccus-shaped, non-motile, non-sporulating bacteria	(Amoozegar et al. 2014)
Jeotgalicoccus	B.V - **B.VCh***; **NN.C*** - NN.NVCh; **NN.C*** - NN.V; **RIR.C*** - RIR.NVCh; **RIR.C*** - RIR.VCh; **RIR.V*** - RIR.VCh; **WL.C*** - WL.NVCh; **WL.C*** - WL.VCh; **WL.V*** - WL.VCh	Gram positive, non-motile, non-spore forming cocci, isolated from poultry houses	(Martin et al. 2011)
Salinicoccus	**RIR.C*** - RIR.NVCh	Found abundant in 4 month old broiler farm litter	(Kubasova et al. 2022)
Staphylococcus	**BA.C*** - BA.V; **NN.C*** - NN.NVCh; **NN.C*** - NN.V; NN.V - **NN.VCh***; **RIR.C*** - RIR.NVCh; **RIR.C*** - RIR.VCh; **RIR.C*** - RIR.V; **WL.C*** - WL.NVCh; **WL.C*** - WL.VCh; **WL.C*** - WL.V; **WL.V*** - WL.VCh	Found abundant in fresh and 4 month old broiler farm litter	(Kubasova et al. 2022)
Ruminiclostridium	RIR.C - **RIR.NVCh***	Found as highly connected taxa in co-occurrence network in cecum	(Feng et al. 2023)
Clostridia_vadinBB60_group	**B.C*** - B.NVCh; NN.C - **NN.NVCh***; NN.C - **NN.VCh***; NN.C - **NN.V***	Largely unclassified but predominant Firmicutes, in chicken cecum	(Ty et al. 2022)
Clostridium_sensu_stricto_1	BA.C - **BA.NVCh***; BA.C - **BA.V***; **BA.V*** - BA.VCh; **B.C*** - B.NVCh; **B.C*** - B.VCh; **B.V*** - B.VCh; NN.V - **NN.VCh***; RIR.C - **RIR.NVCh***; RIR.C - **RIR.VCh***; RIR.V - **RIR.VCh***; WL.C - **WL.NVCh***; WL.C - **WL.V***; WL.VCh - **WL.NVCh***	Main colonizers of small intestine in chicken	(Rychlik 2020)
Eubacteriaceae	BA.C - **BA.V***; **BA.V*** - BA.VCh	Efficiently transferred from hen to offspring	(Oakley et al. 2014; Rychlik 2020)
[Eubacterium]_ventriosum_group	**B.C*** - B.NVCh; **NN.V*** - NN.VCh		
Blautia	NN.V - **NN.VCh***; RIR.C - **RIR.NVCh***; RIR.C - **RIR.VCh***; RIR.V - **RIR.VCh***	Anaerobic, have potential of probiotics, abundance increased with the age of chickens	(Xi et al. 2019; X. Liu et al. 2021)

Lachnospiraceae;CHKCI001	**BA.C*** - BA.NVCh; **BA.C*** - BA.VCh; **BA.C*** - BA.V; NN.V - **NN.VCh***; **RIR.C*** - RIR.V; **RIR.VCh*** - RIR.NVCh; RIR.V - **RIR.VCh***; **WL.C*** - WL.V; WL.VCh - **WL.NVCh***	Abundant in poultry and particularly effective in degrading cellulose/indigestible polysaccharides	(Biddle et al. 2013)
*Frisingicoccus**	BA.C - **BA.NVCh***; BA.VCh - **BA.NVCh***; **B.C*** - B.NVCh; **B.VCh*** - B.NVCh; NN.C - **NN.V***; **NN.V*** - NN.VCh; **RIR.V*** - RIR.VCh; WL.C - **WL.VCh***; **WL.VCh*** - WL.NVCh	Found abundant in high weight male chickens	(Liu et al. 2023; Ali et al. 2023)
Lachnoclostridium	RIR.C - **RIR.VCh***; RIR.V - **RIR.VCh***	SCFA producers, predominant in last stage of the broiler production cycle.	(Clavijo et al. 2022)
Lachnospiraceae_UCG-010	NN.C - **NN.V***	Positively correlated with feed efficiency of chickens through SCFA production	(J. Liu et al. 2021)
Marvinbryantia	**B.C*** - B.NVCh	Decreased abundance noticed in *Eimeria* infected chicken group	(Memon et al. 2022)
Roseburia	**B.C*** - B.NVCh; **B.VCh*** - B.NVCh	Cellulolytic Firmicute, enhancing host health	(Leth et al. 2018)
Shuttleworthia	**RIR.C*** - RIR.NVCh; **RIR.VCh*** - RIR.NVCh; RIR.V - **RIR.VCh***; **WL.C*** - WL.NVCh	Decreased abundance noticed in *Eimeria* infected chicken group	(Memon et al. 2022)
Tuzzerella	**B.C*** - B.NVCh; **B.C*** - B.VCh; **NN.C*** - NN.VCh; **NN.C*** - NN.V; **B.C*** - B.VCh; **B.C*** - B.V; B.VCh - **B.NVCh***; WL.C - **WL.VCh***	Found as part of stable ensemble in chicken gut, over a period of 32 days	(Ameer et al., 2023)
Monoglobus	**B.C*** - B.NVCh	Have ability to ferment dietary fiber, found strongly linked with some SCFAs in ducks ceca	(Goris et al. 2021; Hao et al. 2022)
[Clostridium]_methylpentosum_group	B.C - **B.NVCh***	Cellulolytic Firmicute, enhancing host health	(Leth et al. 2018)
[Eubacterium]_coprostanoligenes_group	**BA.C*** - BA.NVCh; **BA.V*** - BA.VCh	Found in co-occurrence netwrok in jejunum chymus and mucosa of low body weight chickens	(Farkas et al. 2022)
Butyricicoccus	B.VCh - **B.NVCh***	Butyrate producers, isolated from chicken caecal content	(Wang et al. 2020)

Butyricicoccaceae;UCG-008	**BA.C*** - BA.V; NN.VCh - **NN.NVCh***; **NN.V*** - NN.VCh; **RIR.C*** - RIR.NVCh; **RIR.C*** - RIR.VCh; **RIR.V*** - RIR.VCh;	Butyrate producers, isolated from chicken caecal content	(Wang et al. 2020)
Butyricicoccaceae;UCG-009	**B.C*** - B.NVCh	Butyrate producers, isolated from chicken caecal content	(Wang et al. 2020)
Flavonifractor	B.C - **B.NVCh***; B.VCh - **B.NVCh***; RIR.C - **RIR.NVCh***; WL.C - **WL.V***	Non-spore forming, can produce butyrate by lysine fermentation or succinate reduction	(Rychlik 2020)
Intestinimonas	**B.C*** - B.NVCh; **B.C*** - B.VCh	Abundant butyrate producer in chicken ceca, also found as core member	(Kläring et al. 2013; Ijaz et al. 2018)
Oscillospira	B.V - **B.VCh***	Gram positive, rods, capable of producing short-chain fatty acids	(Yang et al. 2021)
Oscillospiraceae;UCG-007	**B.V*** - B.VCh	Found as abundant taxa having stable relative abundance with minimum coefficient of variation, in chicken cecum	(Ameer et al. 2023)
[Eubacterium]_siraeum_group	**NN.C*** - NN.V; **WL.C*** - WL.VCh	An important serpin, play role in host:bacterium crosstalk	(Yang et al. 2019; Mkaouar et al. 2021)
Angelakisella	RIR.C - **RIR.V***; **RIR.V*** - RIR.VCh	Found in low abundance in chicken feaces	(Borey et al. 2021)
Ruminococcaceae;CAG-352	**RIR.VCh*** - RIR.NVCh	Cellulolytic Firmicute, enhancing host health	(Rakoff-Nahoum et al. 2016)
Caproiciproducens	BA.C - **BA.NVCh***; BA.C - **BA.V***; NN.C - **NN.V***	Butyrate producer, found differentially abundant in different groups of broilers	(Kim et al. 2015; Alvarenga et al. 2023)
Ruminococcaceae;DTU089	**WL.C*** - WL.VCh; **WL.V*** - WL.VCh	Cellulolytic Firmicute, enhancing host health	(Rakoff-Nahoum et al. 2016)
Faecalibacterium	RIR.C - **RIR.VCh***; RIR.V - **RIR.VCh***; **WL.C*** - WL.V	Butyrate-producers in both chickens and humans	(Rychlik 2020)
Fournierella	**BA.V*** - BA.VCh; B.C - **B.NVCh***; B.VCh - **B.NVCh***	Related to improved chicken gut health and increased feed intake	(Ameer et al. 2023)
Negativibacillus	B.V - **B.VCh***	Found significantly higher in low body weight chickens caecum chymus	(Farkas et al. 2022)
Paludicola	**B.C*** - B.NVCh; **B.C*** - B.VCh; RIR.VCh - **RIR.NVCh***	A novel chitinolytic anaerobe, positively correlated with the	(Li et al. 2017; Wang et al. 2023)

		market weight of chickens	
Ruminococcus	**B.C*** - B.NVCh; **B.C*** - B.VCh; RIR.C - **RIR.NVCh***	Cellulolytic *Firmicute*, enhancing host health	(Rakoff-Nahoum et al. 2016)
Subdoligranulum	**BA.V*** - BA.VCh	Found as part of caecal microbiota related to the market weights of Chinese chickens at 160 days of age	(A. Li et al. 2022)
Oscillospirales;UCG-010;UCG-010	**RIR.V*** - RIR.VCh	Probiotic genera associated with improved broiler performance	(Ameer et al. 2023)
[Eubacterium]_nodatum_group	**BA.C*** - BA.NVCh; **BA.V*** - BA.VCh	periodontitis-causing, found abundant in jejunum of Eimeria infected group of broiler chicken	(Haffajee et al. 2006; Campos et al. 2023)
Anaerovoracaceae;Family_XIII_UCG-001	**WL.C*** - WL.NVCh	Found differentially abundant across different dietary treatments, in intestinal microbiota of piglets	(Reyer et al. 2021)
Romboutsia	WL.C - **WL.NVCh***; WL.VCh - **WL.NVCh***; **WL.V*** - WL.VCh	Main colonizers of small intestine in chicken	(Rychlik 2020)
Gallicola	**BA.C*** - BA.NVCh; **BA.C*** - BA.VCh; **BA.C*** - BA.V; **RIR.C*** - RIR.NVCh; **RIR.C*** - RIR.VCh	Non-spore-forming, obligate anaerobic chicken gut inhabitant	(Ezaki 2015)
Gottschalkia	**RIR.V*** - RIR.VCh; WL.C - **WL.NVCh***	Found abundant in chickens exposed to heavy metals such as Cr	(M.H. Li et al. 2022)
Megamonas	**RIR.C*** - RIR.NVCh; **RIR.C*** - RIR.V; **RIR.VCh*** - RIR.NVCh; RIR.V - **RIR.VCh***	Anaerobic commensals, identified as stable chicken gut microbiome	(Ameer et al. 2023)
Megasphaera	BA.C - **BA.V***; **BA.V*** - BA.VCh; **NN.C*** - NN.V; NN.V - **NN.VCh***; RIR.V - **RIR.VCh***	Butyrate-producers in both chickens and humans	(Rychlik 2020)
Veillonella	B.C - **B.NVCh***; B.VCh - **B.NVCh***; WL.C - **WL.VCh***; **WL.VCh*** - WL.NVCh; WL.V - **WL.VCh***	Propionate producers, increased in abundance in H9N2 infected chickens	(Ruddon and Arbor 2010)Ruddon, 2010
Fusobacterium	**NN.C*** - NN.VCh; **NN.V*** - NN.VCh; WL.C - **WL.VCh***; WL.C - **WL.V***; **WL.VCh*** - WL.NVCh	Found abundant across indoor and outdoor housed laying chickens	(Schreuder et al. 2021)

Candidatus_Saccharimonas	BA.V - **BA.VCh***; RIR.C - **RIR.NVCh***; RIR.C - **RIR.VCh***; RIR.C - **RIR.V***	Found negatively correlated with *Eschrichia-Shigella* when chickens fed or not fed with insect larvae	(Detilleux et al. 2022)Detilleux et al., 2022
Saccharimonadales	**BA.C*** - BA.NVCh; **BA.C*** - BA.V; BA.V - **BA.VCh***; NN.C - **NN.VCh***; **NN.VCh*** - NN.NVCh; WL.VCh - **WL.NVCh***	Found abundant in Ross broilers	(Wu et al. 2021)
Devosia	**RIR.V*** - RIR.VCh	Decreased deoxynivalenol residues growing-finishing pigs tissues	(Li et al. 2018)
Ochrobactrum	WL.C - **WL.NVCh***; WL.C - **WL.VCh***	Highlighted as environment friendly alternative to treat poultry feathers	(de Menezes et al. 2023)
Paenochrobactrum	NN.C - **NN.NVCh***; NN.C - **NN.V***; NN.VCh - **NN.NVCh***; **NN.V*** - NN.VCh; **RIR.C*** - RIR.VCh; **RIR.V*** - RIR.VCh; WL.C - **WL.NVCh***; WL.C - **WL.VCh***; WL.V - **WL.VCh***	Gram negative, non spore forming, non motile rod, isolated from a chicken	(Kämpfer et al. 2014)
Pseudochrobactrum	NN.C - **NN.NVCh***; NN.C - **NN.V***; NN.VCh - **NN.NVCh***; **NN.V*** - NN.VCh; **RIR.V*** - RIR.VCh; WL.C - **WL.NVCh***; WL.C - **WL.VCh***	Novel keratinolytic bacterium, possessing high affinity for black feathers, isolated from chicken manure	(Yusuf et al. 2020)
Gemmobacter	NN.C - **NN.NVCh***; NN.VCh - **NN.NVCh***	Denitrifying methane oxidizers of interest because of their metabolic pathways and habitats	(Jin et al. 2021)
Paracoccus	NN.C - **NN.NVCh***; NN.VCh - **NN.NVCh***; WL.C - **WL.NVCh***; WL.C - **WL.VCh***; WL.V - **WL.VCh***	Species are studied as a potential feed additive for laying hens to enhance yolk color	(Conradie et al. 2018)
Anaerobiospirillum	BA.V - **BA.VCh***; NN.C - **NN.VCh***; **RIR.C*** - RIR.NVCh	Strict anaerobes, abundant in caecal microbiota of adult hens	(Rychlik 2020)
Oligella	BA.V - **BA.VCh***	Found in correlation with other genera in low body weight chickens, associated with urological infections in humans	(Baqi and Mazzulli 1996; Farkas et al. 2022)

Parasutterella	**WL.C*** - WL.NVCh	Strict anaerobe, commonly found in chicken ceacum, specifically in adult hens	(Rychlik 2020)
Escherichia-Shigella	BA.C - **BA.NVCh***; BA.C - **BA.V***; **RIR.C*** - RIR.NVCh; **RIR.C*** - RIR.VCh; WL.C - **WL.NVCh***; WL.C - **WL.VCh***; WL.C - **WL.V***	Main colonizers of small intestine in chicken; associated with compromised performance; significantly increased in NDV infected chickens	(Kollarcikova et al. 2019; Rychlik 2020; Tong et al. 2022)
Gallibacterium	B.C - **B.NVCh***; B.VCh - **B.NVCh***	Found dominant in chicken crop; abundant across indoor and outdoor housed laying chickens, found in chicken crop	(Rychlik 2020; Schreuder et al. 2021)
Acinetobacter	**B.C*** - B.NVCh; **B.C*** - B.VCh	Multidrug resistant commensal gut microbes in chicken, found in peak and late laying periods	(Jochum et al. 2021)
Psychrobacter	**NN.V*** - NN.VCh	Found abundant in braised chicken	(Liu et al. 2019)
Brachyspira	BA.C - **BA.NVCh***; RIR.C - **RIR.V***	Opportunistic pathogen colonizing the gastrointestinal tract of poultry (ileum, caeca, and colon), cause avian intestinal spirochaetosis in poultry	(Le Roy et al. 2015)
Treponema	**NN.C*** - NN.V; NN.V - **NN.VCh***; RIR.C - **RIR.VCh***; RIR.V - **RIR.VCh***; **WL.C*** - WL.VCh; **WL.C*** - WL.V; WL.VCh - **WL.NVCh***	Involved in fibre digestion, poorly represented in chikens, more common in pigs	(Kubasova et al. 2018; Rychlik 2020)
Chlamydia	NN.C - **NN.VCh***; **NN.VCh*** - NN.NVCh; NN.V - **NN.VCh***	Zoonotic intracellular bacterium, reported in many bird species including poultry	(Heijne et al. 2021)
Kiritimatiellae;WCHB1-41;WCHB1-41;WCHB1-41	**B.C*** - B.NVCh; **B.C*** - B.VCh; NN.C - **NN.V***; NN.VCh - **NN.NVCh***; **NN.V*** - NN.VCh; RIR.C - **RIR.V***; **RIR.V*** - RIR.VCh	Non dominated phylum, found in Rhinopithecus; found abundant in cecum of fasting guinea pig	(Xi et al. 2023; Frias et al. 2023)
Victivallales;vadinBE97;vadinBE97	NN.C - **NN.VCh***; NN.C - **NN.V***; **RIR.V*** - RIR.VCh	0.40% of the total abundance of genera, exhibited significant positive correlations with other genera, in chicken ceaca, may	(Wen et al. 2023)

		regulate intramuscular fat trough SCFAs production	
Victivallaceae	BA.V - **BA.VCh***; NN.C - **NN.NVCh***; NN.C - **NN.V***; WL.V - **WL.VCh***	Found as commensals, and associated with improved feed efficiency in poultry	(Singh et al. 2012)
Victivallis	NN.C - **NN.V***; RIR.C - **RIR.V***	Found as an important component of heritable chicken gut microbiome, varied abundance noticed in *Pullorum* positive and negative chickens	(Ding et al. 2021)
Cerasicoccus	BA.C - **BA.NVCh***; BA.VCh - **BA.NVCh***; B.C - **B.NVCh***; B.C - **B.VCh***; B.V - **B.VCh***	Increased abundance noticed in feed restricted laying hen's gut	(Artdita et al. 2021)
Pedosphaeraceae;DEV114	NN.C - **NN.NVCh***; NN.C - **NN.V***	Found abundant in conventional diet group of Nelore bulls	(Andrade et al. 2022)
*Akkermansia**	BA.C - **BA.NVCh***; BA.V - **BA.VCh***; B.C - **B.VCh***; **B.VCh*** - B.NVCh; B.V - **B.VCh***; NN.VCh - **NN.NVCh***; **NN.V*** - NN.VCh; RIR.C - **RIR.NVCh***; RIR.C - **RIR.V***; **RIR.V*** - RIR.VCh	Mucolytic bacteria, oral inoculation promoted the development of Necrotic enteritis and modulated the jejunal microbiota of chickens	(Derrien et al. 2017; Yang et al. 2022)
Bacteria;WPS-2;WPS-2;WPS-2;WPS-2;WPS-2	**NN.C*** - NN.V; NN.V - **NN.VCh***; RIR.C - **RIR.NVCh***; **WL.C*** - WL.V; WL.V - **WL.VCh***	--	--
Rhabditida	NN.C - **NN.VCh***; **WL.C*** - WL.V	Saprophytic free-living nematodes, parasitizing humans and animals, causing fatal infections	(Guerrant et al. 2006)

Supplementary Table S6: MetaCyc pathways along with function, found to be statistically significant (adjusted *p*-value significance cut-off of 0.05 and log2 fold change cut-off of 2) between multiple comparisons (using DESeq2) where the group they are upregulated in is in boldface. We have used the following naming conventions: **B.C** (Broiler Control); **B.V** (Broiler Vaccinated); **B.VCh** (Broiler Vaccinated Challenged); **B.NVCh** (Broiler Non-vaccinated Challenged); **NN.C** (Naked Neck Control); **NN.V** (Naked Neck Vaccinated); **NN.VCh** (Naked Neck Vaccinated Challenged); **NN.NVCh** (Naked Neck Non-vaccinated Challenged); **BA.C** (Black Australorp Control); **BA.V** (Black Australorp Vaccinated); **BA.VCh** (Black Australorp Vaccinated Challenged); **BA.NVCh** (Black Australorp Non-vaccinated Challenged); **RIR.C** (Rhode Island Red Control); **RIR.V** (Rhode Island Red Vaccinated); **RIR.VCh** (Rhode Island Red Vaccinated Challenged); **RIR.NVCh** (Rhode Island Red Non-vaccinated Challenged); **WL.C** (White Layer Control); **WL.V** (White Layer Vaccinated); **WL.VCh** (White Layer Vaccinated Challenged); and **WL.NVCh** (White Layer Non-vaccinated Challenged).

**Bacterial/Archaeal Pathways**	**DESEQ Results** (upregulated in bold group)	**Function**	**Expected Taxonomic Range**
3-HYDROXYPHENYLACETATE-DEGRADATION-PWY	**B.C*** - B.NVCh	Carbon and energy source	Pseudomonadota
AST-PWY	WL.C- **WL.VCh***	Utilize arginine and produce ammonia	Pseudomonadota
CATECHOL-ORTHO-CLEAVAGE-PWY	**B.C*** - B.NVCh; **NN.C*** - NN.V; **RIR.C*** - RIR.NVCh; **RIR.C*** - RIR.VCh; **RIR.C*** - RIR.V; **WL.C***- WL.NVCh	Degrade catechol through ortho-cleave	Actinomycetota, Pseudomonadota
CHLOROPHYLL-SYN	**B.C*** - B.NVCh; **B.VCh*** - B.NVCh; **B.C*** - B.NVCh; **B.VCh*** - B.NVCh; **NN.C*** - NN.V; **RIR.C** - RIR.NVCh; **RIR.C*** - RIR.VCh; **RIR.C*** - RIR.V; **RIR.V*** -RIR.VCh; **WL.C***- WL.NVCh; **WL.C***- WL.VCh	Synthesize enterobactin from 2,3-dihydroxybenzoate and L-serine	Bacteria, Euglenozoa, Rhodophyta, Viridiplantae
ENTBACSYN-PWY*	WL.C- **WL.VCh***	Synthesize enterobactin from 2,3-dihydroxybenzoate and L-serine	Pseudomonadota
GALLATE-DEGRADATION-I-PWY*	**RIR.C*** - RIR.VCh; RIR.VCh - **RIR.NVCh***; **RIR.V*** -RIR.VCh	Degradation of aromatic compounds such as gallate	Bacteria
GALLATE-DEGRADATION-II-PWY*	**RIR.C*** - RIR.VCh; RIR.VCh - **RIR.NVCh***; **RIR.V*** -RIR.VCh	Degradation of aromatic compounds such as gallate	Bacteria
GLUCOSE1PMETAB-PWY	B.V - **B.VCh***	Degrade glucose and glucose-1-phosphate to produce carbon and energy	Archaea, Bacteria, Eukaryota

HCAMHPDEG-PWY	**RIR.C*** - RIR.NVCh; **RIR.C*** - RIR.VCh	Utilize aromatic acids as a carbon and energy source	Pseudomonadota
KDO-NAGLIPASYN-PWY	WL.C- **WL.VCh***	Lipid A biosynthesis	
LIPASYN-PWY	**B.C*** - B.NVCh; **B.VCh*** - B.NVCh; **RIR.C*** - RIR.NVCh; **RIR.C*** - RIR.VCh; **RIR.V*** - RIR.VCh; WL.C- **WL.NVCh***; WL.C- **WL.VCh***; WL.C- **WL.V***	Fatty acid and lipid degradation	Archaea, Bacteria, Eukaryota
METHYLGALLATE-DEGRADATION-PWY	**RIR.C*** - RIR.VCh; RIR.VCh - **RIR.NVCh***; **RIR.V*** -RIR.VCh	Aromatic compound degradation	Bacteria
NADSYN-PWY	**B.C*** - B.NVCh; **B.C*** - B.VCh	Nicotinamide adenine dinucleotide biosynthesis	Bacteria, Eukaryota
ORNDEG-PWY	**RIR.C*** - RIR.NVCh; **RIR.C*** - RIR.VCh	Amide, amidine, amine, and polyamine degradation	Bacteria
P101-PWY	**B.C*** - B.NVCh; **RIR.C*** - RIR.NVCh; **RIR.C*** - RIR.VCh; **WL.C***- WL.NVCh; **WL.C***- WL.VCh;	Amide, amidine, amine, and polyamine biosynthesis	Bacteria
P381-PWY	**BA.C*** - BA.V; **RIR.C*** - RIR.VCh	Adenosylcobalamin biosynthesis II, vitamin B12 biosynthesis	Archaea, Bacteria
P621-PWY	B.C - **B.VCh***	Caprolactam degradation	Bacteria
PROTOCATECHUATE-ORTHO-CLEAVAGE-PWY	**RIR.C*** - RIR.VCh	Aromatic compound degradation	Bacteria
PWY-1422	**B.C*** - B.NVCh; **B.VCh*** - B.NVCh	Tocopherol biosynthesis	Chlorophyta, Cyanobacteriota, Phaeophyceae, Rhodophyta, Viridiplantae
PWY-1882	**B.C*** - B.NVCh; **RIR.V*** - RIR.VCh	Aromatic compound biosynthesis	Spermatophyta
PWY-3661	**B.C*** - B.NVCh; **B.VCh*** - B.NVCh; **NN.C*** - NN.V; **RIR.C*** - RIR.NVCh; **RIR.C*** - RIR.VCh; **RIR.C*** - RIR.V	Amide, amidine, amine, and polyamine degradation	Archaea, Bacteria
PWY-3801	**B.C*** - B.NVCh; **B.VCh*** - B.NVCh	Carbohydrate degradation	Cyanobacteriota, Viridiplantae
PWY-3941	**B.C*** - B.NVCh; **B.C*** - B.VCh; **RIR.V*** -RIR.VCh	Amino acid biosynthesis	Bacteria, Viridiplantae
PWY-4361	**B.C*** - B.NVCh; **B.C*** - B.VCh	Methionine salvage pathway	Archaea, Bacteria, Embryophyta, Metazoa
PWY-4722	WL.VCh - **WL.NVCh***	Creatinine degradation II	Bacteria

PWY-5178	**NN.C*** - NN.NVCh; **NN.C*** - NN.V; **RIR.C*** - RIR.NVCh**;** **RIR.C*** - RIR.VCh; **RIR.C*** - RIR.V; **WL.C*-** WL.NVCh	Aromatic compound degradation	Pseudomonadota
PWY-5181	**B.C*** - B.NVCh; **B.C*** - B.VCh	Degradation of toluene	Pseudomonadota
PWY-5183	**RIR.C*** - RIR.NVCh; **RIR.C*** - RIR.VCh	Aerobic degradation of toluene	Pseudomonadota
PWY-5392	**B.C*** - B.NVCh; **B.C*** - B.VCh; **B.V*** - B.VCh	Reductive tricarboxylic acid cycle	Aquificae
PWY-5417	**B.C*** - B.NVCh; **B.C*** - B.VCh; **RIR.C*** - RIR.NVCh; **RIR.C*** - RIR.VCh; **WL.C***- WL.NVCh	Aromatic compound degradation	Fungi, Pseudomonadota
PWY-5419	**B.C*** - B.NVCh; **B.C*** - B.VCh	Catechol degradation to 2-hydroxypentadienoate II	Actinomycetota, Pseudomonadota
PWY-5420	**B.C*** - B.NVCh; **B.C8** - B.VCh	Catechol degradation	Actinomycetota, Pseudomonadota
PWY-5430	**B.C*** - B.NVCh; **RIR.C*** - RIR.NVCh; **RIR.C* -** RIR.VCh	Benzoate degradation	Acinetobacter, Pseudomonas
PWY-5431	**B.C*** - B.NVCh; **B.C*** - B.VCh; **RIR.C*** - RIR.NVCh; **RIR.C*** - RIR.VCh; **WL.C***- WL.NVCh	Aromatic compound degradation	Pseudomonadota
PWY-5499	**B.C*** - B.NVCh; **B.C*** - B.VCh; **RIR.C*** - RIR.VCh	Vitamin B6 degradation	Bacteria
PWY-5529	**B.C*** - B.NVCh	Chlorophyll a biosynthesis	Chlorobiales, Chromatiales, Rhodobacterales, Rhodospirillales
PWY-5531	**B.C*** - B.NVCh; **B.VCh* -** B.NVCh	Anaerobic chlorophyllide biosynthesis	Bacteria
PWY-5647	**B.C*** - B.NVCh; **B.C*** - B.VCh	Nitrobenzoate degradation	Bacteria
PWY-5651	**B.C*** - B.NVCh; **B.C*** - B.VCh	L-tryptophan degradation	Bacteria, Fungi
PWY-5654	**B.C*** - B.NVCh; **B.C*** - B.VCh	Carboxylic acid degradation	Bacteria
PWY-5655	**B.C*** - B.NVCh; **B.C*** - B.VCh	L-tryptophan degradation	Bacteria
PWY-5656	BA.C **- BA.NVCh***; B.C - **B.NVCh***; B.C **- B.VCh***; B.V - **B.VCh***	Mannosylglycerate biosynthesis I	Archaea, Bacteria
PWY-5741	**RIR.V*** -RIR.VCh	Ethylmalonyl-CoA pathway	Bacteria
PWY-6071	**B.C*** - B.NVCh; **B.C*** - B.VCh	Phenylethylamine degradation	Bacteria
PWY-6107	B.C **- B.NVCh***; B.C **- B.VCh***; B.V - **B.VCh***; **RIR.C* -** RIR.VCh; **RIR.V*** -RIR.VCh	Chlorosalicylate degradation	Bacteria
PWY-6113	B.C **- B.VCh***; **B.VCh* -** B.NVCh; B.V **- B.VCh*;**	Mycolate biosynthesis	Mycobacteriaceae

PWY-6143	BA.V - **BA.NVCh***; B.C - **B.NVCh***	CMP-pseudaminate biosynthesis	Bacteria
PWY-6167	**B.C*** - B.NVCh	Flavin biosynthesis II in archaea	Archaea
PWY-6174	**BA.C*** - BA.V	Mevalonate pathway II in haloarchaea	Chloroflexota, Halobacteria
PWY-6182	**B.C*** - B.NVCh; **B.C*** - B.VCh; **RIR.C*** - RIR.VCh	Salicylate degradation	Bacteria
PWY-6185	**B.C*** - B.NVCh; **B.C*** - B.VCh	4-methylcatechol degradation	Bacteria
PWY-6284	B.C **- B.VCh***; **B.VCh*** - B.NVCh; B.V **- B.VCh***	Unsaturated fatty acids biosynthesis	Bacteria
PWY-6285	B.C - **B.VCh***; **B.VCh*** - B.NVCh; B.V **- B.VCh***	Fatty acids biosynthesis	Bacteria
PWY-6339	**RIR.V*** -RIR.VCh	Syringate degradation	Bacteria
PWY-6383	**NN.C*** - NN.V; **RIR.C*** - RIR.NVCh; **RIR.C*** - RIR.VCh; **RIR.C*** - RIR.V; **WL.C***- WL.NVCh	Poly-cis decaprenyl phosphate biosynthesis	Mycobacteriaceae
PWY-6397	**B.C*** - B.NVCh; **NN.C* -** NN.V; **RIR.C* -** RIR.NVCh; **RIR.C* -** RIR.VCh; **RIR.C*** - RIR.V; **WL.C***- WL.NVCh; **WL.C***- WL.VCh	Peptidoglycan complex biosynthesis	Mycobacteriaceae
PWY-6404	**BA.C*** - BA.V; **B.C*** - B.NVCh; **RIR.V*** -RIR.VCh	Mycolyl-arabinogalactan peptidoglycan complex biosynthesis	Mycobacteriaceae
PWY-6505	**B.C*** - B.NVCh; **B.C*** - B.VCh	L-tryptophan degradation	Bacteria
PWY-6562	**B.C*** - B.NVCh; **B.C*** - B.VCh	Norspermidine biosynthesis	Vibrionaceae
PWY-6690	**RIR.C*** - RIR.NVCh; **RIR.C*** - RIR.VCh	Cinnamate and 3-hydroxycinnamate degradation to 2-hydroxypentadienoate	Bacteria
PWY-6713	**B.C*** - B.NVCh; **B.C*** - B.VCh	L-rhamnose degradation II	Bacteria, Fungi
PWY-6876	RIR.C - **RIR.NVCh***; RIR.C - **RIR.VCh***; RIR.V **-RIR.VCh8**	Isopropanol biosynthesis	Clostridium, Escherichia coli
PWY-6906	**RIR.V*** -RIR.VCh; WL.C- **WL.NVCh***; WL.C- **WL.VCh***; WL.C- **WL.V***	Chitin derivatives degradation	Vibrionaceae
PWY-6957	**RIR.V*** -RIR.VCh	Mandelate degradation to acetyl-CoA	Pseudomonadota
PWY-6992	**B.C*** - B.NVCh; **B.C* -** B.VCh; **RIR.C* -** RIR.VCh; RIR.VCh - **RIR.NVCh***; **RIR.V*** -RIR.VCh;	1,5-anhydrofructose degradation	Bacteria

	**WL.C***- WL.NVCh; **WL.C***- WL.VCh		
PWY-7007	**RIR.C*** - RIR.VCh	Methyl ketone biosynthesis	Escherichia coli, Micrococcus luteus
PWY-7031	BA.V - **BA.NVCh***; B.C - **B.NVCh***	Protein N-glycosylation	Campylobacter
PWY-7084	**B.C*** - B.NVCh; **RIR.V*** - RIR.VCh	Nitrifier denitrification	Bacteria
PWY-7159	**B.C*** - B.NVCh; **B.VCh*** - B.NVCh	Divinyl-chlorophyllide a biosynthesis	Bacteria, Bryophyta, Chlorophyta, Cyanobacteriota, Polypodiopsida
PWY-7209	NN.C - **NN.V***; RIR.C - **RIR.V***; **RIR.V*** -RIR.VCh	Pyrimidine ribonucleosides degradation	Archaea, Bacteria, Metazoa
PWY-722	**RIR.V*** -RIR.VCh	Nicotinate degradation I	Pseudomonadota
PWY-7295	BA.C - **BA.NVCh***; B.C - **B.NVCh***; B.C - **B.VCh***; B.V - **B.VCh***	L-arabinose degradation IV in archaea	Archaea
PWY-7347	**B.C*** - B.NVCh; **B.VCh*** - B.NVCh	Sucrose biosynthesis	Methylobacter, Methylomicrobium, Methylophaga, Methylophilaceae
PWY-7373	BA.V - **BA.NVCh***	Demethylmenaquinol-6 biosynthesis II	Bacteria
PWY-7376	**BA.C*** - BA.V; **RIR.C*** - RIR.VCh	Adenosylcobalamin biosynthesis II (aerobic)	Archaea, Bacteria
PWY-7391	**RIR.C*** - RIR.NVCh	Isoprene biosynthesis	Enterococcus, Methanosarcina
PWY-7446	WL.C- **WL.NVCh***; WL.C- **WL.VCh***	Sulfoquinovose degradation I	Pseudomonadota
PWY-7527	**B.C*** - B.NVCh; **B.C*** - B.VCh;	L-methionine salvage cycle III	Archaea, Bacteria, Metazoa
PWY-7528	**B.C*** - B.NVCh; **B.C*** - B.VCh; **B.V*** - B.VCh	L-methionine salvage cycle I	Archaea, Bacteria, Viridiplantae
PWY-7616	**NN.C*** - NN.V; **RIR.C*** - RIR.NVCh; **RIR.C*** - RIR.VCh; **RIR.C*** - RIR.V; **WL.C***- WL.NVCh; **WL.C***- WL.VCh	Methanol oxidation to carbon dioxide	Bacteria
PWY0-1277	**RIR.C*** - RIR.NVCh; **RIR.C*** - RIR.VCh	Aromatic-ring-cleavage, 3-phenylpropanoate and 3-(3-hydroxyphenyl)propanoate degradation	Pseudomonadota
PWY0-321	**B.C*** - B.NVCh; **B.C*** - B.VCh	Phenylacetate degradation I (aerobic)	Bacteria, Fungi
PWY1G-0	**NN.C*** - NN.V; **RIR.C*** - RIR.NVCh; **RIR.C*** - RIR.VCh;	Mycothiol biosynthesis	Actinomycetota

	**RIR.C*** - RIR.V; **WL.C***-WL.NVCh		
SUCSYN-PWY	**B.C*** - B.NVCh; **B.VCh*** - B.NVCh	Sucrose biosynthesis I	Cyanobacteriota, Viridiplantae
VALDEG-PWY	**B.C*** - B.NVCh; **B.C*** - B.VCh; **RIR.V*** -RIR.VCh	L-valine degradation I	Actinomycetota, Bacillota, Eukaryota, Pseudomonadota

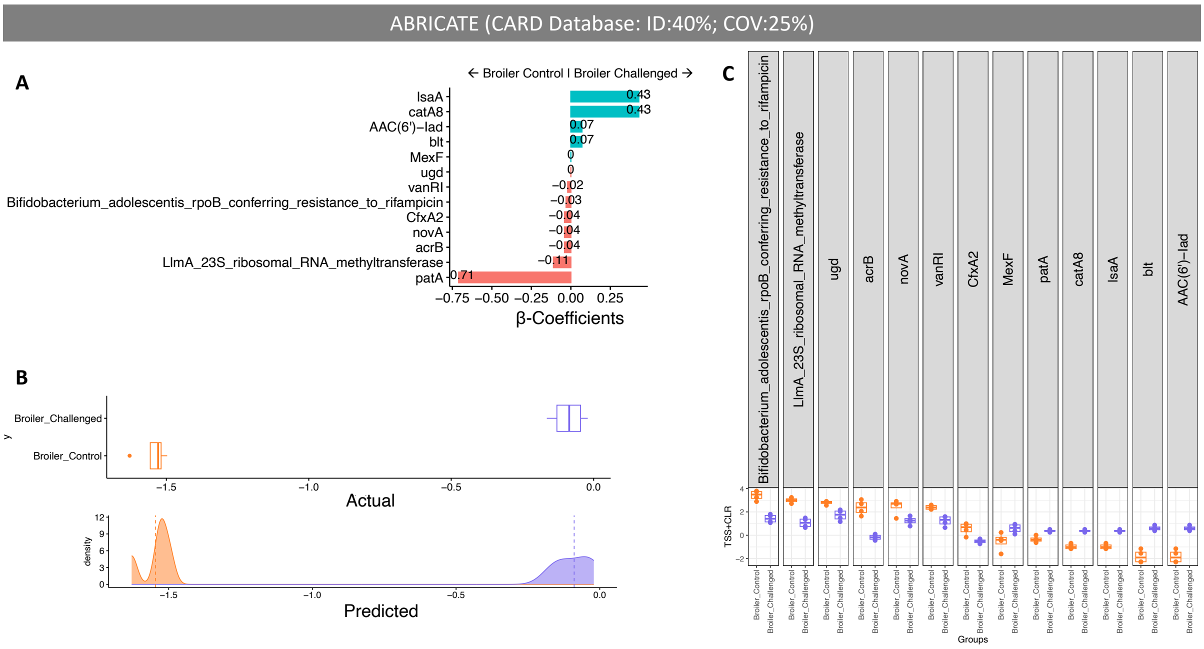

Supplementary Figure S17. Shotgun metagenomics differential analysis results for AMR genes found using ABRICATE (detected with at least 40% similarity and at least 25% coverage of sequences). **(A)** Non-zero β −coefficients returned from CODA-LASSO procedure as two disjoint sets (those that are associated with Broiler Challenged (positive; green) and those that are associated with Broiler Control (negative; red). **(B)** The density plot returned from the CODA-LASSO segregates the two groups and provides a graphical assessment of the classification accuracy (top: true; bottom: predicted from the procedure). **(C)** shows TSS+CLR (*Total Sum Scaling* followed by *Centralised Log Ratio*) normalised expression values of these features.

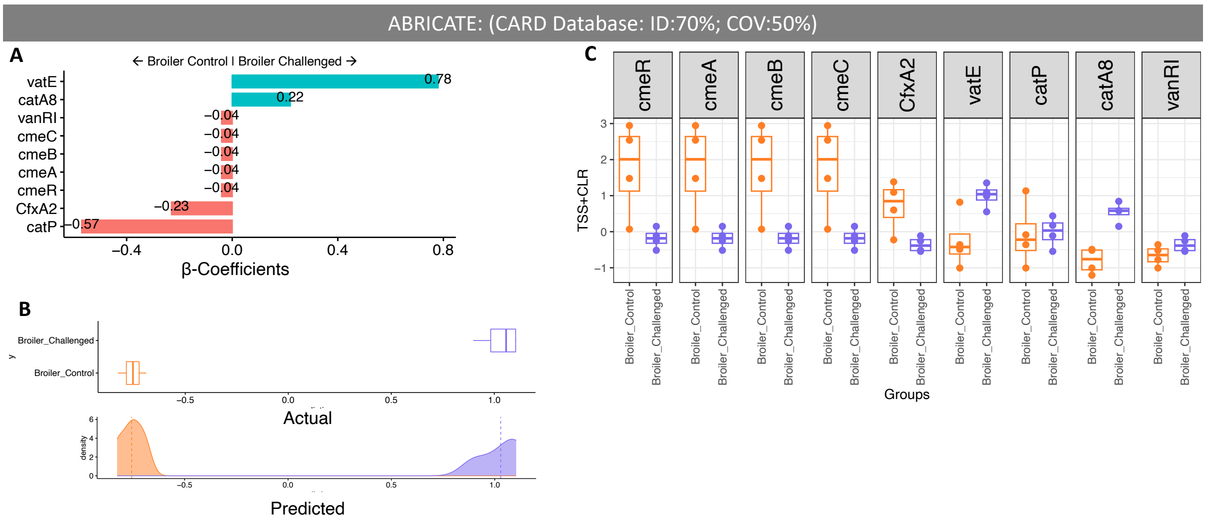

Supplementary Figure S18. Shotgun metagenomics differential analysis results for AMR genes found using ABRICATE (detected with at least 70% similarity and at least 50% coverage of sequences). **(A)** Non-zero β −coefficients returned from CODA-LASSO procedure as two disjoint sets (those that are associated with Broiler Challenged (positive; green) and those that are associated with Broiler Control (negative; red). **(B)** The density plot returned from the CODA-LASSO segregates the two groups and provides a graphical assessment of the classification accuracy (top: true; bottom: predicted from the procedure). **(C)** shows TSS+CLR (*Total Sum Scaling* followed by *Centralised Log Ratio*) normalised expression values of these features.

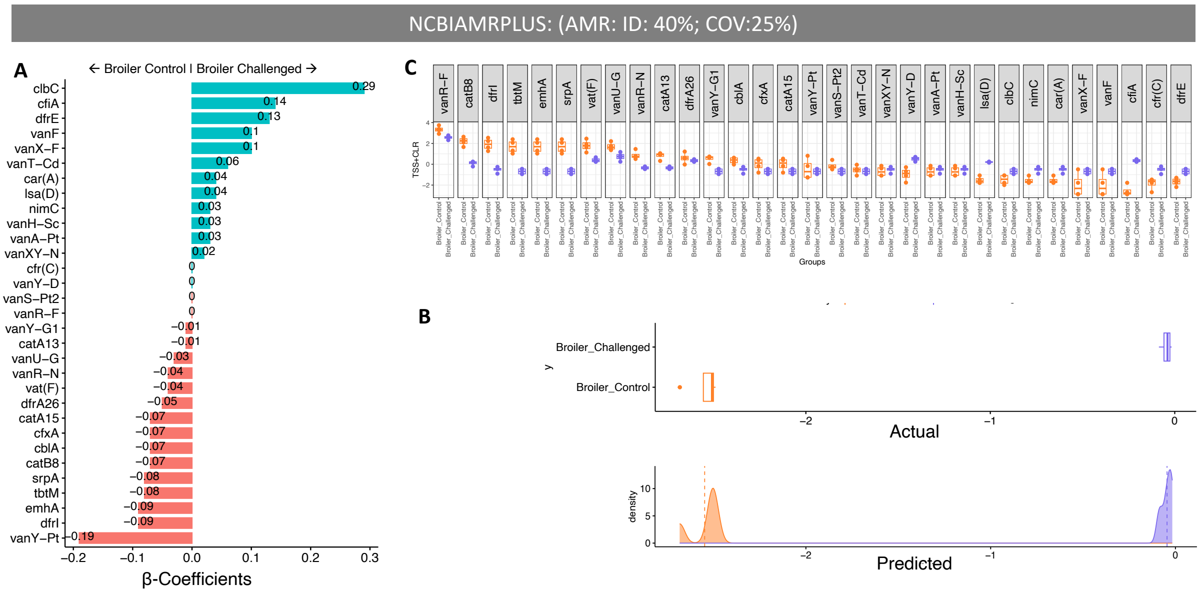

Supplementary Figure S19. Shotgun metagenomics differential analysis results for AMR genes found using NCBI AMRPLUS (detected with at least 40% similarity and at least 25% coverage of sequences). **(A)** Non-zero β −coefficients returned from CODA-LASSO procedure as two disjoint sets (those that are associated with Broiler Challenged (positive; green) and those that are associated with Broiler Control (negative; red). **(B)** The density plot returned from the CODA-LASSO segregates the two groups and provides a graphical assessment of the classification accuracy (top: true; bottom: predicted from the procedure). **(C)** shows TSS+CLR (*Total Sum Scaling* followed by *Centralised Log Ratio*) normalised expression values of these features.

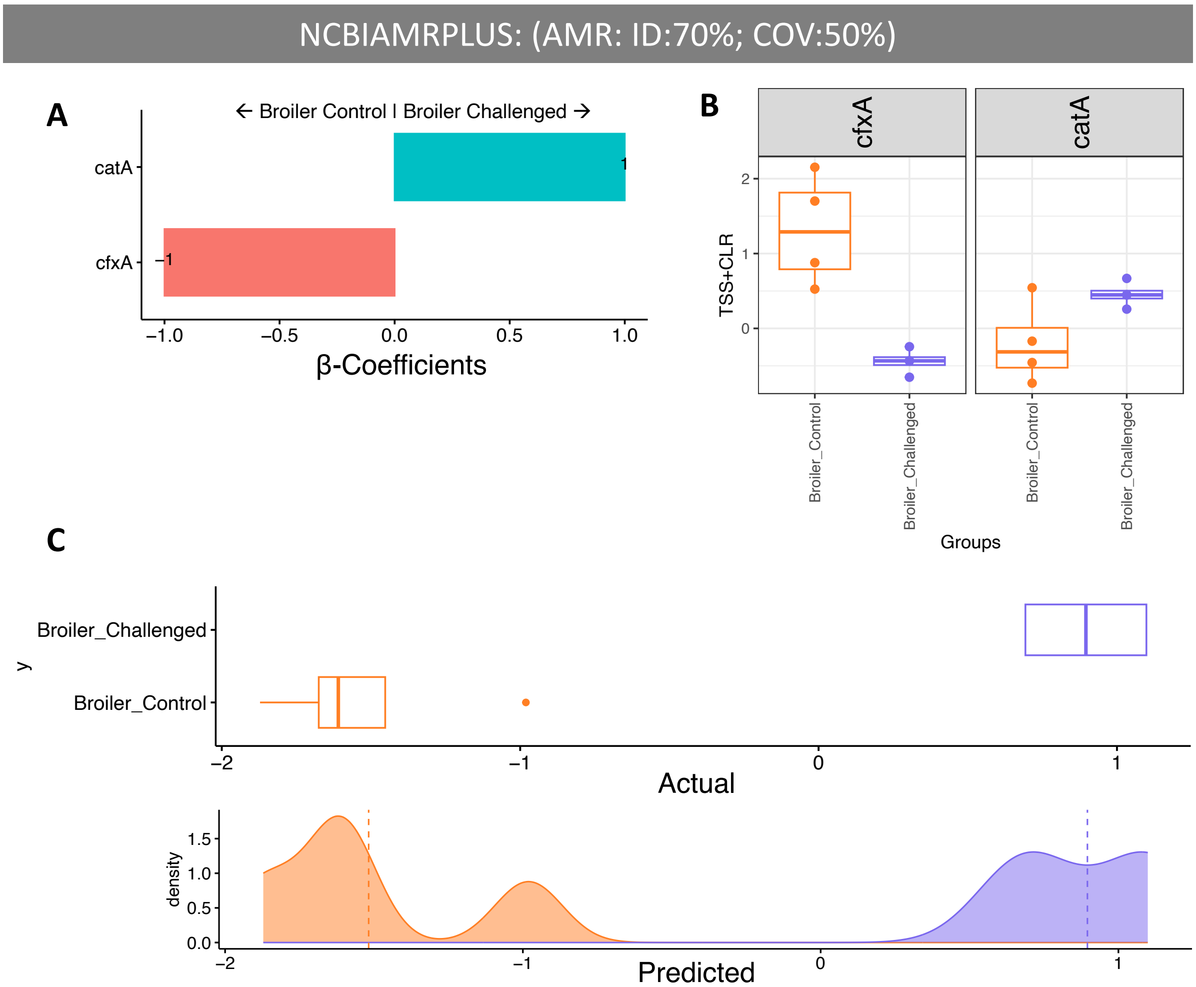

Supplementary Figure S20. Shotgun metagenomics differential analysis results for AMR genes found using NCBI AMRPLUS (detected with at least 70% similarity and at least 50% coverage of sequences). **(A)** Non-zero β −coefficients returned from CODA-LASSO procedure as two disjoint sets (those that are associated with Broiler Challenged (positive; green) and those that are associated with Broiler Control (negative; red). **(B)** The density plot returned from the CODA-LASSO segregates the two groups and provides a graphical assessment of the classification accuracy (top: true; bottom: predicted from the procedure). **(C)** shows TSS+CLR (*Total Sum Scaling* followed by *Centralised Log Ratio*) normalised expression values of these features.

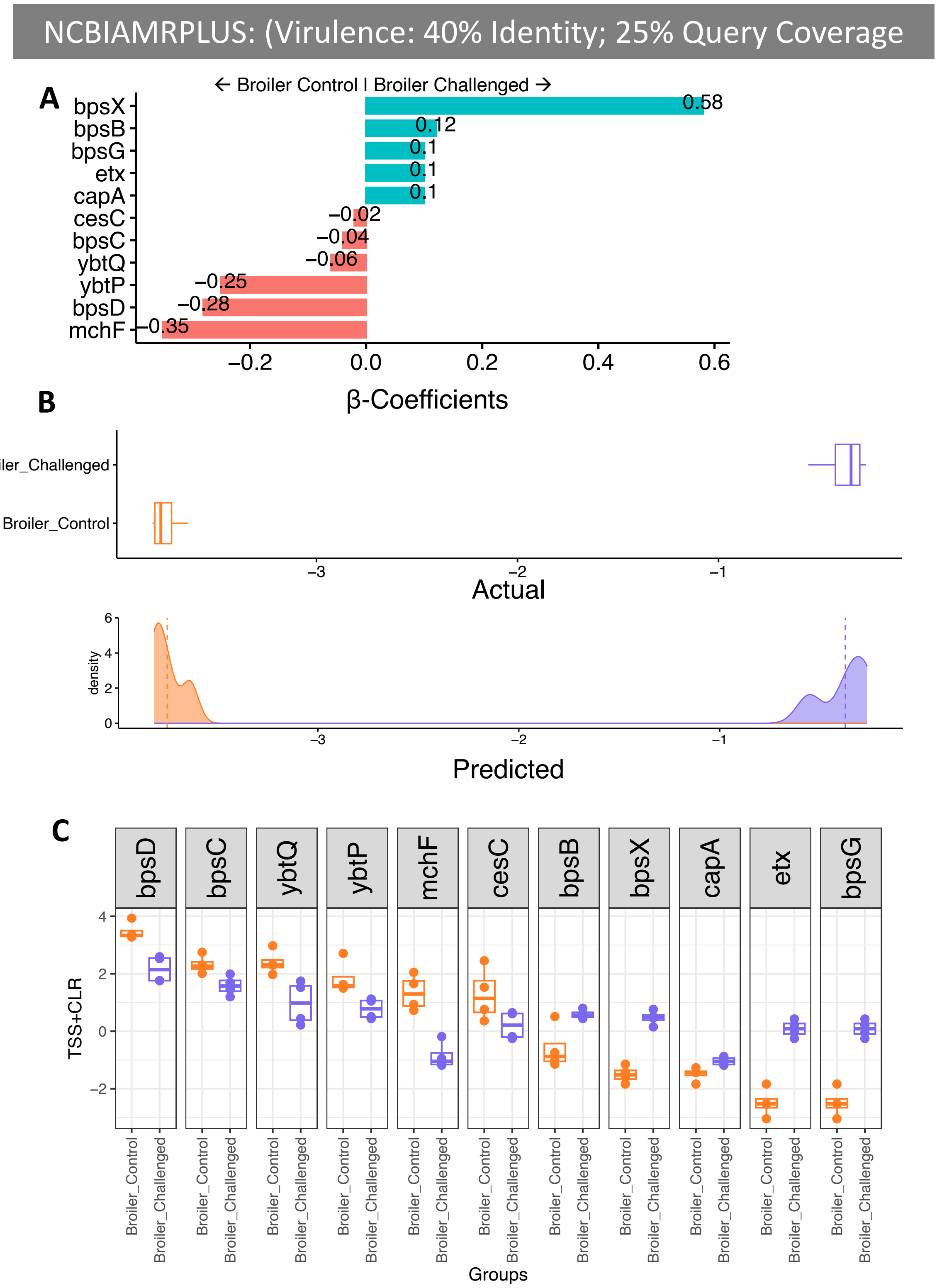

Supplementary Figure S21. Shotgun metagenomics differential analysis results for virulence genes found using NCBI AMRPLUS (detected with at least 40% similarity and at least 25% coverage of sequences). **(A)** Non-zero $\beta-$coefficients returned from CODA-LASSO procedure as two disjoint sets (those that are associated with Broiler Challenged (positive; green) and those that are associated with Broiler Control (negative; red). **(B)** The density plot returned from the CODA-LASSO segregates the two groups and provides a graphical assessment of the classification accuracy (top: true; bottom: predicted from the procedure).

(C) shows TSS+CLR (*Total Sum Scaling* followed by *Centralised Log Ratio*) normalised expression values of these features.

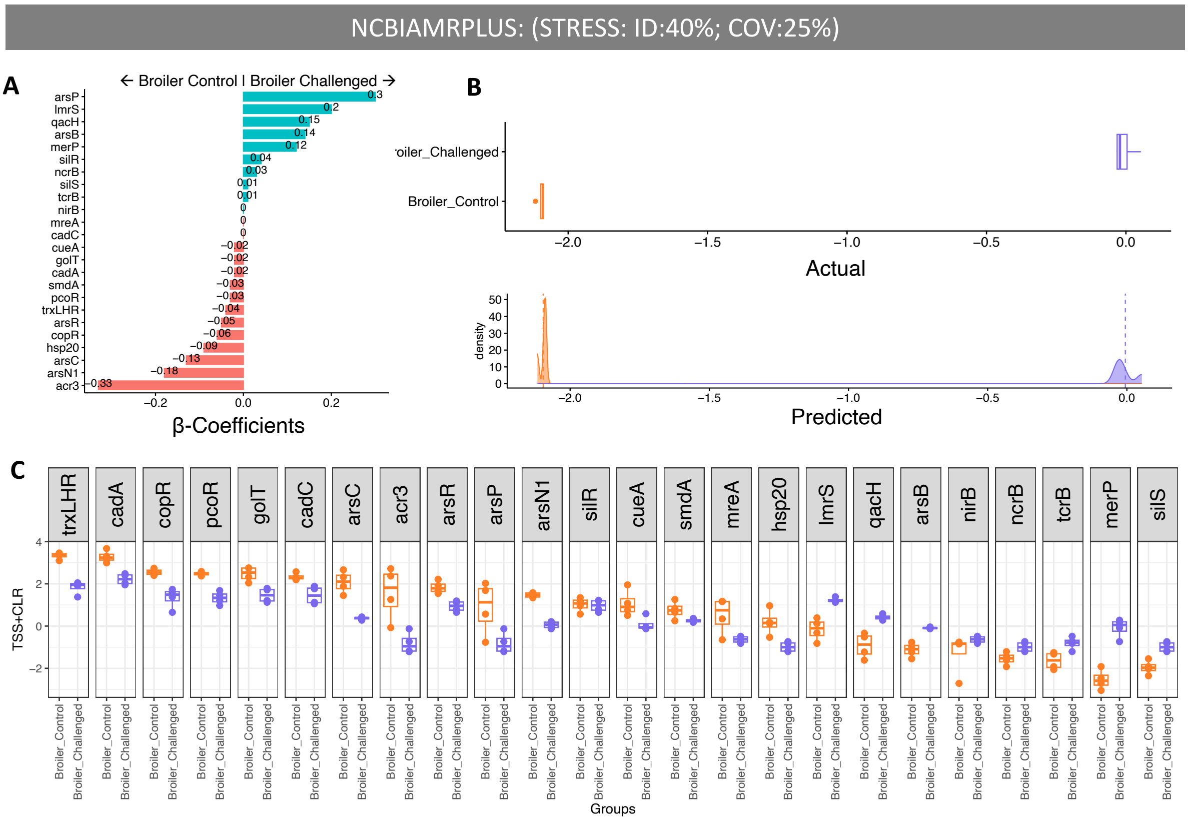

Supplementary Figure S22. Shotgun metagenomics differential analysis results for stress genes found using NCBI AMRPLUS (detected with at least 40% similarity and at least 25% coverage of sequences). **(A)** Non-zero β −coefficients returned from CODA-LASSO procedure as two disjoint sets (those that are associated with Broiler Challenged (positive; green) and those that are associated with Broiler Control (negative; red). **(B)** The density plot returned from the CODA-LASSO segregates the two groups and provides a graphical assessment of the classification accuracy (top: true; bottom: predicted from the procedure). **(C)** shows TSS+CLR (*Total Sum Scaling* followed by *Centralised Log Ratio*) normalised expression values of these features.

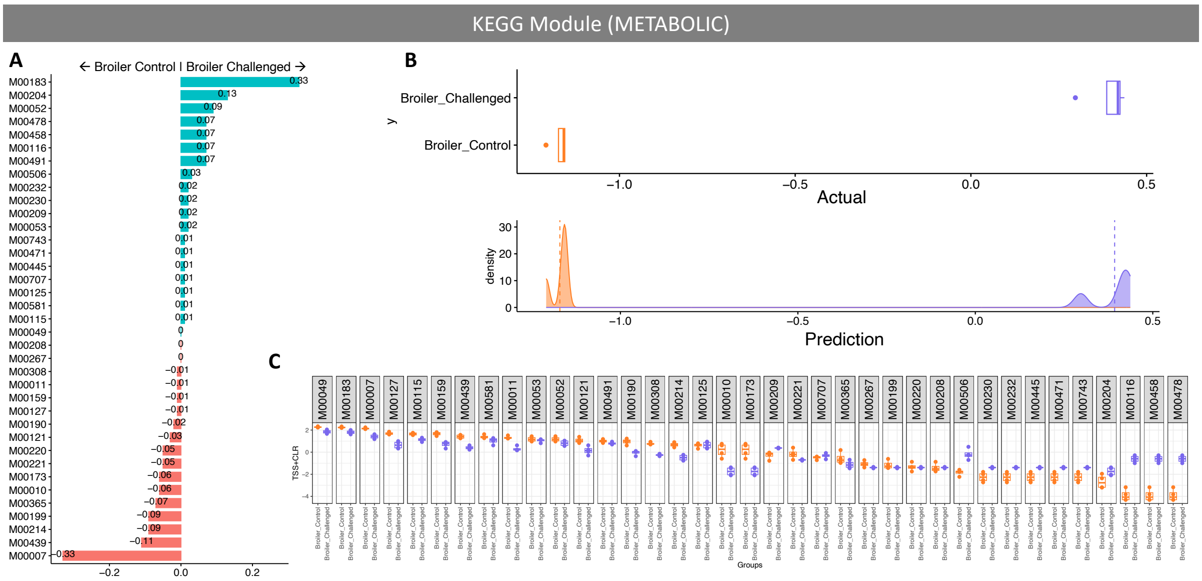

Supplementary Figure S23. Shotgun metagenomics differential analysis results for KEGG modules found using METABOLIC software. **(A)** Non-zero β −coefficients returned from CODA-LASSO procedure as two disjoint sets (those that are associated with Broiler Challenged (positive; green) and those that are associated with Broiler Control (negative; red). **(B)** The density plot returned from the CODA-LASSO segregates the two groups and provides a graphical assessment of the classification accuracy (top: true; bottom: predicted from the procedure). **(C)** shows TSS+CLR (*Total Sum Scaling* followed by *Centralised Log Ratio*) normalised expression values of these features. The details of KEGG modules including their names are given in Supplementary_Data_S3_METABOLIC_results.xlsx

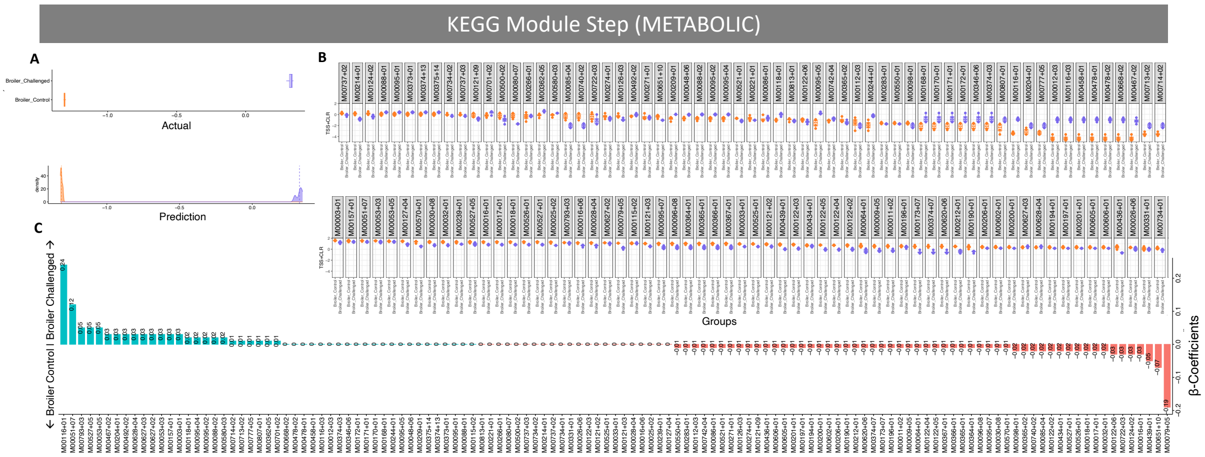

Supplementary Figure S24. Shotgun metagenomics differential analysis results for KEGG module steps found using METABOLIC software. **(A)** Non-zero β −coefficients returned from CODA-LASSO procedure as two disjoint sets (those that are associated with Broiler Challenged (positive; green) and those that are associated with Broiler Control (negative; red). **(B)** The density plot returned from the CODA-LASSO segregates the two groups and provides a graphical assessment of the classification accuracy (top: true; bottom: predicted from the procedure). **(C)** shows TSS+CLR (*Total Sum Scaling* followed by *Centralised Log Ratio*) normalised expression values of these features. The details of KEGG module steps including their names are given in Supplementary_Data_S3_METABOLIC_results.xlsx

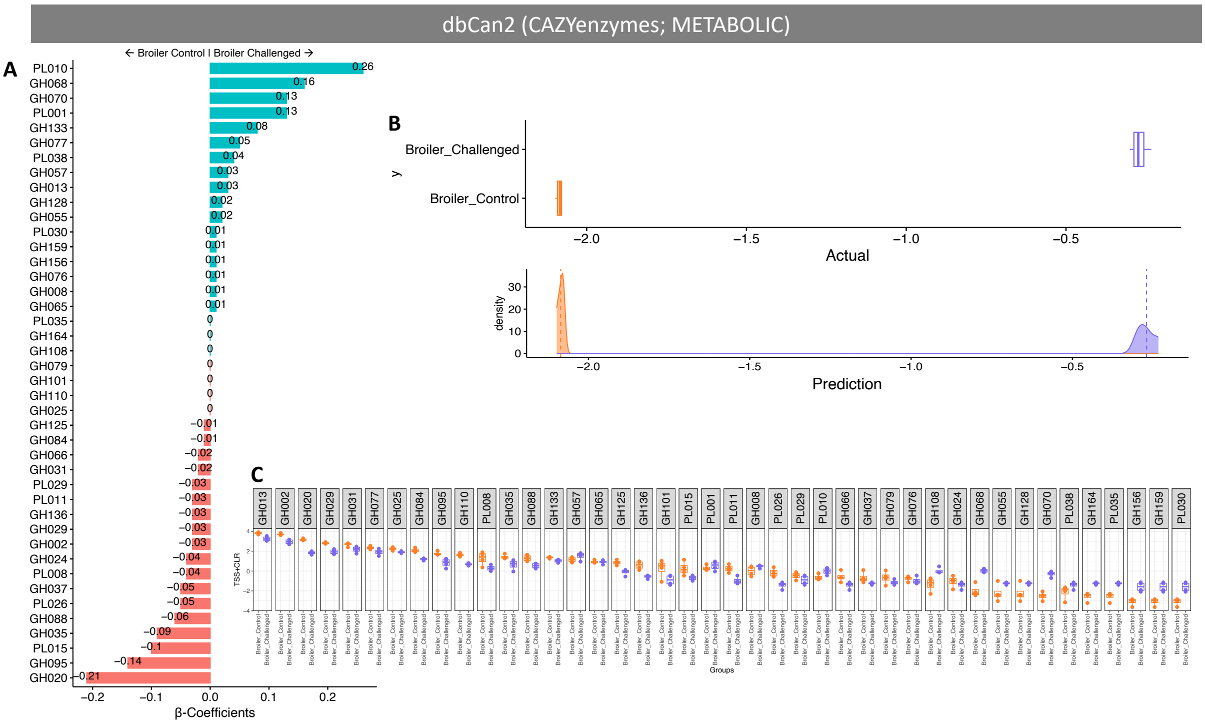

Supplementary Figure S25. Shotgun metagenomics differential analysis results for CAZymes found using METABOLIC software. **(A)** Non-zero β –coefficients returned from CODA-LASSO procedure as two disjoint sets (those that are associated with Broiler Challenged (positive; green) and those that are associated with Broiler Control (negative; red). **(B)** The density plot returned from the CODA-LASSO segregates the two groups and provides a graphical assessment of the classification accuracy (top: true; bottom: predicted from the procedure). **(C)** shows TSS+CLR (*Total Sum Scaling* followed by *Centralised Log Ratio*) normalised expression values of these features.

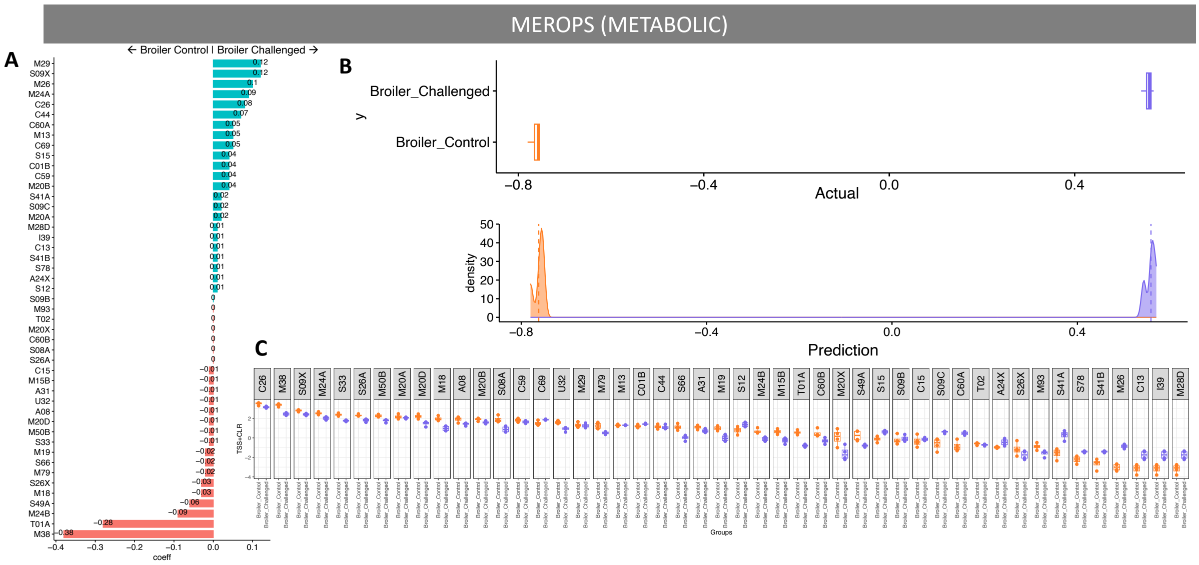

Supplementary Figure S26. Shotgun metagenomics differential analysis results for MEROPS peptidases found using METABOLIC software. **(A)** Non-zero β –coefficients returned from CODA-LASSO procedure as two disjoint sets (those that are associated with Broiler Challenged (positive; green) and those that are associated with Broiler Control (negative; red). **(B)** The density plot returned from the CODA-LASSO segregates the two groups and provides a graphical assessment of the classification accuracy (top: true; bottom:

predicted from the procedure). **(C)** shows TSS+CLR (*Total Sum Scaling* followed by *Centralised Log Ratio*) normalised expression values of these features.

References

Alauzet C, Aujoulat F, Lozniewski A, Brahim S Ben, Domenjod C, Enault C, Lavigne JP, Marchandin H. 2021. A new look at the genus solobacterium: A retrospective analysis of twenty-seven cases of infection involving s. moorei and a review of sequence databases and the literature. Microorganisms [Internet]. [accessed 2024 Jan 18] 9(6):1229. https://doi.org/10.3390/MICROORGANISMS9061229/S1

Ali A, Abbas S, Nawaz S, Man C, Liu YH, Li WJ, Ahmed I. 2023. Unraveling the draft genome and phylogenomic analysis of a multidrug-resistant Planococcus sp. NCCP-2050T: a promising novel bacteria from Pakistan. 3 Biotech [Internet]. [accessed 2024 Jan 18] 13(10):1–9. https://doi.org/10.1007/S13205-023-03748-Z/METRICS

Alneberg J, Bjarnason BS, De Bruijn I, Schirmer M, Quick J, Ijaz UZ, Lahti L, Loman NJ, Andersson AF, Quince C. 2014. Binning metagenomic contigs by coverage and composition. Nat Methods 2014 1111 [Internet]. [accessed 2023 Dec 25] 11(11):1144–1146. https://doi.org/10.1038/nmeth.3103

Alvarenga BO, Paiva JB, Souza AIS, Rodrigues DR, Tizioto PC, Ferreira AJP. 2023. Metagenomics analysis of the morphological aspects and bacterial composition of broiler feces. Poult Sci. 102(2):102401. https://doi.org/10.1016/J.PSJ.2022.102401

Ameer A, Cheng Y, Saleem F, Uzma, McKenna A, Richmond A, Gundogdu O, Sloan WT, Javed S, Ijaz UZ. 2023. Temporal stability and community assembly mechanisms in healthy broiler cecum. Front Microbiol [Internet]. [accessed 2024 Jan 17] 14. https://doi.org/10.3389/FMICB.2023.1197838/FULL

Amoozegar MA, Bagheri M, Makhdoumi-Kakhki A, Didari M, Schumann P, Nikou MM, Sánchez-Porro C, Ventosa A. 2014. Aliicoccus persicus gen. nov., sp. nov., a halophilic member of the Firmicutes isolated from a hypersaline lake. Int J Syst Evol Microbiol [Internet]. [accessed 2024 Jan 18] 64(PART 6):1964–1969. https://doi.org/10.1099/IJS.0.058545-0/CITE/REFWORKS

Anang DM, Rusul G, Radu S, Bakar J, Beuchat LR. 2006. Inhibitory Effect of Oxalic Acid on Bacterial Spoilage of Raw Chilled Chicken. J Food Prot. 69(8):1913–1919. https://doi.org/10.4315/0362-028X-69.8.1913

Anantharaman K, Brown CT, Hug LA, Sharon I, Castelle CJ, Probst AJ, Thomas BC, Singh A, Wilkins MJ, Karaoz U, et al. 2016. Thousands of microbial genomes shed light on interconnected biogeochemical processes in an aquifer system. Nat Commun 2016 71 [Internet]. [accessed 2023 Dec 25] 7(1):1–11. https://doi.org/10.1038/ncomms13219

Andrade BGN, Bressani FA, Cuadrat RRC, Cardoso TF, Malheiros JM, de Oliveira PSN, Petrini J, Mourão GB, Coutinho LL, Reecy JM, et al. 2022. Stool and Ruminal Microbiome Components Associated With Methane Emission and Feed Efficiency in Nelore Beef Cattle. Front Genet [Internet]. [accessed 2024 Mar 13] 13:812828. https://doi.org/10.3389/FGENE.2022.812828/BIBTEX

Artdita CA, Zhuang YR, Liu TY, Cheng CY, Hsiao FSH, Lin YY. 2021. The effect of feeding restriction on the microbiota and metabolome response in late-phase laying hens. Animals [Internet]. [accessed 2024 Mar 13] 11(11):3043. https://doi.org/10.3390/ANI11113043/S1

Baqi M, Mazzulli T. 1996. Oligella Infections: Case Report and Review of the Literature. Can J Infect Dis Med Microbiol [Internet]. [accessed 2024 Mar 13] 7(6):377–379. https://doi.org/10.1155/1996/153512

Becker K, Rutsch F, Uekötter A, Kipp F, König J, Marquardt T, Peters G, Von Eiff C. 2008. Kocuria rhizophila adds to the emerging spectrum of micrococcal species involved in human infections. J Clin Microbiol [Internet]. [accessed 2024 Jan 18] 46(10):3537–3539. https://doi.org/10.1128/JCM.00823-08/ASSET/10B67097-BF10-4798-9724-0072D03E83DB/ASSETS/GRAPHIC/ZJM0100883940001.JPEG

Biddle A, Stewart L, Blanchard J, Leschine S. 2013. Untangling the Genetic Basis of Fibrolytic Specialization by Lachnospiraceae and Ruminococcaceae in Diverse Gut

Communities. Divers 2013, Vol 5, Pages 627-640 [Internet]. [accessed 2024 Jan 18] 5(3):627–640. https://doi.org/10.3390/D5030627
Bolton FJ, Wareing DRA, Sails AD. 1997. Comparison of a novel microaerobic system with three other gas-generating systems for the recovery of Campylobacter species from human faecal samples. Eur J Clin Microbiol Infect Dis [Internet]. [accessed 2024 Jan 18] 16(11):839–842. https://doi.org/10.1007/BF01700415/METRICS
Bolyen E, Rideout JR, Dillon MR, Bokulich NA, Abnet CC, Al-Ghalith GA, Alexander H, Alm EJ, Arumugam M, Asnicar F, et al. 2019. Reproducible, interactive, scalable and extensible microbiome data science using QIIME 2. Nat Biotechnol 2019 378 [Internet]. [accessed 2023 Oct 2] 37(8):852–857. https://doi.org/10.1038/s41587-019-0209-9
Borey M, Bed'Hom B, Bruneau N, Estellé J, Larsen F. 2021. Caecal Microbiota Composition of Experimental Laying Hens Differs According to Genetic Line and Vaccination to IBV [Internet]. https://www.researchsquare.com/article/rs-177864/latest.pdf
Campos PM, Miska KB, Jenkins MC, Yan X, Proszkowiec-Weglarz M. 2023. Effects of Eimeria acervulina infection on the luminal and mucosal microbiota of the duodenum and jejunum in broiler chickens. Front Microbiol [Internet]. [accessed 2024 Mar 13] 14:1147579. https://doi.org/10.3389/FMICB.2023.1147579/BIBTEX
Chaumeil PA, Mussig AJ, Hugenholtz P, Parks DH. 2020. GTDB-Tk: a toolkit to classify genomes with the Genome Taxonomy Database. Bioinformatics [Internet]. [accessed 2023 Dec 25] 36(6):1925–1927. https://doi.org/10.1093/BIOINFORMATICS/BTZ848
Clavijo V, Morales T, Vives-Flores MJ, Reyes Muñoz A. 2022. The gut microbiota of chickens in a commercial farm treated with a Salmonella phage cocktail. Sci Reports 2022 121 [Internet]. [accessed 2024 Jan 18] 12(1):1–16. https://doi.org/10.1038/s41598-021-04679-6
Conradie TA, Pieterse E, Jacobs K. 2018. Application of Paracoccus marcusii as a potential feed additive for laying hens. Poult Sci. 97(3):986–994. https://doi.org/10.3382/PS/PEX377
Crhanova M, Karasova D, Juricova H, Matiasovicova J, Jahodarova E, Kubasova T, Seidlerova Z, Cizek A, Rychlik I. 2019. Systematic Culturomics Shows that Half of Chicken Caecal Microbiota Members can be Grown in Vitro Except for Two Lineages of Clostridiales and a Single Lineage of Bacteroidetes. Microorg 2019, Vol 7, Page 496 [Internet]. [accessed 2024 Jan 18] 7(11):496. https://doi.org/10.3390/MICROORGANISMS7110496
Crispo M, Shivaprasad HL, Cooper GL, Bickford AA, Stoute ST. 2018. Streptococcosis in Commercial and Noncommercial Avian Species in California: 95 Cases (2000–2017). Avian Dis [Internet]. [accessed 2024 Jan 18] 62(2):152–162. https://doi.org/10.1637/11765-103117-REG.1
Derrien M, Belzer C, de Vos WM. 2017. Akkermansia muciniphila and its role in regulating host functions. Microb Pathog. 106:171–181. https://doi.org/10.1016/J.MICPATH.2016.02.005
Detilleux J, Moula N, Dawans E, Taminiau B, Daube G, Leroy P. 2022. A Probabilistic Structural Equation Model to Evaluate Links between Gut Microbiota and Body Weights of Chicken Fed or Not Fed Insect Larvae. Biology (Basel) [Internet]. [accessed 2024 Mar 13] 11(3):357. https://doi.org/10.3390/BIOLOGY11030357/S1
Ding J, Zhou H, Luo L, Xiao L, Yang K, Yang L, Zheng Y, Xu K, He C, Han C, et al. 2021. Heritable Gut Microbiome Associated with Salmonella enterica Serovar Pullorum Infection in Chickens . mSystems [Internet]. [accessed 2024 Mar 13] 6(1). https://doi.org/10.1128/MSYSTEMS.01192-20/SUPPL_FILE/MSYSTEMS.01192-20-ST006.XLSX
Dong XB, Li X, Zhang CH, Wang JZ, Tang CH, Sun HM, Jia W, Li Y, Chen LL. 2014. Development of a novel method for hot-pressure extraction of protein from chicken bone and the effect of enzymatic hydrolysis on the extracts. Food Chem. 157:339–346. https://doi.org/10.1016/J.FOODCHEM.2014.02.043
Douglas GM, Maffei VJ, Zaneveld JR, Yurgel SN, Brown JR, Taylor CM, Huttenhower C, Langille MGI. 2020. PICRUSt2 for prediction of metagenome functions. Nat Biotechnol 2020 386 [Internet]. [accessed 2022 Dec 12] 38(6):685–688. https://doi.org/10.1038/s41587-020-0548-6

El-Sharkawy H, Tahoun A, Rizk AM, Suzuki T, Elmonir W, Nassef E, Shukry M, Germoush MO, Farrag F, Bin-Jumah M, Mahmoud AM. 2020. Evaluation of Bifidobacteria and Lactobacillus Probiotics as Alternative Therapy for Salmonella typhimurium Infection in Broiler Chickens. Anim 2020, Vol 10, Page 1023 [Internet]. [accessed 2024 Jan 18] 10(6):1023. https://doi.org/10.3390/ANI10061023
Enurah LU. 2016. An Outbreak of Corynebacterium Diphtheriae. 16(1):1–4.
Ezaki T. 2015. Gallicola. Bergey's Man Syst Archaea Bact [Internet]. [accessed 2024 Mar 13]:1–3. https://doi.org/10.1002/9781118960608.GBM00714
Farkas V, Csitári G, Menyhárt L, Such N, Pál L, Husvéth F, Rawash MA, Mezőlaki Á, Dublecz K. 2022. Microbiota Composition of Mucosa and Interactions between the Microbes of the Different Gut Segments Could Be a Factor to Modulate the Growth Rate of Broiler Chickens. Animals. 12(10):1296. https://doi.org/10.3390/ANI12101296/S1
Farooq M, Smoglica C, Ruffini F, Soldati L, Marsilio F, Di Francesco CE. 2022. Antibiotic Resistance Genes Occurrence in Conventional and Antibiotic-Free Poultry Farming, Italy. Anim an Open Access J from MDPI [Internet]. [accessed 2023 Dec 18] 12(18):2310. https://doi.org/10.3390/ANI12182310
Feng Y, Zhang M, Liu Y, Yang X, Wei F, Jin X, Liu D, Guo Y, Hu Y. 2023. Quantitative microbiome profiling reveals the developmental trajectory of the chicken gut microbiota and its connection to host metabolism. iMeta [Internet]. [accessed 2024 Jan 18] 2(2):e105. https://doi.org/10.1002/IMT2.105
Finn RD, Bateman A, Clements J, Coggill P, Eberhardt RY, Eddy SR, Heger A, Hetherington K, Holm L, Mistry J, et al. 2014. Pfam: the protein families database. Nucleic Acids Res [Internet]. [accessed 2023 Dec 25] 42(D1):D222–D230. https://doi.org/10.1093/NAR/GKT1223
Frias H, Murga Valderrama NL, Flores Durand GJ, Cornejo VG, Romani AC, Bardales W, Segura GT, Polveiro RC, Vieira D da S, Ramos Sanchez EM, et al. 2023. Comparative analysis of fasting effects on the cecum microbiome in three guinea pig breeds: Andina, Inti, and Peru. Front Microbiol. 14:1283738. https://doi.org/10.3389/FMICB.2023.1283738/BIBTEX
Gilroy R, Ravi A, Getino M, Pursley I, Horton DL, Alikhan NF, Baker D, Gharbi K, Hall N, Watson M, et al. 2021. Extensive microbial diversity within the chicken gut microbiome revealed by metagenomics and culture. PeerJ [Internet]. [accessed 2024 Jan 18] 9:e10941. https://doi.org/10.7717/PEERJ.10941/SUPP-2
Gong L, Wang B, Zhou Y, Tang L, Zeng Z, Zhang H, Li W. 2021. Protective Effects of Lactobacillus plantarum 16 and Paenibacillus polymyxa 10 Against Clostridium perfringens Infection in Broilers. Front Immunol. 11:628374. https://doi.org/10.3389/FIMMU.2020.628374/BIBTEX
Goris T, Cuadrat RRC, Braune A. 2021. Flavonoid-modifying capabilities of the human gut microbiome—an in silico study. Nutrients [Internet]. [accessed 2024 Jan 18] 13(8):2688. https://doi.org/10.3390/NU13082688/S1
Guerrant RL, Walker DH, Weller PF. 2006. Tropical Infectious Diseases. Trop Infect Dis Princ Pathog Pract 2-Volume Set with CD-ROM [Internet]. [accessed 2024 Mar 13]:1–1786. https://doi.org/10.1016/B978-0-443-06668-9.X5001-7
Haffajee AD, Teles RP, Socransky SS. 2006. Association of Eubacterium nodatum and Treponema denticola with human periodontitis lesions. Oral Microbiol Immunol [Internet]. [accessed 2024 Mar 13] 21(5):269–282. https://doi.org/10.1111/J.1399-302X.2006.00287.X
Hao Y, Ji Z, Shen Z, Xue Y, Zhang B, Yu D, Liu T, Luo D, Xing G, Tang J, et al. 2022. Increase Dietary Fiber Intake Ameliorates Cecal Morphology and Drives Cecal Species-Specific of Short-Chain Fatty Acids in White Pekin Ducks. Front Microbiol. 13:853797. https://doi.org/10.3389/FMICB.2022.853797/BIBTEX
Hazeleger WC, Wouters JA, Rombouts FM, Abee T. 1998. Physiological activity of Campylobacter jejuni far below the minimal growth temperature. Appl Environ Microbiol [Internet]. [accessed 2024 Jan 18] 64(10):3917–3922. https://doi.org/10.1128/AEM.64.10.3917-3922.1998/ASSET/A883226D-A031-4705-B078-9996D81596E9/ASSETS/GRAPHIC/AM1080846006.JPEG

Heijne M, van der Goot J, Buys H, Dinkla A, Roest HJ, van Keulen L, Koets A. 2021. Experimental Chlamydia gallinacea infection in chickens does not protect against a subsequent experimental Chlamydia psittaci infection. Vet Res [Internet]. [accessed 2024 Mar 13] 52(1):141. https://doi.org/10.1186/S13567-021-01011-Y/FIGURES/6
Hill JE, Kelley LC, Langheinrich KA. 1992. Visceral granulomas in chickens infected with a filamentous bacteria. Avian Dis. 36(1):172–176. https://doi.org/10.2307/1591735
Hug LA, Baker BJ, Anantharaman K, Brown CT, Probst AJ, Castelle CJ, Butterfield CN, Hernsdorf AW, Amano Y, Ise K, et al. 2016. A new view of the tree of life. Nat Microbiol 2016 15 [Internet]. [accessed 2023 Dec 25] 1(5):1–6. https://doi.org/10.1038/nmicrobiol.2016.48
Ijaz UZ, Sivaloganathan L, McKenna A, Richmond A, Kelly C, Linton M, Stratakos AC, Lavery U, Elmi A, Wren BW, et al. 2018. Comprehensive Longitudinal Microbiome Analysis of the Chicken Cecum Reveals a Shift From Competitive to Environmental Drivers and a Window of Opportunity for Campylobacter. Front Microbiol. 9(OCT). https://doi.org/10.3389/FMICB.2018.02452
Jin L, Jin CZ, Lee HG, Lee CS. 2021. Genomic insights into denitrifying methane-oxidizing bacteria gemmobacter fulva sp. Nov., isolated from an anabaena culture. Microorganisms [Internet]. [accessed 2024 Mar 13] 9(12):2423. https://doi.org/10.3390/MICROORGANISMS9122423/S1
Jochum JM, Redweik GAJ, Ott LC, Mellata M. 2021. Bacteria Broadly-Resistant to Last Resort Antibiotics Detected in Commercial Chicken Farms. Microorg 2021, Vol 9, Page 141 [Internet]. [accessed 2024 Mar 13] 9(1):141. https://doi.org/10.3390/MICROORGANISMS9010141
Joshi N. and FJ. 2011. : A Sliding-Window, Adaptive, Quality-Based Trimming Tool for Fastq Files (Version 1.33). 1.33 ed. San Francisco, CA. . Sickle.
Jung A, Chen LR, Suyemoto MM, Barnes HJ, Borst LB. 2018. A Review of Enterococcus cecorum Infection in Poultry. Avian Dis [Internet]. [accessed 2024 Jan 18] 62(3):261–271. https://doi.org/10.1637/11825-030618-REVIEW.1
Kageyama A, Benno Y. 2000. Phylogenic and phenotypic characterization of some Eubacterium-like isolates from human feces: Description of Solobacterium moorei gen. nov., sp. nov. Microbiol Immunol [Internet]. [accessed 2024 Jan 18] 44(4):223–227. https://doi.org/10.1111/j.1348-0421.2000.tb02487.x
Kämpfer P, Poppel MT, Wilharm G, Glaeser SP, Busse HJ. 2014. Paenochrobactrum pullorum sp. nov. isolated from a chicken. Int J Syst Evol Microbiol [Internet]. [accessed 2024 Mar 13] 64(PART 5):1724–1728. https://doi.org/10.1099/IJS.0.061101-0/CITE/REFWORKS
Kanehisa M, Goto S. 2000. KEGG: Kyoto Encyclopedia of Genes and Genomes. Nucleic Acids Res [Internet]. [accessed 2023 Dec 25] 28(1):27–30. https://doi.org/10.1093/NAR/28.1.27
Kang DD, Li F, Kirton E, Thomas A, Egan R, An H, Wang Z. 2019. MetaBAT 2: An adaptive binning algorithm for robust and efficient genome reconstruction from metagenome assemblies. PeerJ [Internet]. [accessed 2023 Dec 25] 2019(7):e7359. https://doi.org/10.7717/PEERJ.7359/SUPP-3
Kempf F, Menanteau P, Rychlik I, Kubasová T, Trotereau J, Virlogeux-Payant I, Schaeffer S, Schouler C, Drumo R, Guitton E, Velge P. 2020. Gut microbiota composition before infection determines the Salmonella super- and low-shedder phenotypes in chicken. Microb Biotechnol [Internet]. [accessed 2022 Nov 21] 13(5):1611. https://doi.org/10.1111/1751-7915.13621
Khan S, Chousalkar KK. 2020. Salmonella Typhimurium infection disrupts but continuous feeding of Bacillus based probiotic restores gut microbiota in infected hens. J Anim Sci Biotechnol [Internet]. [accessed 2024 Jan 18] 11(1):1–16. https://doi.org/10.1186/S40104-020-0433-7/FIGURES/8
Kim BC, Jeon BS, Kim S, Kim H, Um Y, Sang BI. 2015. Caproiciproducens galactitolivorans gen. Nov., sp. nov., a bacterium capable of producing caproic acid from galactitol, isolated from a wastewater treatment plant. Int J Syst Evol Microbiol [Internet]. [accessed 2024 Jan 18] 65(12):4902–4908. https://doi.org/10.1099/IJSEM.0.000665/CITE/REFWORKS

Kläring K, Hanske L, Bui N, Charrier C, Blaut M, Haller D, Plugge CM, Clavel T. 2013. Intestinimonas butyriciproducens gen. nov., sp. nov., a butyrate-producing bacterium from the mouse intestine. Int J Syst Evol Microbiol [Internet]. [accessed 2022 Nov 21] 63(Pt 12):4606–4612. https://doi.org/10.1099/IJS.0.051441-0
Kollarcikova M, Kubasova T, Karasova D, Crhanova M, Cejkova D, Sisak F, Rychlik I. 2019. Use of 16S rRNA gene sequencing for prediction of new opportunistic pathogens in chicken ileal and cecal microbiota. Poult Sci. 98(6):2347–2353. https://doi.org/10.3382/PS/PEY594
Kubasova T, Davidova-Gerzova L, Babak V, Cejkova D, Montagne L, Le-Floc'h N, Rychlik I. 2018. Effects of host genetics and environmental conditions on fecal microbiota composition of pigs. PLoS One [Internet]. [accessed 2024 Jan 18] 13(8):e0201901. https://doi.org/10.1371/JOURNAL.PONE.0201901
Kubasova T, Faldynova M, Crhanova M, Karasova D, Zeman M, Babak V, Rychlik I. 2022. Succession, Replacement, and Modification of Chicken Litter Microbiota. Appl Environ Microbiol [Internet]. [accessed 2024 Jan 18] 88(24). https://doi.org/10.1128/AEM.01809-22/SUPPL_FILE/AEM.01809-22-S0001.XLSX
LaClaire L, Facklam R. 2000. Antimicrobial Susceptibilities and Clinical Sources of Facklamia Species. Antimicrob Agents Chemother [Internet]. [accessed 2024 Jan 18] 44(8):2130–2132. https://doi.org/10.1128/AAC.44.8.2130-2132.2000
Lee MD. 2019. GToTree: a user-friendly workflow for phylogenomics. Bioinformatics [Internet]. [accessed 2024 Jul 3] 35(20):4162–4164. https://doi.org/10.1093/BIOINFORMATICS/BTZ188
Lena M, Syahramadani DF, Gustya AN, Darmawan A, Sumiati, Winarsih W, Maeda M, Wiryawan KG. 2022. The Influence of Lactococcus and Bacillus species Probiotics on Performance, Energy Utilization, Intestinal Ecosystem of Broiler Chickens. Adv Anim Vet Sci. 10(3):651–658. https://doi.org/10.17582/JOURNAL.AAVS/2022/10.3.651.658
Leth ML, Ejby M, Workman C, Ewald DA, Pedersen SS, Sternberg C, Bahl MI, Licht TR, Aachmann FL, Westereng B, Hachem MA. 2018. Differential bacterial capture and transport preferences facilitate co-growth on dietary xylan in the human gut. Nat Microbiol 2018 35 [Internet]. [accessed 2024 Jan 18] 3(5):570–580. https://doi.org/10.1038/s41564-018-0132-8
Li A, Wang Y, Hao J, Wang L, Quan L, Duan K, Fakhar-e-Alam Kulyar M, Ullah K, Zhang J, Wu Y, Li K. 2022. Long-term hexavalent chromium exposure disturbs the gut microbial homeostasis of chickens. Ecotoxicol Environ Saf. 237:113532. https://doi.org/10.1016/J.ECOENV.2022.113532
Li D, Liu CM, Luo R, Sadakane K, Lam TW. 2015. MEGAHIT: An ultra-fast single-node solution for large and complex metagenomics assembly via succinct de Bruijn graph. Bioinformatics. 31(10):1674–1676. https://doi.org/10.1093/bioinformatics/btv033
Li MH, Meng JX, Wang W, He M, Zhao ZY, Ma N, Lv QB, Qin YF, Geng HL, Zhao Q, et al. 2022. Dynamic description of temporal changes of gut microbiota in broilers. Poult Sci. 101(9):102037. https://doi.org/10.1016/J.PSJ.2022.102037
Li Y, Guo B, Wu Z, Wang W, Li C, Liu G, Cai H. 2020. Effects of Fermented Soybean Meal Supplementation on the Growth Performance and Cecal Microbiota Community of Broiler Chickens. Anim 2020, Vol 10, Page 1098 [Internet]. [accessed 2024 Jan 18] 10(6):1098. https://doi.org/10.3390/ANI10061098
Li Y, Zhang LL, Liu L, Tian YQ, Liu XF, Li WJ, Dai YM. 2017. Paludicola psychrotolerans gen. Nov., sp. nov., a novel psychrotolerant chitinolytic anaerobe of the family ruminococcaceae. Int J Syst Evol Microbiol [Internet]. [accessed 2024 Mar 13] 67(10):4100–4103. https://doi.org/10.1099/IJSEM.0.002260/CITE/REFWORKS
Li Z, Wang W, Liu D, Guo Y. 2018. Effects of Lactobacillus acidophilus on the growth performance and intestinal health of broilers challenged with Clostridium perfringens. J Anim Sci Biotechnol [Internet]. [accessed 2022 Nov 21] 9(1). https://doi.org/10.1186/S40104-018-0243-3
Liu DY, Xiao X, Wang HH, Zhang QY, Zou YF. 2019. Characterization of the bacterial community of braised chicken, a specialty poultry product in China. Poult Sci. 98(2):1055–1063. https://doi.org/10.3382/PS/PEY375
Liu J, Robinson K, Lyu W, Yang Q, Wang J, Christensen KD, Zhang G. 2023.

Anaerobutyricum and Subdoligranulum Are Differentially Enriched in Broilers with Disparate Weight Gains. Anim 2023, Vol 13, Page 1834 [Internet]. [accessed 2024 Jan 18] 13(11):1834. https://doi.org/10.3390/ANI13111834
Liu J, Stewart SN, Robinson K, Yang Q, Lyu W, Whitmore MA, Zhang G. 2021. Linkage between the intestinal microbiota and residual feed intake in broiler chickens. J Anim Sci Biotechnol [Internet]. [accessed 2022 Nov 21] 12(1). https://doi.org/10.1186/S40104-020-00542-2
Liu X, Mao B, Gu J, Wu J, Cui S, Wang G, Zhao J, Zhang H, Chen W. 2021. Blautia—a new functional genus with potential probiotic properties? Gut Microbes [Internet]. [accessed 2024 Jan 18] 13(1):1–21. https://doi.org/10.1080/19490976.2021.1875796
Luo Y, Chen H, Yu B, He J, Zheng P, Mao X, Tian G, Yu J, Huang Z, Luo J, Chen D. 2017. Dietary pea fiber increases diversity of colonic methanogens of pigs with a shift from Methanobrevibacter to Methanomassiliicoccus-like genus and change in numbers of three hydrogenotrophs. BMC Microbiol [Internet]. [accessed 2024 Jan 18] 17(1):1–11. https://doi.org/10.1186/S12866-016-0919-9/FIGURES/6
Luo Y heng, Li H, Luo J qiu, Zhang K ying. 2013. Yeast-Derived β-1,3-Glucan Substrate Significantly Increased the Diversity of Methanogens During In vitro Fermentation of Porcine Colonic Digesta. J Integr Agric. 12(12):2229–2234. https://doi.org/10.1016/S2095-3119(13)60381-0
De Maesschalck C, Van Immerseel F, Eeckhaut V, De Baere SD, Cnockaert M, Croubels S, Haesebrouck F, Ducatelle R, Vandamme P. 2014. Faecalicoccus acidiformans gen. nov., Sp. nov., Isolated from the chicken caecum, And reclassification of streptococcus pleomorphus (barnes et al. 1977), Eubacterium biforme (eggerth 1935) and eubacterium cylindroides (cato et al. 1974) as faecalicoccus pleomorphus comb. nov., Holdemanella biformis gen. nov., Comb. nov. and faecalitalea cylindroides gen. nov., Comb. nov., Respectively, Within the family erysipelotrichaceae. Int J Syst Evol Microbiol [Internet]. [accessed 2024 Jan 18] 64(Pt_11):3877–3884. https://doi.org/10.1099/IJS.0.064626-0/CITE/REFWORKS
Martin E, Klug K, Frischmann A, Busse HJ, Kämpfer P, Jäckel U. 2011. Jeotgalicoccus coquinae sp. nov. and Jeotgalicoccus aerolatus sp. nov., isolated from poultry houses. Int J Syst Evol Microbiol [Internet]. [accessed 2024 Jan 18] 61(2):237–241. https://doi.org/10.1099/IJS.0.021675-0/CITE/REFWORKS
Masella AP, Bartram AK, Truszkowski JM, Brown DG, Neufeld JD. 2012. PANDAseq: Paired-end assembler for illumina sequences. BMC Bioinformatics [Internet]. [accessed 2023 Oct 2] 13(1):1–7. https://doi.org/10.1186/1471-2105-13-31/FIGURES/4
McOrist S, Keller L, McOrist AL. 2003. Search for Lawsonia intracellularis and Bilophila wadsworthia in malabsorption-diseased chickens. Can J Vet Res [Internet]. [accessed 2024 Jan 18] 67(3):232. /pmc/articles/PMC227059/
Medvecky M, Cejkova D, Polansky O, Karasova D, Kubasova T, Cizek A, Rychlik I. 2018. Whole genome sequencing and function prediction of 133 gut anaerobes isolated from chicken caecum in pure cultures. BMC Genomics [Internet]. [accessed 2024 Jan 18] 19(1):1–15. https://doi.org/10.1186/S12864-018-4959-4/FIGURES/5
Memon FU, Yang Y, Zhang G, Leghari IH, Lv F, Wang Y, Laghari F, Khushk FA, Si H. 2022. Chicken Gut Microbiota Responses to Dietary Bacillus subtilis Probiotic in the Presence and Absence of Eimeria Infection. Microorganisms [Internet]. [accessed 2022 Nov 21] 10(8). https://doi.org/10.3390/MICROORGANISMS10081548
de Menezes CLA, Boscolo M, da Silva R, Gomes E, da Silva RR. 2023. The degradation of chicken feathers by Ochrobactrum intermedium results in antioxidant and metal chelating hydrolysates and proteolytic enzymes for staphylococcal biofilm dispersion. 3 Biotech [Internet]. [accessed 2024 Mar 13] 13(6):1–9. https://doi.org/10.1007/S13205-023-03619-7/METRICS
Mkaouar H, Mariaule V, Rhimi S, Hernandez J, Kriaa A, Jablaoui A, Akermi N, Maguin E, Lesner A, Korkmaz B, Rhimi M. 2021. Gut Serpinome: Emerging Evidence in IBD. Int J Mol Sci 2021, Vol 22, Page 6088 [Internet]. [accessed 2024 Mar 14] 22(11):6088. https://doi.org/10.3390/IJMS22116088

Nikolenko SI, Korobeynikov AI, Alekseyev MA. 2013. BayesHammer: Bayesian clustering for error correction in single-cell sequencing. BMC Genomics [Internet]. [accessed 2023 Oct 2] 14(1):1–11. https://doi.org/10.1186/1471-2164-14-S1-S7/TABLES/3
Oakley BB, Lillehoj HS, Kogut MH, Kim WK, Maurer JJ, Pedroso A, Lee MD, Collett SR, Johnson TJ, Cox NA. 2014. The chicken gastrointestinal microbiome. FEMS Microbiol Lett. 360(2):100–112. https://doi.org/10.1111/1574-6968.12608
Oerskovia merdavium Sa2CUA9 | DSM 112358 | BacDiveID:169777. [accessed 2024 Jan 18]. https://bacdive.dsmz.de/strain/169777
Parks DH, Imelfort M, Skennerton CT, Hugenholtz P, Tyson GW. 2015. CheckM: assessing the quality of microbial genomes recovered from isolates, single cells, and metagenomes. Genome Res [Internet]. [accessed 2023 Dec 25] 25(7):1043–1055. https://doi.org/10.1101/GR.186072.114
Poudel B, Shterzer N, Sbehat Y, Ben-Porat N, Rakover M, Tovy-Sharon R, Wolicki D, Rahamim S, Bar-Shira E, Mills E. 2022. Characterizing the chicken gut colonization ability of a diverse group of bacteria. Poult Sci. 101(11):102136. https://doi.org/10.1016/J.PSJ.2022.102136
Purty S, Saranathan R, Prashanth K, Narayanan K, Asir J, Sheela Devi C, Kumar Amarnath S. 2013. The expanding spectrum of human infections caused by Kocuria species: a case report and literature review . Emerg Microbes Infect. 2(1):1–1. https://doi.org/10.1038/EMI.2013.93
Qiu M, Hu J, Peng H, Li B, Xu J, Song X, Yu C, Zhang Z, Du X, Bu G, et al. 2022. Research Note: The gut microbiota varies with dietary fiber levels in broilers. Poult Sci. 101(7):101922. https://doi.org/10.1016/J.PSJ.2022.101922
Quast C, Pruesse E, Yilmaz P, Gerken J, Schweer T, Yarza P, Peplies J, Glöckner FO. 2013. The SILVA ribosomal RNA gene database project: improved data processing and web-based tools. Nucleic Acids Res [Internet]. [accessed 2023 Oct 2] 41(D1):D590–D596. https://doi.org/10.1093/NAR/GKS1219
Rakoff-Nahoum S, Foster KR, Comstock LE. 2016. The evolution of cooperation within the gut microbiota. Nat 2015 5337602 [Internet]. [accessed 2024 Jan 18] 533(7602):255–259. https://doi.org/10.1038/nature17626
Rawlings ND, Barrett AJ, Finn R. 2016. Twenty years of the MEROPS database of proteolytic enzymes, their substrates and inhibitors. Nucleic Acids Res [Internet]. [accessed 2023 Dec 25] 44(D1):D343–D350. https://doi.org/10.1093/NAR/GKV1118
Reyer H, Sjöberg PJR, Oster M, Wubuli A, Murani E, Ponsuksili S, Wolf P, Wimmers K. 2021. Mineral phosphorus supply in piglets impacts the microbial composition and phytate utilization in the large intestine. Microorganisms [Internet]. [accessed 2024 Mar 13] 9(6):1197. https://doi.org/10.3390/MICROORGANISMS9061197/S1
Ricke SC, Dittoe DK, Olson EG. 2022. Microbiome applications for laying hen performance and egg production. Poult Sci. 101(5):101784. https://doi.org/10.1016/J.PSJ.2022.101784
Rios-Covian D, Salazar N, Gueimonde M, de los Reyes-Gavilan CG. 2017. Shaping the metabolism of intestinal Bacteroides population through diet to improve human health. Front Microbiol. 8(MAR):247826. https://doi.org/10.3389/FMICB.2017.00376/BIBTEX
Rognes T, Flouri T, Nichols B, Quince C, Mahé F. 2016. VSEARCH: A versatile open source tool for metagenomics. PeerJ [Internet]. [accessed 2023 Oct 2] 2016(10):e2584. https://doi.org/10.7717/PEERJ.2584/FIG-7
Le Roy CI, Mappley LJ, La Ragione RM, Woodward MJ, Claus SP. 2015. Brachyspira pilosicoli -induced avian intestinal spirochaetosis . Microb Ecol Heal Dis [Internet]. [accessed 2024 Mar 13] 26(0). https://doi.org/10.3402/MEHD.V26.28853
Ruddon RW, Arbor A. 2010. Progress in molecular biology and translational science. Preface. Prog Mol Biol Transl Sci [Internet]. [accessed 2024 Mar 13] 95(C). https://doi.org/10.1016/B978-0-12-385071-3.00012-5
Rychlik I. 2020. Composition and Function of Chicken Gut Microbiota. Anim 2020, Vol 10, Page 103. 10(1):103. https://doi.org/10.3390/ANI10010103
Sakamoto M, Lan PTN, Benno Y. 2007. Barnesiella viscericola gen. nov., sp. nov., a novel member of the family Porphyromonadaceae isolated from chicken caecum. Int J Syst Evol

Microbiol [Internet]. [accessed 2024 Jan 18] 57(2):342–346. https://doi.org/10.1099/IJS.0.64709-0/CITE/REFWORKS
Santos RG, Hurtado R, Gomes LGR, Profeta R, Rifici C, Attili AR, Spier SJ, Giuseppe M, Morais-Rodrigues F, Gomide ACP, et al. 2020. Complete genome analysis of Glutamicibacter creatinolyticus from mare abscess and comparative genomics provide insight of diversity and adaptation for Glutamicibacter. Gene. 741:144566. https://doi.org/10.1016/J.GENE.2020.144566
Scheutz F, Strockbine NA. 2005. Bergey's Manual of Systematic Bacteriology: Volume 2: The Proteobacteria. [place unknown]: Springer: New York, NY, USA.
Schirmer M, Ijaz UZ, D'Amore R, Hall N, Sloan WT, Quince C. 2015. Insight into biases and sequencing errors for amplicon sequencing with the Illumina MiSeq platform. Nucleic Acids Res [Internet]. [accessed 2023 Oct 2] 43(6):e37–e37. https://doi.org/10.1093/NAR/GKU1341
Schreuder J, Velkers FC, Bossers A, Bouwstra RJ, de Boer WF, van Hooft P, Stegeman JA, Jurburg SD. 2021. Temporal Dynamics of Cloacal Microbiota in Adult Laying Chickens With and Without Access to an Outdoor Range. Front Microbiol. 11:626713. https://doi.org/10.3389/FMICB.2020.626713/BIBTEX
Schreuder J, Velkers FC, Bouwstra RJ, Beerens N, Stegeman JA, de Boer WF, van Hooft P, Elbers ARW, Bossers A, Jurburg SD. 2020. An observational field study of the cloacal microbiota in adult laying hens with and without access to an outdoor range. Anim Microbiome [Internet]. [accessed 2024 Mar 13] 2(1):1–11. https://doi.org/10.1186/S42523-020-00044-6/FIGURES/6
Selengut JD, Haft DH, Davidsen T, Ganapathy A, Gwinn-Giglio M, Nelson WC, Richter AR, White O. 2007. TIGRFAMs and Genome Properties: tools for the assignment of molecular function and biological process in prokaryotic genomes. Nucleic Acids Res [Internet]. [accessed 2023 Dec 25] 35(suppl_1):D260–D264. https://doi.org/10.1093/NAR/GKL1043
Singh KM, Shah T, Deshpande S, Jakhesara SJ, Koringa PG, Rank DN, Joshi CG. 2012. High through put 16S rRNA gene-based pyrosequencing analysis of the fecal microbiota of high FCR and low FCR broiler growers. Mol Biol Rep [Internet]. [accessed 2024 Mar 13] 39(12):10595–10602. https://doi.org/10.1007/S11033-012-1947-7/METRICS
Sorokin DY, Lücker S, Vejmelkova D, Kostrikina NA, Kleerebezem R, Rijpstra WIC, Sinninghe Damsté JS, Le Paslier D, Muyzer G, Wagner M, et al. 2012. Nitrification expanded: discovery, physiology and genomics of a nitrite-oxidizing bacterium from the phylum Chloroflexi. ISME J 2012 612 [Internet]. [accessed 2024 Jan 18] 6(12):2245–2256. https://doi.org/10.1038/ismej.2012.70
Sorokin DY, Vejmelkova D, Lücker S, Streshinskaya GM, Rijpstra WIC, Sinninghe Damsté JS, Kleerbezem R, van Loosdrecht M, Muyzer G, Daims H. 2014. Nitrolancea hollandica gen. nov., sp. nov., a chemolithoautotrophic nitrite-oxidizing bacterium isolated from a bioreactor belonging to the phylum Chloroflexi. Int J Syst Evol Microbiol [Internet]. [accessed 2024 Jan 18] 64(PART 6):1859–1865. https://doi.org/10.1099/IJS.0.062232-0/CITE/REFWORKS
Sun B, Hou L, Yang Y. 2021. The Development of the Gut Microbiota and Short-Chain Fatty Acids of Layer Chickens in Different Growth Periods. Front Vet Sci. 8:666535. https://doi.org/10.3389/FVETS.2021.666535/BIBTEX
Tong L, Wang W, Ren S, Wang Jianling, Wang Jie, Qu Y, Addoma Adam FE, Li Z, Gao X. 2022. The 16S rRNA Gene Sequencing of Gut Microbiota in Chickens Infected with Different Virulent Newcastle Disease Virus Strains. Anim 2022, Vol 12, Page 2558. 12(19):2558. https://doi.org/10.3390/ANI12192558
Ty M, Taha-Abdelaziz K, Demey V, Castex M, Sharif S, Parkinson J. 2022. Performance of distinct microbial based solutions in a Campylobacter infection challenge model in poultry. Anim Microbiome [Internet]. [accessed 2024 Jan 18] 4(1):1–19. https://doi.org/10.1186/S42523-021-00157-6/FIGURES/4
Uritskiy G V., Diruggiero J, Taylor J. 2018. MetaWRAP - A flexible pipeline for genome-resolved metagenomic data analysis 08 Information and Computing Sciences 0803 Computer Software 08 Information and Computing Sciences 0806 Information Systems. Microbiome [Internet]. [accessed 2023 Dec 25] 6(1):1–13. https://doi.org/10.1186/S40168-

018-0541-1/FIGURES/7
Varriale L, Coretti L, Dipineto L, Green BD, Pace A, Lembo F, Menna LF, Fioretti A, Borrelli L. 2022. An Outdoor Access Period Improves Chicken Cecal Microbiota and Potentially Increases Micronutrient Biosynthesis. Front Vet Sci. 9:904522. https://doi.org/10.3389/FVETS.2022.904522/BIBTEX
Wang L, Chai B. 2022. Fate of Antibiotic Resistance Genes and Changes in Bacterial Community With Increasing Breeding Scale of Layer Manure. Front Microbiol. 13:857046. https://doi.org/10.3389/FMICB.2022.857046/BIBTEX
Wang L, Zhang F, Li H, Yang Shenglin, Chen X, Long S, Yang Shenghong, Yang Y, Wang Z. 2023. Metabolic and inflammatory linkage of the chicken cecal microbiome to growth performance. Front Microbiol. 14:1060458. https://doi.org/10.3389/FMICB.2023.1060458/BIBTEX
Wang Yang, Xu C, Zhang R, Chen Y, Shen Y, Hu F, Liu D, Lu J, Guo Y, Xia X, et al. 2020. Changes in colistin resistance and mcr-1 abundance in Escherichia coli of animal and human origins following the ban of colistin-positive additives in China: an epidemiological comparative study. Lancet Infect Dis [Internet]. [accessed 2023 Aug 17] 20(10):1161–1171. https://doi.org/10.1016/S1473-3099(20)30149-3
Wen C, Gou Q, Gu S, Huang Q, Sun C, Zheng J, Yang N. 2023. The cecal ecosystem is a great contributor to intramuscular fat deposition in broilers. Poult Sci. 102(4):102568. https://doi.org/10.1016/J.PSJ.2023.102568
Wongkuna S, Ghimire S, Chankhamhaengdecha S, Janvilisri T, Scaria J. 2021. Description of Collinsella avium sp. nov., a new member of the Collinsella genus isolated from the ceacum of feral chicken. New Microbes New Infect. 42:100902. https://doi.org/10.1016/J.NMNI.2021.100902
Wu Y, Zhang H, Zhang R, Cao G, Li Q, Zhang B, Wang Y, Yang C. 2021. Serum metabolome and gut microbiome alterations in broiler chickens supplemented with lauric acid. Poult Sci. 100(9):101315. https://doi.org/10.1016/J.PSJ.2021.101315
Wu YW, Simmons BA, Singer SW. 2016. MaxBin 2.0: an automated binning algorithm to recover genomes from multiple metagenomic datasets. Bioinformatics [Internet]. [accessed 2023 Dec 25] 32(4):605–607. https://doi.org/10.1093/BIOINFORMATICS/BTV638
Xi L, Wen X, Jia T, Han J, Qin X, Zhang Y, Wang Z. 2023. Comparative study of the gut microbiota in three captive Rhinopithecus species. BMC Genomics [Internet]. [accessed 2024 Mar 13] 24(1):1–11. https://doi.org/10.1186/S12864-023-09440-Z/FIGURES/6
Xi Y, Shuling N, Kunyuan T, Qiuyang Z, Hewen D, ChenCheng G, Tianhe Y, Liancheng L, Xin F. 2019. Characteristics of the intestinal flora of specific pathogen free chickens with age. Microb Pathog. 132:325–334. https://doi.org/10.1016/J.MICPATH.2019.05.014
Yang J, Li Y, Wen Z, Liu W, Meng L, Huang H. 2021. Oscillospira - a candidate for the next-generation probiotics. Gut Microbes [Internet]. [accessed 2024 Jan 18] 13(1). https://doi.org/10.1080/19490976.2021.1987783
Yang WY, Chou CH, Wang C. 2022. The effects of feed supplementing Akkemansia muciniphila on incidence, severity, and gut microbiota of necrotic enteritis in chickens. Poult Sci. 101(4):101751. https://doi.org/10.1016/J.PSJ.2022.101751
Yang Y, Ashworth AJ, Willett C, Cook K, Upadhyay A, Owens PR, Ricke SC, DeBruyn JM, Moore PA. 2019. Review of Antibiotic Resistance, Ecology, Dissemination, and Mitigation in U.S. Broiler Poultry Systems. Front Microbiol [Internet]. [accessed 2021 Feb 15] 10. https://doi.org/10.3389/fmicb.2019.02639
Ye Z, Zhang N, Wu C, Zhang X, Wang Q, Huang X, Du L, Cao Q, Tang J, Zhou C, et al. 2018. A metagenomic study of the gut microbiome in Behcet's disease. Microbiome [Internet]. [accessed 2024 Jan 18] 6(1):1–13. https://doi.org/10.1186/S40168-018-0520-6/TABLES/1
Yeoman CJ, Chia N, Jeraldo P, Sipos M, Goldenfeld ND, White BA. 2012. The microbiome of the chicken gastrointestinal tract. Anim Heal Res Rev. 13(1):89–99. https://doi.org/10.1017/S1466252312000138
Yusuf I, Garba L, Shehu MA, Oyiza AM, Kabir MR, Haruna M. 2020. Selective biodegradation of recalcitrant black chicken feathers by a newly isolated thermotolerant

bacterium Pseudochrobactrum sp. IY-BUK1 for enhanced production of keratinase and protein-rich hydrolysates. Int Microbiol [Internet]. [accessed 2024 Mar 13] 23(2):189–200. https://doi.org/10.1007/S10123-019-00090-4/METRICS
Zhang H, Yohe T, Huang L, Entwistle S, Wu P, Yang Z, Busk PK, Xu Y, Yin Y. 2018. dbCAN2: a meta server for automated carbohydrate-active enzyme annotation. Nucleic Acids Res [Internet]. [accessed 2023 Dec 25] 46(W1):W95–W101. https://doi.org/10.1093/NAR/GKY418
Zhang X, Hu Y, Ansari AR, Akhtar M, Chen Y, Cheng R, Cui L, Nafady AA, Elokil AA, Abdel-Kafy ESM, Liu H. 2022. Caecal microbiota could effectively increase chicken growth performance by regulating fat metabolism. Microb Biotechnol [Internet]. [accessed 2024 Jan 18] 15(3):844–861. https://doi.org/10.1111/1751-7915.13841
Zhou Z, Tran PQ, Breister AM, Liu Y, Kieft K, Cowley ES, Karaoz U, Anantharaman K. 2022. METABOLIC: high-throughput profiling of microbial genomes for functional traits, metabolism, biogeochemistry, and community-scale functional networks. Microbiome [Internet]. [accessed 2023 Dec 25] 10(1):1–22. https://doi.org/10.1186/S40168-021-01213-8/FIGURES/10
